# Proceedings of the 4th National Big Data Health Science Conference

**DOI:** 10.1186/s12919-023-00281-y

**Published:** 2023-11-23

## I1 Introduction: Proceedings of the 4^th^ National Big Data Health Science Conference

### Xiaoming Li^1,2^, Bankole Olatosi^1,3^, Miranda Nixon^1,2^

#### ^1^Big Data Health Science Center, University of South Carolina, Columbia, SC, USA; ^2^Department of Health Promotion, Education, and Behavior, Arnold School of Public Health, University of South Carolina, Columbia, SC, USA; ^3^Department of Health Services Policy and Management, Arnold School of Public Health, University of South Carolina, Columbia, SC, USA

*BMC Proceedings 2023*, **17(Suppl 19):**I1

This proceeding contains some of the oral and poster presentations from the 4^th^ National Big Data Health Science Conference (Columbia, SC, Feb 10-11, 2023) that was organized by the University of South Carolina Big Data Health Science Center (BDHSC; https://bigdata.sc.edu/). The BDHSC is an interdisciplinary enterprise that promotes and supports Big Data health science research through capacity development, academic training, professional development, community engagement and methodological advancement. Starting in 2020, this recurring annual national conference has four primary goals: 1) Create a multidisciplinary scientific venue for the exchange of new concepts, methods, and results to encourage the sharing of theoretical, methodological, and substantive knowledge from Big Data health science research; 2) Identify new issues that are, to date, understudied in this area, and then generate, promote, and support innovation in Big Data health science; 3) Expand impact and scholarly excellence by producing new and interdisciplinary publications; and 4) Promote inclusive excellence in training and mentoring opportunities by engaging and supporting underrepresented junior investigators and students in the conference. This proceeding reflects our efforts in achieving those goals, especially those related to the scientific knowledge creation and dissemination.

The U.S. healthcare industry is a complex adaptive system^1^ that is constantly changing as a result of technological advancements, aging populations, changing disease patterns, increasing noncommunicable diseases, rising costs, new discoveries for the treatment of diseases, political reforms and policy initiatives.^2^ Moreover, big societal outbreaks and events like COVID-19 and climate change put additional and unexpected burdens on healthcare. Novel strategies are needed to bring about much needed change to the complex and evolving U.S. healthcare system. The exponential growth of healthcare data from various sources and the emergence of advanced information, communication and computational technologies, collectively referred to as “Big Data analytics” (or data science), offers an invaluable opportunity to improve the quality and efficiency of healthcare.^3,4^ The NIH-Wide Strategic Plan for FY 2021-2025 leverages data science as one of its cross-cutting themes.^5^ Further, NIH’s first Strategic Plan for Data Science^6^ released in June 2018 suggests that the Big Data approach will advance uniquely our understanding of disease prevention, identification, control and treatment in the coming decades and will be key to reducing national and global health disparities.

While other health related Big Data conferences offer a deeper dive into their respective areas such as artificial intelligence,^7^ machine learning,^8,9,10^ and health information technology,^11^ the BDHS Conference differs in its approach by filling an important role in promoting Big Data health science and serving as a multidisciplinary platform by bringing all stakeholders together to focus on addressing healthcare challenges and advancing our understanding of the unique solutions Big Data offers the nation’s healthcare system. To the best of our knowledge, there are few conferences in which the sole theme is fully focused on Big Data health science. Through its menu of plenary (keynote) sessions, participatory breakout sessions organized by content of Big Data (electronic health records [EHR] data, geospatial data, social media data, genomic data, and artificial intelligence [AI] for sensing and diagnosis), panel discussions, hands-on workshops, special sessions (e.g., NIH grantee session, NIH R25/T35 trainee session, underrepresented minority luncheon), poster sessions, and networking opportunities, this annual conference moves beyond offering one piece of the puzzle and seeks to bridge the gaps between Big Data health science’s many crucial stakeholders including the general public, research communities, government agencies and healthcare providers.

The use of Big Data analytics or data science in healthcare has already presented promising results for the generation of value for all healthcare stakeholders in several contexts. However, more technological, organizational, multidisciplinary, and collaborative connectivity among stakeholders from academia, government and industry is critical to realizing the full potential of data science in healthcare. To address these demands and challenges, our conference will continue to provide a multidisciplinary forum for the discussion of state-of-the art advancements in Big Data health science; promote open discussions surrounding critical questions in Big Data health science with particular emphasis on emerging methods which may contribute to the future of healthcare; and facilitate the exchange of ideas and communication of findings that could shape the future of Big Data health science with a meaningful partnership between academia, government, industry and healthcare systems alike.

We also would like to take this opportunity to thank the USC administration, the BDHSC Steering Committee, the BDHSC National Advisory Committee, and the Conference Planning Committee for their leadership, support, and efforts to make the conference a reality and success. We also want to thank all the authors who have provided their consent to publish their abstracts in this inaugural issue of our conference proceedings. We look forward to the participation of faculty members, graduate students, postdoctoral researchers, healthcare practitioners, clinical researchers and representatives from industry, government agencies and non-profit organizations in our future conferences (https://www.sc-bdhs-conference.org/).

**References**

1. Braithwaite J. Changing how we think about healthcare improvement. BMJ. 2018;361:k2014. Published 2018 May 17. doi:10.1136/bmj.k2014.
2. Nilsen P, Seing I, Ericsson C, Birken SA, Schildmeijer K. Characteristics of successful changes in health care organizations: an interview study with physicians, registered nurses and assistant nurses. BMC Health Serv Res. 2020;20(1):147. Published 2020 Feb 27. doi:10.1186/s12913-020-4999-8.
3. Schneeweiss S. Learning from big health care data. N Engl J Med. 2014;370(23):2161-2163. doi:10.1056/NEJMp1401111.
4. Raghupathi W, Raghupathi V. Big data analytics in healthcare: promise and potential. Health Inf Sci Syst. 2014;2:3. Published 2014 Feb 7. doi:10.1186/2047-2501-2-3.
5. National Institutes of Health. NIH-Wide Strategic Plan for FY 2021-2025. Accessed on August 25, 2022. https://www.nih.gov/sites/default/files/about-nih/strategic-plan-fy2021-2025-508.pdf.
6. National Institutes of Health. Strategic Plan for Data Science. Accessed on August 25, 2022. https://datascience.nih.gov/sites/default/files/NIH_Strategic_Plan_for_Data_Science_Final_508.pdf
7. Data Summit (Boston), May 17-19, 2022. The Data Management and Analytics Conference. Accessed on May 25, 2022. Available at https://www.dbta.com/DataSummit/2022/Default.aspx.
8. MLDM 2022. 17th International Conference on Machine Learning and Data Mining (New York), June 16-21, 2022. Accessed on May 25, 2022. http://www.mldm.de/.
9. Predictive Analytics World Business. Machine Learning Week (Las Vegas), June 19-24, 2022. Accessed on May 25, 2022. https://www.predictiveanalyticsworld.com/machinelearningweek/.
10. IEEE. 2022 IEEE International Conference on Big Data (Osaka, Japan), December 17-20, 2022. Accessed on August 25, 2022. https://www.himss.org/global-conference.
11. Healthcare Information and Management Systems Society. HIMSS 2023 (Chicago), April 17-21, 2023. Accessed on August 25, 2022. https://www.himss.org/global-conference.

## O1 PDBMine, a tool for improving the performance AlphaFold

### Hamed Abdollahi^1^, Niharika Pandala^2^, Homayoun Valafar^1^

#### ^1^Department of Computer Science and Engineering, University of South Carolina, Columbia, SC, USA; ^2^Department of Bioengineering, University of Texas at Dallas, Richardson, TX, USA

##### **Correspondence:** Homayoun Valafar (HOMAYOUN@cse.sc.edu)

*BMC Proceedings 2023*, **17(Suppl 19):**O1

**Study Objectives:** There is a direct relationship between the biological characteristics and functions of a protein and its three-dimensional conformation, which is influenced by its genetic sequence and changes in environmental conditions. Currently, experimental approaches provide the highest quality, accuracy, and reliability in elucidating protein structures, as compared to computational methods. However, in recent years, computational modeling techniques have become indispensable for studying protein structures. One such computational model is AlphaFold, which is one of the most prominent neural network-based models that predicts protein structure solely based on the peptide sequence of amino acids. Although very successful, AlphaFold has demonstrated certain deficiencies in structure determination of proteins. The objective of this work is to develop data analytics techniques in order to identify and correct the points of structural error by AlphaFold.

**Methods:** AlphaFold results of modeling protein templates that are the targets of critical assessment of structure prediction (CASP) community experiment XIV and XV have been extracted from (“https://predictioncenter.org/casp14/results.cgi”) along with the corresponding experimentally determined PDB files. In addition, we have developed PDBMine algorithm, which utilizes PDB (“https://www.rcsb.org/”) data to provide an approach for the validation and modeling of protein structures. We use PDBMine^1^ as a method of assessing the plausibility of model structures provided by any modeling technique such as AlphaFold. In this experiment we used target 1024 for our studies.

**Results:** Figure 1 below illustrates dihedral angles obtained from three different sources. The PDBMine results for specific residues (residues 98 and 131) are plotted in blue, and the corresponding dihedral angles from the X-ray and AlphaFold structures are illustrated in green and red respectively. Figure 1 (a) shows an agreement between PDBMine and X-ray Structure, and disagreement between PDBMine and AlphaFold for residue 98 with local structure violations. On the other hand, residue 131 with correct local geometry demonstrate agreement between PDBMine, X-ray Structure, and AlphaFold structures.

**Discussion:** PDBMine can be used as a post-processing technique to improve the performance of AlphaFold. First, it can be used to identify the points of structural divergence and second, to correct any errors in modeled structures by AlphaFold. PDBMine can also help to accept of decline a model produce by AlphaFold in order to increase the reliability of the modeled structures.

**References**

1. Cole, C., Ott, C., Valdes, D., & Valafar, H. (2019, December). PDBMine: A reformulation of the protein data bank to facilitate structural data mining. In 2019 International Conference on Computational Science and Computational Intelligence (CSCI) (pp. 1458-1463). IEEE.

**Fig. 1 (abstract O1).**
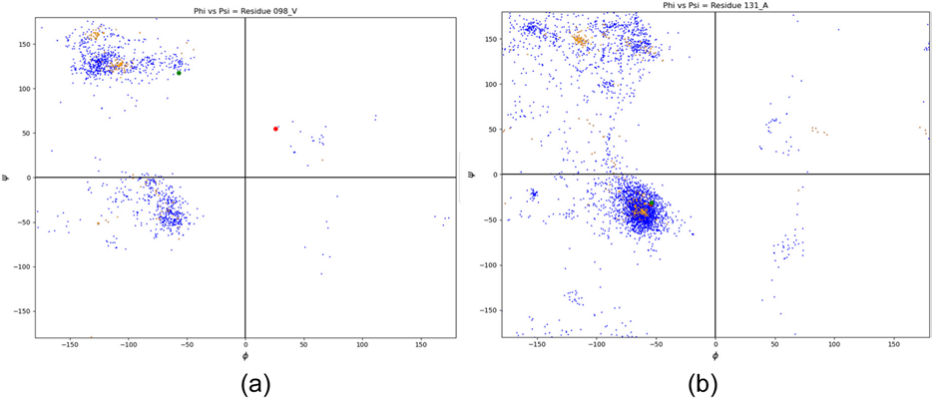
PDBMine results for a) residue 98 and b) residue 131 as examples of incorrect and correct local structures respectively

## O2 Big data health science case competition: impact and outcomes

### Dilek Akgun^1,3^, Audrey Auen^2,3^, Bankole Olatosi^2,3^

#### ^1^Department of Integrated Information Technology, College of Engineering and Computing, University of South Carolina, Columbia, SC, USA; ^2^Department of Health Services Policy and Management, Arnold School of Public Health, University of South Carolina, Columbia, SC, USA; ^3^Big Data Health Science Center, University of South Carolina, Columbia, SC, USA

##### **Correspondence:** Dilek Akgun (Akgun@mailbox.sc.edu)

*BMC Proceedings 2023*, **17(Suppl 19):**O2

**Introduction:** Case competitions are a useful form of experiential learning in higher education that allows students to apply the analytical competencies gained in the classroom to real-world challenges. In addition to learning and applying skills, case competitions enable students to develop and enhance problem-solving, critical thinking, teamwork, communication skills, and social capital development.^1^ Case competitions also helps students develop public speaking and presentation skills that increase self-confidence.

Big data case competitions specifically provide a great platform for students to demonstrate the real-world application of data analytics and data science skills to problems. Students work on a challenging problem under time pressure, gain experience working with real data, and present findings to a panel of judges. This is a valuable experience for students who are interested in a data science career. Students must quickly identify the key problems in a case, analyze the data, and develop and present solutions. These competitions can be very challenging, as students are typically given a short amount of time to analyze the data and come up with a solution. However, they can also be very rewarding, as students gain valuable skills and experience that can help them in their future careers. Literature shows that students who take part in case competitions express motivation and pride at having the opportunity to represent their universities, demonstrate teamwork, and attain favorable reactions from the judges.^2^ Prior research also suggests that case competitions offer students a venue to work in diverse teams during this experiential activity.^3^ This abstract discusses how using a healthcare data specific case competition as a form of experiential learning can help solve real-world problems, and presents its outcomes.

**Innovation** *:* As an important component of the Annual National Big Data Health Science (BDHS) Conference^4^, the Annual Big Data Health Science Case Competition^5^ provides enthusiastic teams of graduate and undergraduate students with the opportunity to apply competencies in data science to the analyses of real-world problems using large healthcare datasets. Participating students are presented with a real-world healthcare data challenge and have a limited amount of time to analyze the problem, develop a data-driven solution, and present their findings to a national panel of academic and industry judges.

**Process and Structure:** The competition is designed to be a data science experience in healthcare open to all eligible undergraduate and graduate students. The Case Competition is made up of two rounds: the preliminary round and final round. Participating teams are given the same problem and datasets to solve a challenging problem using a data science approach within 24 hours. A panel of national industry and academic experts judge the presentations based on the teams’ use of the full analytics process, from framing the problem to method selection, data use, model building, implementation, and innovation.

**Outcomes:** Since it began four years ago, 95 teams totaling 279 students across 32 universities in the United States and 1 university in China have participated in the annual case competition. Table 1 presents the number of participating institutions and teams and the total number of students who competed in the BDHS case competition. Figure 1 shows the distribution of participating students’ degrees.

**Conclusion:** Overall, case competitions are a valuable learning experience for students in higher education. More specifically, big data case competitions are a great way to learn and apply data science skills to real world healthcare problems. BDHSC case competitions also help students develop a strong foundation in data science applied to healthcare, including understanding the different types of data, how to collect and clean data, and how to use statistical and machine learning techniques to analyze data. They are a great way for students to improve their critical thinking, problem-solving, and teamwork skills.

**References**

1. Gamble, E., & Jelley, R. (2014). The case for competition: Learning about evidence-based management through case competition. Academy of Management Learning & Education, 433-445.
2. Kinzie, M., Hrabe, M., & Larsen, V. (1997). Exploring professional practice through an instruction design team case competition. Proceedings of Selected Research and Development Presentations at the 1997 National Convention of the Association for Educational Communications and Technology, (pp. 93-106). Albuquerque, NM
3. Phillips, T.N., & Wood, L.I. (2017). Teaching Diversity through Case Competition.
4. National Big Data Health Science Conference. Available at https://www.sc-bdhs-conference.org/
5. The Annual Big Data Health Science Case Competition. Available at https://bigdata.sc.edu/events/student-case-competition/

**Table 1 (abstract O2).** Competing Teams in the Annual BDHSC Case Competition Across 2020 – 2023

	The Number of		
Year	Participating Institutions	Teams	Students
2020	8	19	56
2021	14	22	64
2022	10	16	48
2023	15	37	111

**Fig. 1 (Abstract O2).**
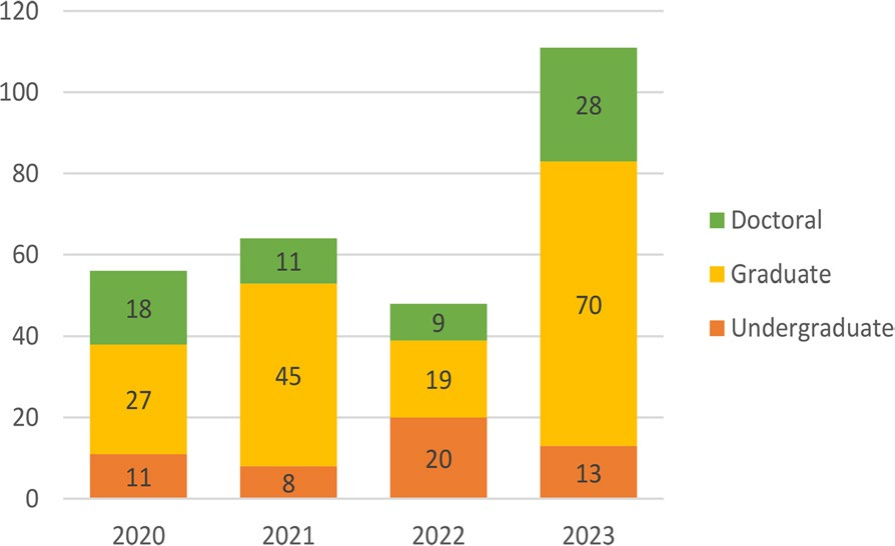
The Annual Case Competition Distribution of Participating Students’ Degrees

## O3 Automatic segmentation of vasculature in computed tomographic angiograms using deep learning

### Alireza Bagheri Rajeoni^1^, Breanna Pederson^2^, Susan M. Lessner^2^, Homayoun Valafar^1^

#### ^1^Department of Computer Science and Engineering, College of Engineering and Computing, University of South Carolina, Columbia, SC, USA; ^2^School of Medicine Columbia, University of South Carolina, Columbia, SC, USA

##### **Correspondence:** Homayoun Valafar (homayoun@cse.sc.edu)

*BMC Proceedings 2023*, **17(Suppl 19):**O3

**Study Objectives:** Numerous chronic diseases, including atherosclerosis and aneurysms are rooted in abnormal changes within the human vascular system. However, the manual examination of medical images, such as computed tomographic angiograms (CTAs), for analyzing the vascular system is a time-consuming and exhaustive task. To tackle this challenge, we propose a deep learning model specifically designed to segment the vascular system in CTA images of patients who undergo surgery for peripheral arterial disease (PAD). Our research focuses on accurately predicting two regions: (1) from the descending thoracic aorta to the iliac bifurcation, and (2) from the descending thoracic aorta to the knees in CTA images, utilizing advanced deep learning techniques.

**Methods:** Using the dataset of 11 patients collected at Prisma health ^1^, we utilized a deep learning algorithm to segment the vascular system. Our model architecture follows an encoder-decoder structure similar to U-net ^2^, incorporating skip connections from the encoder to decoder. Additionally, a Transformer ^3^ is utilized in the bridge between these two main blocks as illustrated in Figure 1. The inputs to the model were images with dimensions of 512x512 and three channels, and the output was a mask with dimensions of 512x512 and one channel. We trained the model using a batch size of 40, for 400 epochs, with a learning rate of 1e-3. During training and validation, we utilized Intersection over Union (IOU) as the evaluation metric, while Dice score was used for testing.

**Results:** During the training and validation process, we achieved average IOU accuracies of 97.3% and 95% for segmenting the vascular system from the descending thoracic aorta to the knees, respectively, in the cross-validation. In the testing dataset, we obtained average Dice accuracies of 93.3% and 83.4% for (1) segmenting the vascular system from the descending thoracic aorta to the iliac bifurcation and (2) from the descending thoracic aorta to the knees, respectively. These results demonstrate the high accuracy and potential clinical usefulness of our trained model. Figure 2 showcases the outcomes achieved by the trained model when segmenting the vascular system in the testing data.

**Discussion:** Accurately capturing and examining the vascular system enables the identification of various pathological conditions like aneurysms and vascular calcification. Moving forward, our primary objective is to improve the accuracy of segmenting and precisely measuring calcification within the vascular system. This progress will significantly enhance diagnostic precision, facilitate proactive treatment, and ultimately lead to better outcomes for patients in the field of vascular health.

**References**

1. Zhao, L., Odigwe, B., Lessner, S., Clair, D., Mussa, F., & Valafar, H. (*2019). Automated Analysis of Femoral Artery Calcification Using Machine Learning Techniques. 2019 International Conference on Computational Science and Computational Intelligence (CSCI)*, 584–589. 10.1109/CSCI49370.2019.00110
2. Ronneberger, O., Fischer, P., & Brox, T. (2015) U-Net: Convolutional Networks for Biomedical Image Segmentation. In N. Navab, J. Hornegger, W. M. Wells, & A. F. Frangi (Eds.), *Medical Image Computing and Computer-Assisted Intervention – MICCAI 2015* (pp. 234–241). Springer International Publishing. 10.1007/978-3-319-24574-4_28
3. Dosovitskiy, A., Beyer, L., Kolesnikov, A., Weissenborn, D., Zhai, X., Unterthiner, T., Dehghani, M., Minderer, M., Heigold, G., Gelly, S., Uszkoreit, J., & Houlsby, N. (2021). *An Image is Worth 16x16 Words: Transformers for Image Recognition at Scale* (arXiv:2010.11929). arXiv. 10.48550/arXiv.2010.11929

**Fig. 1 (abstract O3).**
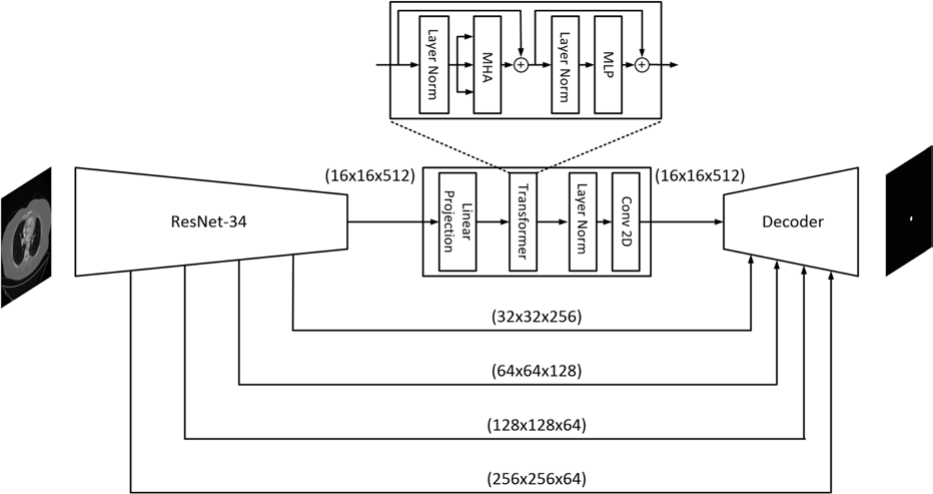
TransONet structure. In the decoder, ResNet-34 is used; in the bridge, a transformer block is used and feeds into the decoder. Skip connection from different stages of encoder samples to decoder to construct the mask

**Fig. 2 (abstract O3).**
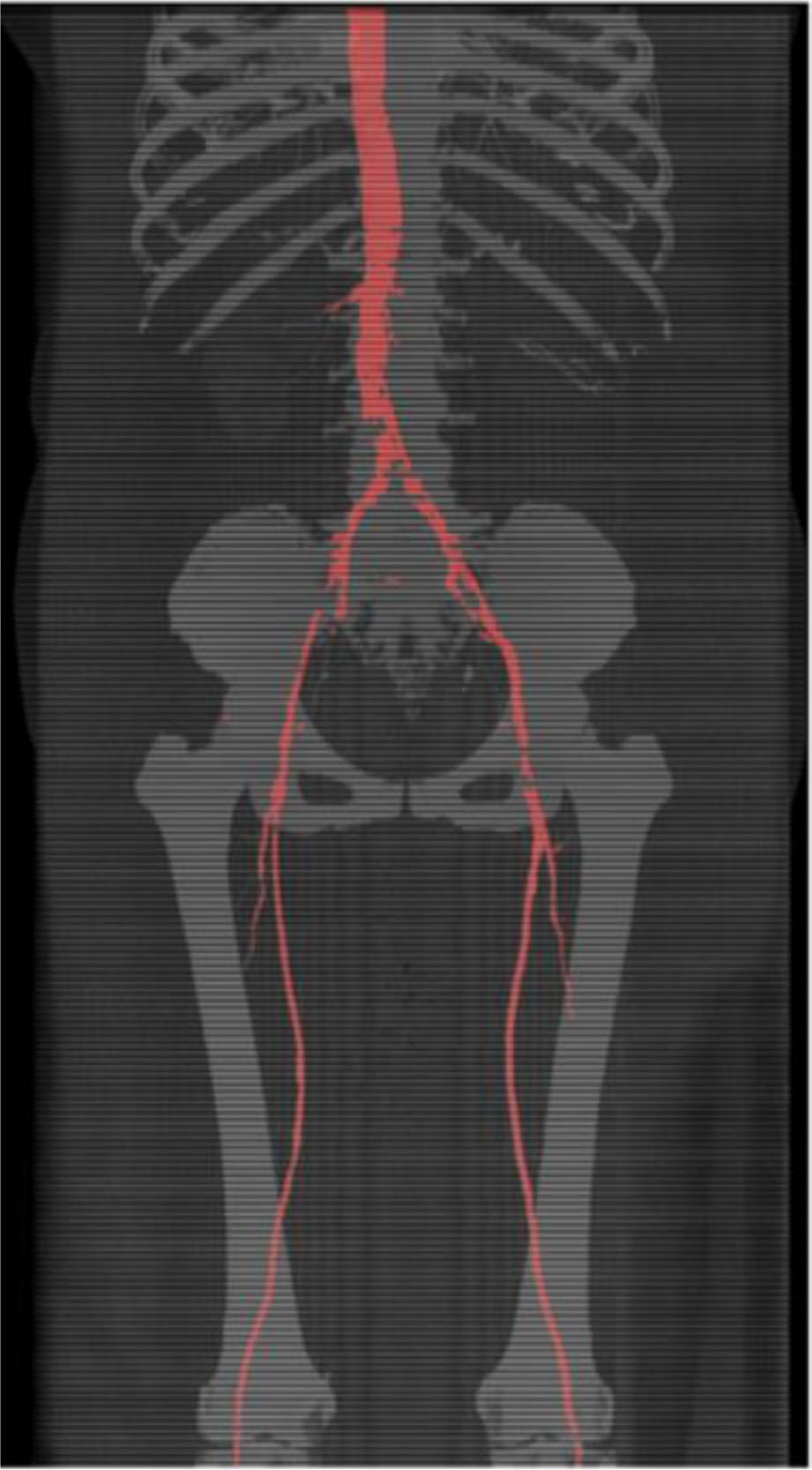
Result of using transOnet on the testing dataset. The red region represents the predicted vascular system. On this specific dataset the model achieved 91.2% Dice accuracy

## O4 On the use of an automated, reproducible binning approach to bring consistency in calibration of predictive models built on electronic health records

### Madhusree Chowdhury, Richard Jordan

#### Health Data Analytics Institute, Dedham, Massachusetts, USA

##### **Correspondence:** Madhusree Chowdhury (madhusree.chowdhury@uconn.edu)

*BMC Proceedings 2023*, **17(Suppl 19):**O4

**Study Objectives:** The rise in usage of electronic health records (EHR) promises the development of predictive algorithms for adverse health outcomes. While much focus has been on the accuracy/discrimination of models, calibration—the agreement between the estimated and true risk of an outcome—is also important in clinical settings.

Calibration is assessed via reliability diagrams and quantified through the miscalibration component of Brier Score decomposition. The methodology relies on binning the predicted probabilities. Historically, the number of bins is selected in an ad hoc fashion. Changing the number alters the appearance of the reliability plot and the values of the metrics in the decomposition. The CORP approach^1^, which generates optimally binned reliability diagrams in an automated way, aims to solve this problem of instability.

Our objective is to assess the effectiveness of CORP and compare the calibration and discrimination of several machine learning methods for predicting three health outcomes of interest: sepsis, respiratory failure, and mortality.

**Methods:** Our training set is a 5% sample of 2018 national Medicare admission data (n=476,593). Patients with 12 months of part A and part B coverage, without any part C coverage, and age>=18 are considered. The test set is a 5% sample of similar data from 2019 (n = 465,041).

Features include ICD-10^2^ diagnosis codes, CCSR^3^ disease codes, CPT-10^4^ procedure codes, and the age and sex of patients. The ICD, CCSR and CPT variables are considered with 365 and 90-day history.

The models predict the probability of an adverse outcome within 90 days after admission. We investigate performance of XGBoost, LightGBM, and regularized logistic regression models.

Metrics of interest include the Brier Score and the components of its decomposition:

$$Brier\ Score=\frac{1}{N}\sum\limits_{k=1}^K{n}_k{\left({p}_k-{o}_k\right)}^2-\frac{1}{N}\sum\limits_{k=1}^K{n}_k{\left({o}_k-\overline{o}\right)}^2+\overline{o}\left(1-\overline{o}\right)$$

First Term: *Miscalibration*, Second Term: *Discrimination*, Third Term: *Uncertainty*

Here, predicted probabilities are discretized into *K* bins. In bin *k*, *n*_*k*_ is the number of data points, *p*_*k*_ is the average predicted probability and *o*_*k*_ is the average outcome. $\overline{o}$is the average outcome over the population (incidence). The uncertainty is inherent to the data itself, so is independent of the predictive model. Note that two models can have identical Brier Scores yet different miscalibration.

CORP uses the pool-adjacent-violators (PAV) algorithm to optimally select the number of bins and their sizes. To each predicted probability, PAV assigns a calibrated probability under the regularizing constraint of isotonicity and interpolates linearly in between to facilitate comparison with the diagonal corresponding to perfect calibration.

**Results:** Table 1 shows results for the LightGBM mortality model using the standard fixed bin-width calibration approach. The non-monotone variation of the miscalibration as the number of bins increases illustrates the instability mentioned above. Table 2 shows results of the different models for the various outcomes using the CORP approach. LightGBM consistently yields the best results for all the metrics and shows the best calibration by far.

**Discussion:** We have found CORP to be effective in providing stable reliability diagrams and calibration metrics. It is expected that its adaptation in a clinical setting would be valuable and give a rigorous analysis of model predictions.

**References**

1. Dimitriadis T, Gneiting T, Jordan AI. Stable reliability diagrams for probabilistic classifiers. Proc Natl Acad Sci. 2021;118(8). doi:10.1073/pnas.2016191118
2. Centers for Disease Control and Prevention. International Classification of Diseases, Tenth Revision, Clinical Modification (ICD-10-CM) and Procedure Coding System (ICD-10-PCS) Background. Accessed December 10, 2022. Available from: https://www.cdc.gov/nchs/icd/icd10cm_pcs_background.htm
3. Agency for Healthcare Research and Quality. Clinical Classifications Software (CCS) for ICD-10-CM Diagnoses - Beta Version. Accessed December 10, 2022. Available from: https://www.hcup-us.ahrq.gov/toolssoftware/ccsr/dxccsr.jsp
4. National Athletic Trainers' Association. Commonly Used CPT Codes. Accessed December 10, 2022. Available from: https://www.nata.org/practice-patient-care/revenue-reimbursement/general-revenue-reimbursement/commonly-used-cpt-codes

**Table 1 (abstract O4).** Model results for standard fixed bin-width calibration approach

# Bins	Brier Score	Miscalibration	Discrimination
10	0.09620	1.39e-04	0.0227
25	0.09541	6.64e-05	0.0234
100	0.09529	8.93e-05	0.0236
1000	0.09528	3.82e-04	0.0239

**Table 2 (abstract O4).** Model results for CORP approach

Sepsis			
Model	Brier Score	Miscalibration	Discrimination
XGBoost	0.05107	1.03e-04	0.00229
LightGBM	0.05097	4.39e-05	0.00247
Regularized-logistic-regression	0.05127	1.49e-04	0.00230
**Mortality**			
XGBoost	0.09571	2.43e-04	0.0231
LightGBM	0.09528	9.19e-05	0.0234
Regularized-logistic-regression	0.09669	4.37e-04	0.0225
**Respiratory Failure**			
XGBoost	0.05538	7.38e-05	0.00407
LightGBM	0.05338	5.40e-05	0.00427
Regularized-logistic-regression	0.05576	1.11e-04	0.00398

## O5 Using random forest classifier to identify important COVID-19 patient characteristics predicting mortality in South Florida

### Debarshi Datta^1^, Safiya George Dalmida^1^, Laurie Martinez^1^, David Newman^1^, Javad Hashemi^2^, Taghi M. Khoshgoftaar^2^, Connor Shorten^2^, Candice Sareli^3^, Paula Eckardt^3^

#### ^1^Christine E. Lynn College of Nursing, Florida Atlantic University, Boca Raton, FL, USA; ^2^College of Engineering & Computer Science, Florida Atlantic University, Boca Raton, FL, USA; ^3^Memorial Healthcare System, Hollywood, FL, USA

##### **Correspondence:** Debarshi Datta (debarshidatta@outlook.com)

*BMC Proceedings 2023*, **17(Suppl 19):**O5

**Background:** The COVID-19 pandemic continues to create substantial health and economic burdens in the US. As new variants continuously emerge, predicting critical clinical events in the context of relevant individual risks is a promising option for reducing the overall burden of COVID-19. The aim of this study was threefold: (1) present patient characteristics and comorbidities among 5,371 patients hospitalized with COVID-19 in Southern Florida, (2) develop an AI-driven decision support system that helps build a model to understand the most important features that predict ‘mortality’ in patients hospitalized with COVID-19; and (3) set a platform for future work to forecast treatment plans for such critical clinical events that slow severity of COVID-19 disease, optimize individual outcomes, find quick treatment modules, and decrease the economic burden.

**Methods:** We conducted a retrospective analysis of 5,371 COVID-19 disease patients hospitalized for COVID-19-related symptoms from South Florida Memorial Health Systems between March 14^th^, 2020, and January 16^th^, 2021. Demographics, patient characteristics, and pre-existing health data in the dataset were collected at admission. We trained Random Forest Classifier to predict ‘mortality’ for hospitalized patients who were infected with the SARS-CoV-2 virus. Our respective Institutional Review Board (IRB) approved the study with the exemption of informed consent and HIPAA waiver. IRB also determined that this project is exempt from further review.

**Results:** According to the model interpretability (SHAP), the main predictor of the outcome was age, followed by BMI, diarrhea, hypertension, early stages of kidney disease, diabetes, race, pneumonia, smoking status, and gender in ranking order. Significant differences were found among the mean of the variables mentioned earlier between the two patient groups: (1) expired; (2) survived. It was also noted that individuals over 65 (‘older adults’), including ‘males’, ‘whites’, ‘Alaska Native Americans, and ‘current smokers’, were at greater risk of death. On the other hand, BMI, classified as ‘overweight’ and ‘obesity’, was a significant indicator of mortality. The study also reported that regular use of medicines (‘ARBs’ & ‘ACEs’) to treat high blood and heart failure could reduce mortality. These findings suggested that the model could learn features from each category, such as ‘patients’ characteristics, ‘pre-hospital comorbidities, and ‘medications’ but mostly from characterizing ‘pre-hospital comorbidities.’ Therefore, the model revealed the potential to be effective in measuring ‘mortality’ while being transparent and reliable. The small scores corresponded to small increases in Root Mean Square Error (RMSE) — evidence of better model performance in both seen (train) and unseen (test) cases in the current study.

**Conclusion:** The performance of the study model is consistent with other Machine Learning (ML) tools^1,2^ used in various health domains. AI can potentially provide healthcare workers with the ability to stratify patients and streamline optimal care solutions when time is of the essence and resources are limited. This work sets the platform for future work that forecasts patient responses to treatments at various levels of disease severity, identifies patients at high risk of developing long-term complications, and assesses health disparities and patient conditions that promote improved health care in a broader context.

**References**

1. Shorten, C., Cardenas, E., Khoshgoftaar, T. M., Hashemi, J., Dalmida, S. G., Newman, D., ... & Eckard, P. (2022, October). Exploring Language-Interfaced Fine-Tuning for COVID-19 Patient Survival Classification. In 2022 IEEE 34th International Conference on Tools with Artificial Intelligence (ICTAI) (pp. 1449-1454). IEEE.
2. Shorten, C., Khoshgoftaar, T. M., Hashemi, J., Dalmida, S. G., Newman, D., Datta, D., ... & Eckard, P. (2022, May). Predicting the Severity of COVID-19 Respiratory Illness with Deep Learning. In *The International FLAIRS Conference Proceedings* (Vol. 35).

## 06 Identifying associations between hotspots of social determinants of health needs, community-based organizations and healthcare resource use

### Reid DeMass^1^, Stella Self^1^, Caroline Rudisill^2^, Deeksha Gupta^2^, Anna Chupak^2^, Darin Thomas^3^

#### ^1^Department of Epidemiology and Biostatistics, Arnold School of Public Health, University of South Carolina, Columbia, SC, USA; ^2^Department of Health Promotion, Education, and Behavior, Arnold School of Public Health, University of South Carolina, Columbia, SC, USA; ^3^Addiction Medicine Center, Prisma Health, Greenville, SC, USA

##### **Correspondence:** Reid DeMass (rdemass@email.sc.edu)

*BMC Proceedings 2023*, **17(Suppl 19):**O6

**Study Objectives**: The project aimed to identify block group locations in South Carolina exhibiting high levels (hotspots) of social determinants of health (SDoH) needs, community-based organizations (CBOs) and healthcare resource use. The study hypothesized that areas with SDoH hotspots would be associated with resource use hotspots, defined by emergency department (ED), primary care physician visits (PCP), and in-patient (IP) hospital care. Furthermore, the study investigated whether there are overlaps in hotspots for a given SDoH need and CBOs designed to aid that need.

**Methods**: The study sample included Prisma Health patients, aged 18+ years, engaged in ambulatory care and condition management, in-patient case management, or community health in South Carolina’s central and northwestern regions. Data was collected for June 1, 2019-December 31, 2020. Information on patients’ SDoH needs and their respective CBOs were taken from the NowPow referral system. The five SDoH needs categories included food insecurity, housing instability/quality, lack of transportation, financial instability, and two indicators of social connectedness. EMR supplied patients’ ED, PCP, and IP data. Both patient and CBO addresses were geocoded and each was linked to a U.S. Census block group.

The geographic area of interest was limited to block groups within ten neighboring counties in South Carolina. For each block group, the following variables were calculated: number of patients with a given SDoH need (five variables); number of CBOs servicing each SDoH need (five variables); and the mean number of ED, IP, and PCP visits (three variables). The Getis-ord G_i_^*^ statistic was used to identify block groups that were hotspots for each of these variables (food insecurity example, Figure 1). First, chi-square tests detected whether there was an association between hotspots for a given SDoH need and hotspots for the respective CBOs. Then, chi-square tests were made between all SDoH needs hotspots to each of the health resource use hotspots.

**Results**: For tests between SDoH needs and respective CBOs, significant associations were found for food insecurity and food-based CBOs (p-value=0.035) as well as social isolation and its respective CBOs (p-value=0.048, Table 1). Hotspots for IP visits were associated with SDoH hotspots for financial instability and both measures of social connectedness; however, the associations were not significant after adjusting for multiple comparisons. No significant associations were found between other categories of healthcare resource use (ED, PCP) and SDoH hotspot status.

**Discussion**: Identifying hotspot associations can generate hypotheses about the relationship between SDoH needs, the CBOs working to mitigate such needs, and the impact these community features have on healthcare resource use needs. For example, if there is a lack of CBOs for financial instability, then that might contribute to increased IP visits. Moreover, hotspots of food insecurity overlapping with food-oriented CBOs might indicate that CBOs are situated in the appropriate communities. More research will assist in ascertaining the underlying mechanisms behind the identified associations, and lack thereof. The current study serves as a starting point for further analysis in identifying the relationships between SDoH and CBO hotspots and how community features may impact healthcare service use.

**Fig. 1 (abstract O6).**
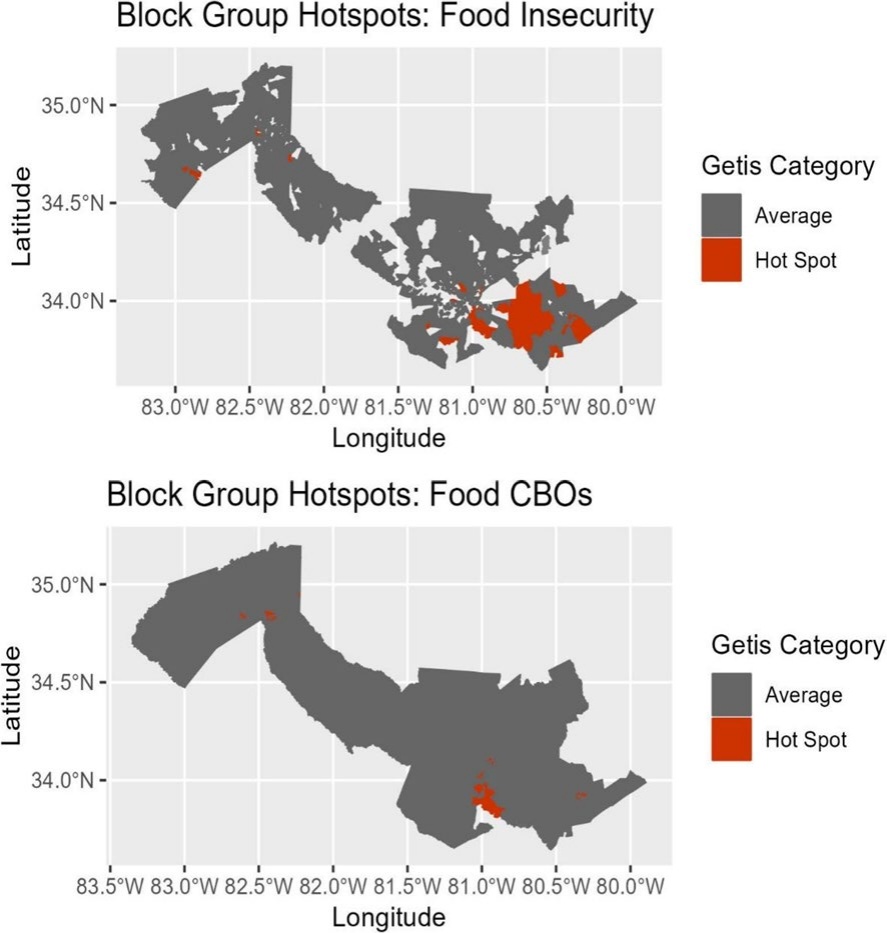
Comparison of food insecurity hotspots and respective CBO hotspots

**Table 1 (abstract O6).** SDoH needs and respective CBOs hotspot overlap

Variable	p-value
Food insecurity	0.035*
Housing instability	0.651
Transportation need	1.00
Financial instability	0.122
Social connectedness (L)	1.00
Social connectedness (SI)	0.048*

*p-value <0.05

## O7 Who is your prenatal care provider? An algorithm to identify the predominant prenatal care provider with claims data

### Songyuan Deng, Samantha Renaud, Kevin J Bennett^1^

#### South Carolina Center for Rural and Primary Healthcare, School of Medicine Columbia, University of South Carolina, Columbia, SC, USA

##### **Correspondence:** Kevin Bennett (Kevin.Bennett@uscmed.sc.edu)

*BMC Proceedings 2023*, **17(Suppl 19):**O7

Declaration: Exemption for this study was obtained from the Institutional Review Board at the University of South Carolina due to the secondary analysis using de-identifiable administrative data.

**Objective:** Many women visit multiple providers over the course of their pregnancy, making identifying the predominant provider difficult. Using claims data to identify a predominant prenatal care (PNC) provider is not always straightforward, yet it is essential for assessing access ^1^, cost ^2^, and outcomes ^3^. Previous algorithms applied plurality ^4^ (providing the most visits) and majority ^5^ (providing at least half of all visits) criteria to identify the predominant provider in the primary care setting, but they lacked visit sequence information. Except visit frequency, PNC initiation is crucial for PNC quality indices, and the last PNC may involve delivery referral. This study proposes an algorithm that includes PNC sequence information to identify the predominant provider and estimates the percentage of pregnancies with an identifiable predominant PNC provider. Additionally, differences in travel distances to the predominant and nearest provider are compared.

**Method:** The dataset used for this study consisted of 108,441 live births and 2,155,076 associated claims from the 2015-2018 South Carolina Medicaid, obtained from the South Carolina Revenue and Fiscal Affairs (RFA) Office. The analysis focused on patients who were continuously enrolled in Medicaid throughout their pregnancy and had at least one PNC visit, resulting in 32,609 pregnancies. PNC visits were identified by comparing delivery date with claim date and refined using claim diagnosis and procedure codes as well as specialty.^6^

To classify PNC providers, seven subgroups were created based on PNC frequency and sequence information (Table 1). A stepwise algorithm was developed to determine the predominant PNC provider, considering both the frequency of PNC visits (in all scenarios) and the sequence of visits (in scenarios 4, 5, and 6) (Table 1). PNC dispersion information was used as supplementary data, as it is impossible to identify a predominant provider if the number of visits equals the number of providers. The percentage of identified predominant providers was reported.

*Chi*-square tests were conducted to assess whether the probability of being identified as a predominant provider for a specific subgroup differed from that of the reference group (PNC(M) - providing at least half of all PNC). Paired *t*-tests were used to examine differences in travel distance.

**Results:** By applying PNC frequency information, a predominant PNC provider can be identified for 81% of pregnancies. If PNC sequential information is also included, a predominant PNC provider can be identified for 92% of pregnancies. (Table 1) The predominant provider was identified mostly as the order of PNC(U), PNC(E), PNC(M), PNC(MFVI) and PNC(MFVF). (Table 1) Distance was significantly shorter for pregnant women traveling to the nearest visited PNC provider (an average of 5 miles) than to the identified predominant PNC provider.

**Discussion:** This algorithm provides researchers and policymakers flexibility in identifying the predominant PNC providers. The inclusion of PNC sequential information in the algorithm has increased the proportion of identifiable predominant providers from 81% to 92%, an increase of 11%. Applying this algorithm reveals a longer distance for pregnant women travelling to their predominant PNC provider than to the nearest PNC provider.

**References**

1. Blewett LA, Johnson PJ, Lee B, Scal PB. When a usual source of care and usual provider matter: Adult prevention and screening services. *J Gen Intern Med*. 2008;23(9). doi:10.1007/s11606-008-0659-0
2. Nicolet A, Al-Gobari M, Perraudin C, Wagner J, Peytremann-Bridevaux I, Marti J. Association between continuity of care (COC), healthcare use and costs: what can we learn from claims data? A rapid review. *BMC Health Serv Res*. 2022;22(1):1-30.
3. Gray DJP, Sidaway-Lee K, White E, Thorne A, Evans PH. Continuity of care with doctors - A matter of life and death? A systematic review of continuity of care and mortality. *BMJ Open*. 2018;8(6). doi:10.1136/bmjopen-2017-021161
4. Weiner JP, Parente ST, Garnick DW, Fowles J, Lawthers AG, Palmer RH. Variation in Office-Based Quality: A Claims-Based Profile of Care Provided to Medicare Patients With Diabetes. *JAMA: The Journal of the American Medical Association*. 1995;273(19). doi:10.1001/jama.1995.03520430039036
5. Rosenblatt RA, Hart LG, Baldwin LM, Chan L, Schneeweiss R. The generalist role of specialty physicians: Is there a hidden system of primary care? *JAMA*. 1998;279(17). doi:10.1001/jama.279.17.1364
6. Sarayani A, Wang X, Thai TN, Albogami Y, Jeon N, Winterstein AG. Impact of the transition from icd–9–cm to icd–10–cm on the identification of pregnancy episodes in us health insurance claims data. *Clin Epidemiol*. 2020;12. doi:10.2147/CLEP.S269400

**Table 1 (abstract O7).** The percentage of pregnancies with identifiable predominant prenatal care providers, by prenatal care frequency

Prenatal care frequency	All providers	Frequency			Frequency + sequence			Dispersion
		PNC(E)	PNC (M) (ref)	PNC (U)	PNC (MFVI)	PNC (MFVF)	PNC (MFVIF)	Dispersal PNC
All frequency	100.00	28.40	19.55	33.49	7.31	3.05	1.42	8.19
PNC =1	6.18	6.18	0.00	0.00	0.00	0.00	0.00	0.00
8 ≥ PNC >1	48.99	12.24 ^***^	9.01	14.98	4.08 ^***^	1.28 ^*^	0.70	7.40 ^***^
14 ≥ PNC >8	40.22	8.78 ^***^	9.52	16.52	3.03 ^***^	1.66 ^*^	0.67	0.70 ^***^
PNC >14	4.62	1.20 ^**^	1.02	1.99 ^***^	0.20	0.12 ^**^	0.05	0.09 ^***^
Sum (Scenarios)								
1, PNC(E)		28.40						
2, 1+PNC(M)		47.95						
3, 2+PNC(U)		81.44						
4, 3+PNC(MFVI)		88.75						
5, 3+PNC(MFVF)		88.40						
6, 4+ PNC(MFVF)		91.81						

Seven PNC subgroups: PNC(E) – The exclusive PNC provider for one pregnancy. PNC(M) – this provider served more than half of all visits for one pregnancy. PNC(U) – the uniquely most frequently visited provider, who is the only one who served the most visits for a patient. PNC(MFVI) - most frequently visited provider, who served the most visits and the first visit for a patient. PNC(MFVF) - most frequently visited provider, who served the most visits and the last visit for a patient. PNC(MFVIF) - most frequently visited provider, who served the most visits, first and the last visits for a patient. Dispersal PNC: the number of total PNC visits equals the number of total providers

*: p < .05, **: p < .01, ***: p < .001

## O8 Epitweetr: development and implementation of a tool with AI components to monitor Twitter trends for early warning of threats to public health

### Laura Espinosa (laura.espinosa@ecdc.europa.eu)

#### European Centre for Disease Prevention and Control, Solna, Sweden

*BMC Proceedings 2023*, **17(Suppl 19):**O8

**Abstract Narrative:** Following the spiral process of social innovation, the European Centre for Disease Prevention and Control developed the open-source R-based tool epitweetr for early detection of threats to public health using Twitter. The methodology used in this project allows for a continuous improvement and increased usability of epitweetr by public health experts.

**Issues:** The European Centre for Disease Prevention and Control (ECDC) aims at strengthening Europe’s defences against infectious diseases. Epidemic intelligence (EI), one of the core functions of the organization, is the process of collating, filtering, validating and analysing information from a wide variety of sources, including social media, for the early detection of threats to public health.

Previous studies have shown the usefulness of social media platforms for public health surveillance and real-time monitoring of outbreaks.

The objective of this project was to improve the timeliness and effectiveness of a Twitter-based threat detection tool.

**Project:** The project was developed following the spiral process of social innovation.

The initial inspiration was the lack of a configurable tool for monitoring Twitter trends for early detection of public health threats. Twitter was chosen due to the publicly available data, well documented application programming interface (API) and R packages facilitating the collection of Twitter data.

Between July 2019 and February 2020, based on a list of requirements, an R-based prototype was developed as a proof-of-concept study. It included processes for data collection, aggregation and visualisation, and email notifications for three diseases. The prototype did not include a signal detection algorithm but this was identified as a key functionality for development.

Following this successful prototype, in 2020, ECDC outsourced the development of an R package and Shiny app which would allow for automated signal detection. This was done through a multidisciplinary team of public health experts, epidemiologists, statisticians and data scientists. An expert workshop was organised in June 2020 to discuss proposals and ideas for the final version, and we performed beta testing with four potential users. The first version published in October 2020 included a signal detection algorithm based on a modified early aberration reporting system, as well as a machine learning model for extracting the geolocation of from the user’s profile and from the content of the tweet.

The use of epitweetr has rapidly increased. It is part of the daily ECDC epidemic intelligence activities. The ECDC EI team and the ECDC 24/7 duty officers have been trained on its use and maintenance. Epitweetr has been presented in different fora and online trainings have been organised for public health experts. Developing epitweetr as a free open-source R package enhanced its use outside of ECDC.

Between September and November 2020, we assessed epitweetr’s performance in comparison with manual monitoring of Twitter and the performance of its signal detection algorithm and machine learning model for tweet geolocation.

Two additional versions of epitweetr have been developed in 2021 and 2022, following the results of this evaluation and its use by different stakeholders, including ECDC (figure 1).

**Lessons Learned:** This project has shown the importance of following the steps in the innovation spiral, from developing a prototype through scaling and finally systematic change, to achieve a successful output. Likewise, having a multidisciplinary team behind its development and making epitweetr as an open-source tool, allowed for continuous improvement and increased usability of epitweetr in the public health community.

**References**

1. Regulation (EU) 2022/2370 of the European Parliament and of the Council of 23 November 2022 amending Regulation (EC) No 851/2004 establishing a European centre for disease prevention and control. http://data.europa.eu/eli/reg/2022/2370/oj
2. Murray R, Caulier-Grice J, Mulgan G. *The open book of social innovation.* The Young Foundation; 2010. https://youngfoundation.org/wp-content/uploads/2012/10/The-Open-Book-of-Social-Innovationg.pdf
3. Nesta. *A compendium of innovation methods.* https://www.peterfisk.com/wp-content/uploads/2019/10/Compendium-of-Innovation-Methods.pdf
4. Li C, Chen LJ, Chen X, Zhang M, Pang CP, Chen H. Retrospective analysis of the possibility of predicting the COVID-19 outbreak from Internet searches and social media data, China, 2020. *Euro Surveill*. 2020;25(10). 10.2807/1560-7917.ES.2020.25.10.2000199
5. Jordan S, Hovet S, Fung I, Liang H, Fu K-W, Tse Z. Using Twitter for Public Health Surveillance from Monitoring and Prediction to Public Response. *Data* (Basel). 2018;4(1):6. 10.3390/data4010006
6. Van de Belt TH, van Stockum PT, Engelen LJLPG, Lancee J, Schrijver R, Rodríguez-Baño J, et al. Social media posts and online search behaviour as early-warning system for MRSA outbreaks. *Antimicrob Resist Infect Control*. 2018;7(1):69. 10.1186/s13756-018-0359-4
7. Tsao S-F, Chen H, Tisseverasinghe T, Yang Y, Li L, Butt ZA. What social media told us in the time of COVID-19: a scoping review. *Lancet Digit Health*. 2021;3(3):e175-94. 10.1016/S2589-7500(20)30315-0
8. Espinosa L, Wijermans A, Orchard F, Höhle M, Czernichow T, Coletti P, Hermans L, Faes C, Kissling E, Mollet T. Epitweetr: Early warning of public health threats using Twitter data. *Euro Surveill*. 2022;27(39):pii=2200177. 10.2807/1560-7917.ES.2022.27.39.2200177
9. Espinosa L. Welcome to epitweetr!. Epitweetr. Updated April 20, 2023. Accessed June 6, 2023. https://github.com/EU-ECDC/epitweetr/discussions/7

**Fig. 1 (abstract O8).**
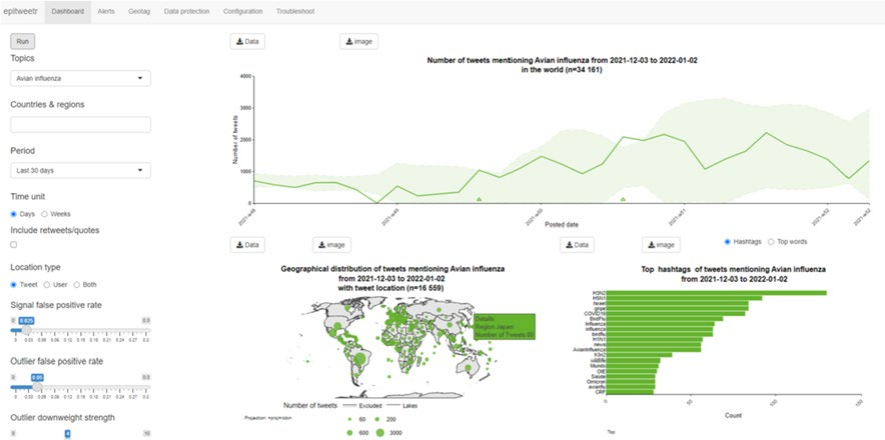
Shiny app of epitweetr version 2.2.13 published on November 30, 2022

## O9 Using label-free two-photon microscopy and deep learning image processing to assess the chondrocyte viability of articular cartilage

### Hongming Fan^1^, Pei Xu^2^, Zhao Zhang^1^, Bruce Gao^1^, Tong Ye^1,3^

#### ^1^Department of Bioengineering, Clemson University, Clemson, SC, USA; ^2^School of Computing, Clemson University, Clemson, SC, USA; ^3^Department of Regenerative Medicine and Cell Biology, Medical University of South Carolina, Charleston, SC, USA

##### **Correspondence:** Hongming Fan (hongmif@g.clemson.edu)

*BMC Proceedings 2023*, **17(Suppl 19):**O9

**Abstract**

Articular cartilage has a limited healing capacity. Traumatic and degenerative lesions eventually progress to osteoarthritis (OA), a leading source of disability worldwide. Among various surgical resurfacing treatments, osteochondral allograft transplantation has been proven with good clinical outcomes, especially for relatively large lesions. Osteochondral allografts have become increasingly popular due to the improvement and standardization in allograft storage. As the chondrocyte is the only cell type in articular cartilage, chondrocyte viability (CV) at the time of implantation is believed to be a critical factor in ensuring long-term allograft survival in vivo. However, viability assessment usually requires dye labeling; tissues are not unusable after the evaluation. Autofluorescence of intracellular fluorescent coenzymes, such as reduced forms of nicotinamide adenine dinucleotide (NADH) or nicotinamide adenine dinucleotide phosphate (NADPH) and oxidized flavoproteins (FPs), have been long used as a label-free means to study metabolic states of cells^1,2^. We previously demonstrated CV could be assessed by a non-labeling method^3^ using two-photon excitation autofluorescence (TPAF) and second harmonic generation (SHG) imaging on rat and porcine cartilage samples, where TPAF/SHG images were merged to form RGB color images with red, green, and blue assigned to FPs, NAD(P)H, and collagen signal channels, respectively. We have also developed deep learning based segmentation and classification algorithms for CV measurement^4^. In this presentation, we introduce a new network for CV measurement using the Mask R- CNN architecture^5^, a robust instant segmentation algorithm just became available in recent years. The new CV network has two significant improvements over the previously proposed method^4^. Firstly, the previous method uses separate networks for segmentation and classification; each network needs its own training and test data sets, which increases the workload of both annotation and training. The new network is a single integrated architecture that can identify individual cells and, at the same time, classify them with live or dead status. This integrated architecture only needs a single annotated training and test dataset, making the learning and viability analysis more efficient. Secondly, the new network performs instant segmentation, which identifies each chondrocyte as a distinct object with the category that it belongs to and the boundary that separates it from the rest of the pixels in an image. Instant segmentation improves the accuracy of both segmentation and classification. Moreover, we have found that Wiener deconvolution preprocessing can significantly improve the accuracy of CV measurement. Taken together, the accuracy of new automated CV measurement has achieved over 95% on both rat and porcine knee cartilage samples. We believe the two-photon microscopy plus the current version of the CV measurement network offers a reliable, label-free method for assessing CV of allografts.

**References**

1. Chance B. Optical method. *Annu Rev Biophys Biophys Chem*. 1991;20:1-28. doi:10.1146/annurev.bb.20.060191.000245
2. Georgakoudi I, Quinn KP. Optical imaging using endogenous contrast to assess metabolic state. *Annu Rev Biomed Eng*. 2012;14:351-367. doi:10.1146/annurev-bioeng-071811-150108
3. Li Y, Chen X, Watkins B, et al. Nonlabeling and quantitative assessment of chondrocyte viability in articular cartilage with intrinsic nonlinear optical signatures. *Exp Biol Med Maywood NJ*. 2020;245(4):348-359. doi:10.1177/1535370219896545
4. Chen X, Li Y, Wyman N, et al. Deep learning provides high accuracy in automated chondrocyte viability assessment in articular cartilage using nonlinear optical microscopy. *Biomed Opt Express*. 2021;12(5):2759-2772. doi:10.1364/BOE.417478
5. He K, Gkioxari G, Dollár P, Girshick R. Mask R-CNN. Published online January 24, 2018. doi:10.48550/arXiv.1703.06870

## O10 Predicting surgical outcome in epilepsy with diffusion MRI

### Dai Fang^1^, Carrie McDonald^2^, Leonardo Bonilha^3^, Yuan Wang^1^

#### ^1^Department of Epidemiology and Biostatistics, Arnold School of Public Health, University of South Carolina, Columbia, SC, USA; ^2^Department of Psychiatry, University of California, San Diego, CA, USA; ^3^Department of Neurology, Emory University, Atlanta, GA, USA

##### **Correspondence:** Yuan Wang (wang578@mailbox.sc.edu)

*BMC Proceedings 2023*, **17(Suppl 19):**O10

**Abstract Narrative:** Epilepsy is a severe neurological disorder affecting over 50 million people worldwide. Leveraging the largest international neuroimaging database on epilepsy provided by the Enhancing Neuroimaging Genetics through Meta-analysis (ENIGMA) Consortium, we apply gold-standard machine learning models to predict surgical outcome in epilepsy patients with diffusion MRI measures.

**Study Objective:** Epilepsy is a severe neurological disorder affecting over 50 million people worldwide. Approximately one third of epilepsy patients are resistant to anti-epileptic drug treatment and require additional diagnostic procedures such as electroencephalographic (EEG) evaluation to localize the neuronal network capable of generating seizures, for surgical resection. This approach, however, relies heavily on the expertise of the specialist clinicians reading the EEG. Non-invasive neuroimaging techniques such as magnetic resonance imaging (MRI) thus play a critical role in the diagnosis of patients with focal epilepsy through identifying visible lesions. Yet, currently around 20 – 40% of focal epilepsy patients do not show lesions on MRI, let alone generalized epilepsy cases that are by default non-lesional. A computational modeling approach to MRI has shown promise in providing new insight on brain structure and function, as well as treatment response, related to epilepsy, but past studies tend to be limited in sample sizes and power. Leveraging the first and currently the largest international neuroimaging database on epilepsy provided by the Enhancing Neuroimaging Genetics through Meta-analysis (ENIGMA) Consortium and its Global Alliance for Worldwide Imaging in Epilepsy (ENIGMA-Epilepsy), we can now integrate neuroimaging data of patients from 24 epilepsy centers in 14 countries to answer complex clinical questions in epilepsy. The objective of this pilot study was to compare performance of gold-standard machine learning models in predicting surgical outcome in epilepsy patients with diffusion MRI (dMRI) measures.

**Method:** Four dMRI measures (AD, RD, MD, FA) from 270 patients with temporal lobe epilepsy (TLE) undergoing surgery were included in the study. To eliminate site effect, the dMRI measures from all epilepsy centers were standardized by clinical variables using the COMBAT harmonization algorithm. The surgical outcome of epilepsy patients was measured by Engel class (I through IV representing seizure freedom through no improvement). Patients with Engel class I&II were merged into the Favorable Outcome group, while patients with Engel class III&IV were merged into the Unfavorable Outcome group. A uniform resampling strategy was applied to balance out the sample sizes across the two surgical outcome groups. The Engel group labels of patients were classified with deep neural network (DNN) with respect to respective correlation matrix constructed from the AD, RD, MD and FA measures from 38 regions of interest (ROIs) per the JHU brain atlas. Performance measures included means and standard deviations of accuracy, sensitivity, specificity, PPV, and NPV calculated after 30, 40, and 50 iterations of resampling from the patients in the two surgical outcome groups.

**Results:** DNN based on AD had the best performance compared to RD, MD and FA. For 30 iterations of resampling using AD, the mean accuracy, sensitivity, specificity, PPV and NPV are 94.2%, 95.2%, 93.2%,93.93%, and 95.15% respectively; for 40 iterations, the mean accuracy, sensitivity, specificity, PPV and NPV are 96.57%, 96.57%, 96.57%, 96.77%, and 96.75% respectively; for 50 iterations, the mean accuracy, sensitivity, specificity, PPV and NPV using AD are 97.35%, 98.24%, 96.47%, 96.74%, and 98.29% respectively.

**Discussion:** The results obtained through gold-standard DNN provide a baseline for more advanced learning approaches in predicting surgical outcome with dMRI measures.

## O11 Application of machine learning in predicting breast cancer patient outcome

### Ali Firooz^1^, Savannah M. Noblitt^1^, Julie Martin^2^, W. Jeffery Edenfield^2^, Anna Blenda^2,3^, Homayoun Valafar^1^

#### ^1^Department of Computer Science and Engineering, College of Engineering and Computing, University of South Carolina, Columbia, SC, USA; ^2^Prisma Health Cancer Institute, Greenville, SC, USA; ^3^School of Medicine Greenville, University of South Carolina, Greenville, SC, USA

##### **Correspondence:** Homayoun Valafar (homayoun@cse.sc.edu)

*BMC Proceedings 2023*, **17(Suppl 19):**O11

**Study Objectives:** Breast cancer is a significant health concern in the United States, ranking as the second leading cause of death. The complex nature of cancer, with its diverse subtypes and heterogeneity, makes accurate diagnosis and treatment challenging. Current medical practices often fail to integrate molecular diagnostics with clinical data, hindering the identification of early disease markers and prediction of patient responses to targeted therapies. The multifactorial nature of cancer, influenced by patient health, co-morbidities, environment, and molecular factors, requires the compilation and presentation of data in an accessible manner for tailored treatments. Unifying and standardizing data is a critical step in utilizing machine learning tools to unravel complex relationships in cancer and enable personalized care^1^. The primary objective of this work is to develop a comprehensive and user-friendly repository of cancer patient data.

**Methods:** A Relational Database Management System has been designed and has been populated by the first round of clinical data from the Prisma Health Cancer Institute Biorepository of ~6,000 cancer patients with at least 66 different cancer diagnoses^2^. Molecular data is available for gene mutations, serum galectin proteins, and glycomic profiles of cancer patients. Mutation status of 50 cancer-critical genes in 1,500 patients, 320 individual patient profiles of 5 serum galectin proteins, and serum and biopsy glycomic profiles of 60 patients have been included and will be expanded. In addition, healthy control values for galectin and glycomic profiles were obtained and added for reference. To further address the issue of easy access to the data, a user-friendly and intuitive web application interface is being developed using Flask web framework.

**Results:** The use of typical statistical analyses (linear and logistic regression) has revealed insignificant correlation between the predictors and the cancer type of patient outcome. However, the use of the Decision Trees revealed some interesting relationships that can be used for explainability and reliability of the Machine Learning approaches. Based on the initial explorations, Decision Trees have demonstrated a performance approaching 80% success in determining the treatment outcomes.

**Discussion:** Our studies provide predictive models that could potentially be used to improve the diagnostic and prognostic power of data collected from patients at presentation. However, the dichotomy of black box AI approaches perform better than explainable approaches, complicating deployment of these techniques in the domain of medicine and healthcare.

**References**

1. Odigwe, B. E., Rajeoni, A. B., Odigwe, C. I., Spinale, F. G., & Valafar, H. (2022). Application of machine learning for patient response prediction to cardiac resynchronization therapy. Proceedings of the 13th ACM International Conference on Bioinformatics, Computational Biology and Health Informatics, 1–4. 10.1145/3535508.3545513
2. Firooz, A., Funkhouser, A. T., Martin, J. C., Edenfield, W. J., Valafar, H., & Blenda, A. V. (2023). Comprehensive and user-analytics-friendly cancer patient database for physicians and researchers. 10.48550/ARXIV.2302.01337

## O12 Disparities in mental health service utilization among immigrants in the U.S. using geospatial big data

### Fengrui Jing^1,2^, Zhenlong Li^1,2^, Shan Qiao^2,3^, Huan Ning^1,2^, Jiajia Zhang^2,4^, Bankole Olatosi^2,5^, Xiaoming Li^2,3^

#### ^1^Geoinformation and Big Data Research Lab, Department of Geography, University of South Carolina, Columbia, SC, USA; ^2^Big Data Health Science Center, University of South Carolina, Columbia, SC, USA; ^3^Department of Health Promotion, Education, and Behavior, Arnold School of Public Health, University of South Carolina, Columbia, SC, USA; ^4^Department of Epidemiology and Biostatistics, Arnold School of Public Health, University of South Carolina, Columbia, SC, USA; ^5^Department of Health Services Policy and Management, Arnold School of Public Health, University of South Carolina, Columbia, SC, USA

##### **Correspondence:** Fengrui Jing (fengrui@sc.edu)

*BMC Proceedings 2023*, **17(Suppl 19):**O12

**Abstract**

Immigrants (foreign-born individuals) are an essential part of United States (US) society, accounting for more than 10 percent of the population. Immigrants utilize mental health services differently than the US-born, and previous studies did not fully explain the differences using individual-level data. Leveraging mobile phone-based mental health service utilization (MHSU) data, we estimated the average mental health visit in contiguous US census tracts from 2019 (pre-COVID-19-outbreak) to 2021 (after-COVID-19-outbreak). Two outcomes were measured: average MHSU and MHSU per new depression diagnosis (MHSU-to-need ratio [MnR]). We then investigated the tract-level association between immigrant population proportions and MHSU indicators using mixed-effects linear regression models that accounted for spatial lag effects, time effects, propensity, enabling, and need factors. Neighborhoods with higher immigration percentages had lower MHSU but higher MnR. In detail, neighborhoods dominated by Latino immigration were significantly associated with lower MHSU and MnR, particularly in the U.S. West and South. When the immigrant's country of origin is in Asia, a higher immigrant percentage was associated with a lower MHSU but a higher MnR. These relationships also vary over time. All neighborhoods experienced significant decreases in MnR and MHSU in 2020, but immigrant neighborhoods recover the slowest in 2021 when compared to their counterparts. Our findings demonstrate disparities in MHSU by immigrant status, and these disparities are spatiotemporally heterogeneous. Immigrant-dominated neighborhoods with disproportionately low mental health visits can be monitored in real-time using emerging mobile phone-based MHSU data to precisely provide additional resources to address MHSU disparities.

## O13 Using Semantic Web Technology to leverage interoperable clinical decision support system rules: a pathway to interoperable patient records

### Xia Jing^1^, James J. Cimino^2^, Dean F. Sittig^3^, Hua Min^4^, Yang Gong^3^, Richard D. Boyce^5^, David Robinson^6^, Paul Biondich^7^, Adam Wright^8^, Christian G. Nøhr^9^, Timothy Law^10^, Arild Faxvaag^11^, Nina Hubig^12^, Rohan Goli^12^, Karthik Nedunchezhiyan^12^, Akash Shanmugam Boobalan^12^, Samuil Orlioglu^12^, Chloe Crozier^12^, Lior Rennert^1^, Ronald Gimbel^1^

#### ^1^Department of Public Health Sciences, College of Behavioral, Social and Health Sciences, Clemson University, Clemson, SC, USA; ^2^Informatics Institute, The University of Alabama at Birmingham, Birmingham, AL, USA; ^3^School of Biomedical Informatics, The University of Texas Health Sciences Center at Houston, Houston, TX, USA; ^4^Department of Health Administration and Policy, College of Public Health, George Mason University, Fairfax, VA, USA; ^5^Department of Biomedical Informatics, University of Pittsburg School of Medicine, Pittsburgh, PA, USA; ^6^Independent Consultant, Cumbria, United Kingdom; ^7^Department of Pediatrics, Clem McDonald Biomedical Informatics Center, Regenstrief Institute, Indiana University School of Medicine, Indianapolis, IN, USA; ^8^Department of Biomedical Informatics, Vanderbilt University Medical Center, Nashville, TN, USA; ^9^Department of Planning, Faculty of Engineering, Aalborg University, Aalborg, Denmark; ^10^Ohio Musculoskeletal and Neurologic Institute, Ohio University, Athens, OH, USA; ^11^Department of Neuromedicine and Movement Science, Faculty of Medicine and Health Sciences, Norwegian University of Science and Technology, Trondheim, Norway; ^12^School of Computing, College of Engineering, Computing and Applied Sciences, Clemson University, Clemson, SC, USA

##### **Correspondence:** Xia Jing (xjing@clemson.edu)

*BMC Proceedings 2023*, **17(Suppl 19):**O13

**Issues**: Interoperability is a well-recognized significant barrier to sharing patients’ medical records seamlessly^1-3^. Although HL7, the set of international standards for communicating clinical and administrative data, plays a critical role in achieving interoperability in healthcare, not all institutions can afford to be compatible with these standards. Having interoperable clinical decision support systems (CDSS) rules can provide a significant step toward realizing interoperable patient records, which are much more complicated than just CDSS rules.

**Project**: We propose using CDSS ontology, an enabling technology of the Semantic Web, to leverage interoperable CDSS rules, which ultimately determine the behaviors of CDSS. We use resource-constrained primary care settings as the ideal targets and two existing open-source electronic health records systems (OpenMRS^4^ and OpenEMR^5^) to demonstrate the feasibility. Figure 1 shows the conceptual model of the project.

To construct the CDSS ontology^6^, we used manual and automatic approaches that complement each other^7,8^. The manual approach primarily involved expert input and iterative feedback. The automatic approach included natural language processing techniques and neural network architecture with semi-supervised learning and transfer learning to identify entity candidates for CDSS ontology from publications about CDSS. Figure 2 shows the primary approach to constructing the CDSS ontology. Centers for Disease Control (CDC) vaccination schedules (0–18 years) are used to generate machine-readable CDSS rules using unambiguous concepts and relationships provided by CDSS ontology. We are in the process of translating CDC tabular vaccination schedules into CDSS rules that programmers can use. The ontology construction is underway, and the automatic part has made excellent progress.

We use OpenMRS and OpenEMR as testbeds for the CDSS rules. We create a CDSS module for OpenMRS and an enhanced CDSS module for the current version of OpenEMR, which does not currently have management and maintenance functionalities. We aim to make the modules easier to be reused and shared. Meanwhile, FAIR (findability, accessibility, interoperability, and reusability) principles will also be followed^9^. We are comparing different solutions and specifying detailed requirements for the CDSS modules and the tracking mechanisms.

The project will create the following artifacts for sharing among the community: a CDSS ontology, machine-readable CDSS rules on the CDC vaccination schedule (0–18 years), a CDSS module for OpenMRS, an enhanced CDSS module for OpenEMR, and an open course on CDSS regarding its design, development, use, maintenance, and evaluation. The course will be organized in the formats of lectures and hands-on sessions. The project is ongoing, and its progress will be shared.

**Lessons learned and their implications**: Community engagement is a critical and challenging component in ontology development, evaluation, and adoption, as well as in the lifecycle of machine-readable CDSS rules^10^. Moreover, balancing the empowerment of end users by providing flexibility and configuration capabilities and making the module easy to use is also essential and challenging. An example from this case study is providing a complete set of CDSS rules for management and maintenance while ensuring the end users are not overwhelmed. The revised CDSS rules can be validated and verified.

**Acknowledgment**

This work was supported by the National Institute of General Medical Sciences of the National Institutes of Health under Award No. R01GM138589, 3R01GM138589-03S1, and partially under P20 GM121342.

**References**

1. Blobel B. Knowledge representation and management enabling intelligent interoperability - principles and standards. *Stud Health Technol Inform*. 2013;186:3-21.
2. Greenes R. *Clinical decision support: the road to broad adoption*. 2nd ed. Elsevier; 2014.
3. Jing X, Min H, Gong Y, et al. Ontologies Applied in Clinical Decision Support System Rules: Systematic Review. Review. *JMIR medical informatics*. 2023;11:e43053. doi:10.2196/43053
4. Verma N, Mamlin B, Flowers J, Acharya S, Labrique A, Cullen T. OpenMRS as a global good: Impact, opportunities, challenges, and lessons learned from fifteen years of implementation. *Int J Med Inform*. May 2021;149:104405. doi:10.1016/j.ijmedinf.2021.104405
5. OpenEMR. Accessed Sept 20, 2019. https://www.open-emr.org/
6. Jing X, Min H, Gong Y, et al. A clinical decision support system (CDSS) ontology to facilitate portable vaccination CDSS rules: preliminary results. presented at: AMIA 2021; Oct 30 – Nov 3, 2021 2021; San Diego, CA.
7. Goli R, Hubig N, Min H, et al. Keyphrase Identification Using Minimal Labeled Data with Hierarchical Context and Transfer Learning. *Artificial Intelligence in Medicine, under review*. 2023;doi: https://medrxiv.org/cgi/content/short/2023.01.26.23285060v2
8. Jing X, Indani A, Hubig NC, et al. A systematic approach to configuring MetaMap for optimal performance. *Methods Inf Med*. May 25 2022;doi:10.1055/a-1862-0421
9. Wilkinson MD, Dumontier M, Aalbersberg IJ, et al. The FAIR Guiding Principles for scientific data management and stewardship. *Scientific Data*. 2016/03/15 2016;3(1):160018. doi:10.1038/sdata.2016.18
10. Jing X, Min H, Gong Y, et al. Literature on ontologies applied in clinical decision support systems demonstrates global collaborations. IOS; 2023:

**Fig. 1 (abstract O13).**
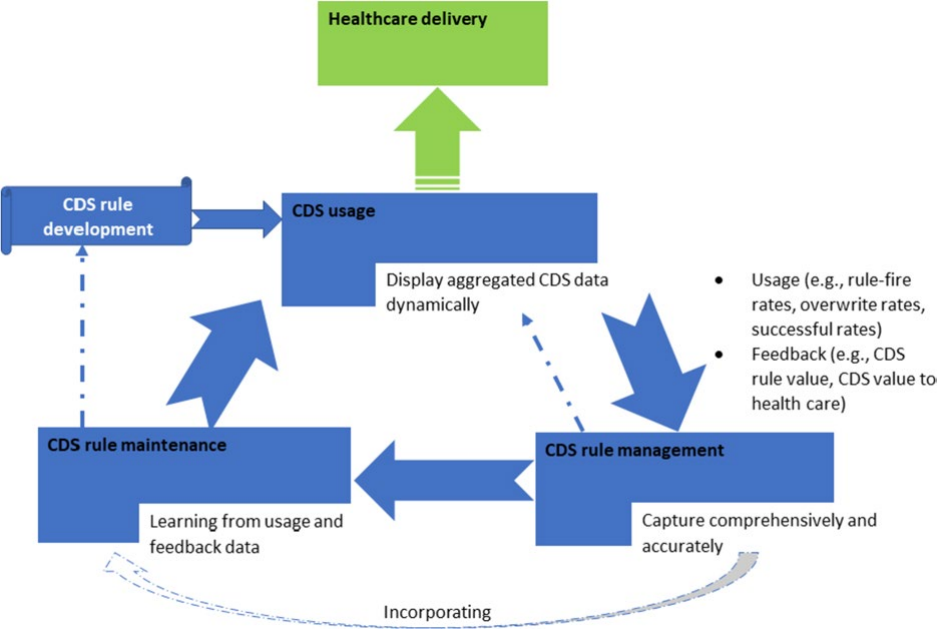
Conceptual model of CDS rule development, management, maintenance, and usage in healthcare delivery

**Fig. 2 (abstract O13).**
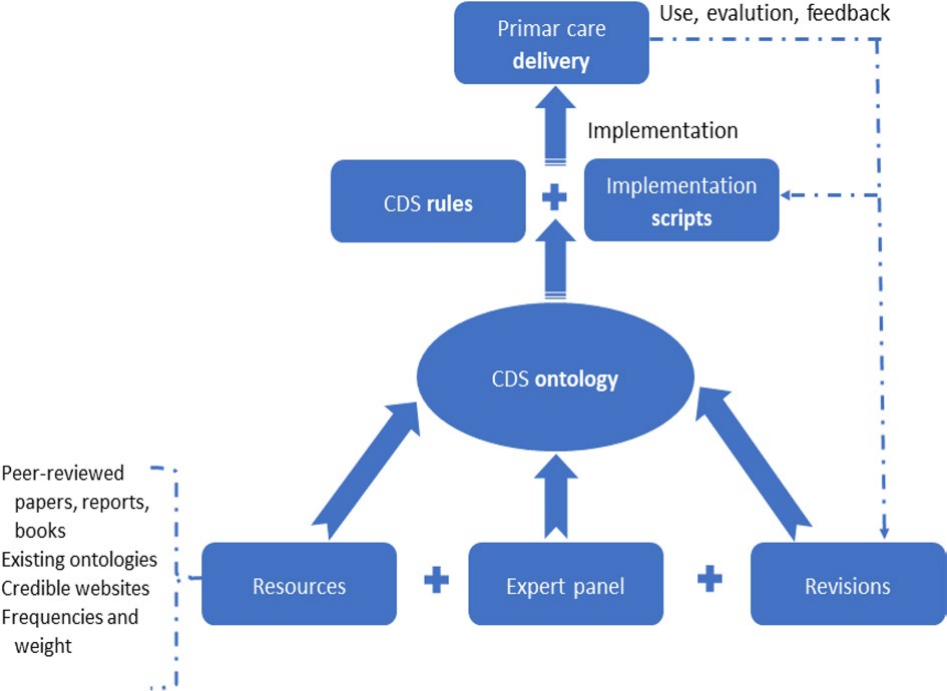
CDS ontology development, usage, implementation, and revision cycle and their roles in primary care

## O14 Leveraging smartphone technology to enhance patient-centered HIV care and treatment services in Nigeria’s most populous city: the Jolly-95 app experience

### Dimgba K. Kalu, David I. Udanwojo, Yewande I. Bamgbade, Obioma A. Azurunwa, Abimbola S. Phillips, Pius I. Christopher-Izere, Francis O. Ogirima, Collins O. Imarhiagbe, Bolanle O. Oyeledun

#### Centre for Integrated Health Programs, FCT Abuja, Nigeria

##### **Correspondence:** Obioma Ezebuka (oezebuka@cihpng.org)

*BMC Proceedings 2023*, **17(Suppl 19):**O14

**Issues:** The COVID-19 pandemic brought many disruptions to the health system with a marked effect on the HIV care and treatment continuum causing a reduction in clinic visits and in-person client engagement.^1^ Lagos – the most populous city in Nigeria and having an estimated 3.4 million people with HIV (PWHIV)^2^ – was the worst hit by the COVID-19 pandemic, with 40% of total cases reported in Nigeria.^3^ As of September 2021, there were more than 27,000 HIV program-supported sites in the state, this presented a challenge to continuity in HIV treatment and viral suppression. According to the National Communication Commission report, in 2022, there were approximately 154 million mobile internet subscribers in Nigeria, rising to 158 million in April 2023.^4^ Leveraging smartphone technology as a tool for delivering accurate and up-to-date information about HIV prevention, testing, treatment, and adherence,^5^ we developed and deployed the Jolly-95 App – an innovative strategy in engaging HIV clients to mitigate interruptions in treatment as part of differentiated service delivery models.

**Project:** The Jolly-95 App (Figure 1) is a self-service mobile app designed to sustain patient engagement in care and improve access to HIV support services. Patients with mobile phones were encouraged to sign up to gain access to app features including patient treatment information (antiretroviral regimen, current viral load), appointment scheduling, self-care management, in-app chat, and facilitated referrals to support services. Trained client service operatives provide real-time support for in-app requests and messaging, with end-to-end encryption to ensure data security.

**Lessons learned:** After 33 weeks of pilot implementation (10th February to 29th September 2022), 1,672 (26%) of 6381 clients who visited the clinic were assisted to download the app. All (100%) had logged in at least once to activate the appointment reminder features. There were more females (69%) than males (31%) among the users, with the greatest proportion (37%) of the users between age 40-49 years and the lowest (8%) between ages 10-29 years. The in-app chat was the most used feature (86%). Feedback via in-app chat showed that clients found the app relevant to their care and made remote access to HIV services easier. Of the 1,672 users, 1573 (94%) were still active in treatment six months after downloading the app. Of 1,044 users who had viral load tests done during the review period, 982 (94%) were virally suppressed. Limitations encountered in utilization included privacy concerns expressed among clients, and data cost to clients.

Integrating the Jolly-95 app into the HIV program can enhance patient-centered care and service quality which will result in improved patient outcomes including continuity in treatment. Additional modules to address HIV self-testing integration, and community pharmacy and facility locator services, are in development to enhance care and expand access.

**References**

1. Lagat H, Sharma M, Kariithi E, et al. Impact of the COVID-19 Pandemic on HIV Testing and Assisted Partner Notification Services, Western Kenya. AIDS Behav. 2020;24(11):3010-3013. doi:10.1007/s10461-020-02938-7
2. National Agency for the Control of AIDS. Nigeria HIV/AIDS Indicator and Impact Survey. Abuja, Nigeria: National Agency for the Control of AIDS; 2018.
3. Amzat J, Aminu K, Kolo VI, Akinyele AA, Ogundairo JA, Danjibo MC. Coronavirus outbreak in Nigeria: Burden and socio-medical response during the first 100 days. Int J Infect Dis. 2020; 98:218-224. doi: 10.1016/j.ijid. 2020.06.067
4. Nigerian Communications Commission. Industry Overview - Statistics & Reports [Internet]. Abuja, Nigeria: Nigerian Communications Commission; c2023 [last update June 02, 2023; cited June 19, 2023]. Available from: https://ncc.gov.ng/statistics-reports/industry-overview#view-graphs-tables-8
5. Okop KJ, Abubakar IS, Okure AO. Mobile phone usage pattern among caregivers attending a tertiary health facility in Nigeria. J Mobile Technol Med. 2014;3(3):23-32.

**Fig. 1 (abstract O14).**
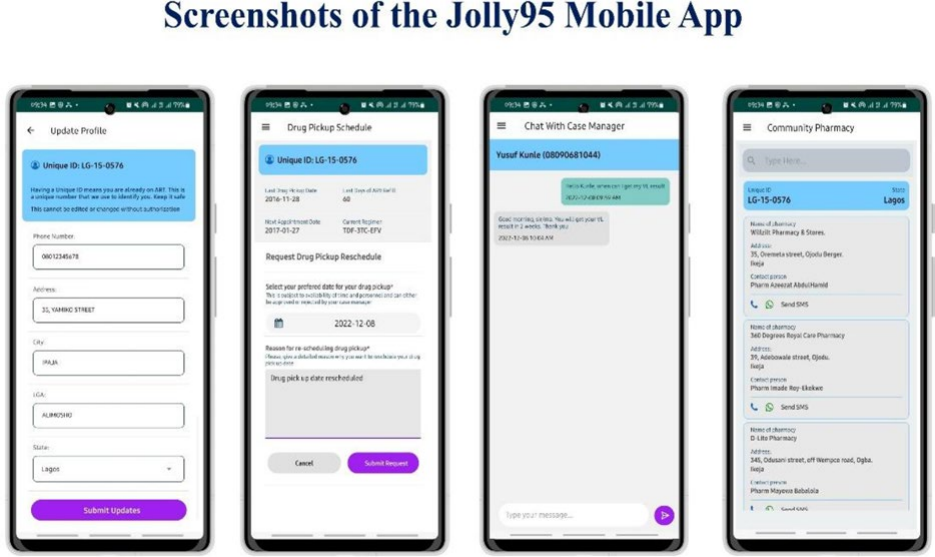
Screenshot of the Jolly95 mobile app

## O15 Youth-focused tobacco prevention through a novel advertising, sales, and social media tracking dashboard

### Jaron Hoani King (kingjh@dhec.sc.gov)

#### South Carolina Department of Health and Environmental Control, Columbia, SC, USA

##### Arnold School of Public Health, Columbia, SC, USA

*BMC Proceedings 2023*, **17(Suppl 19):**O15

**Abstract**

Following 2019 outbreak in THC-containing vaping products, South Carolina identified a distinct and urgent need to innovate tobacco control surveillance within the state. For reference, the youth vaping prevalence more than doubled in just 4 years. It became clear that vapes were quickly becoming the dominant form of nicotine used by young people, and existing public health surveillance systems were ill-equipped to monitor the unregulated market of electronic cigarettes. Given this background, the South Carolina Division of Tobacco Prevention and Control (DTCP) and external partner Research Triangle Institute (RTI) explored potential solutions to surveillance that allowed for more nimble adjustment and monitoring of this rapidly shifting nicotine product landscape. To help track and monitor emerging electronic nicotine delivery systems (ENDS) products, RTI developed the ENDS Tracker in coordination and participation with the SC DTCP. The ENDS Tracker is a data dashboard that collects, analyzes, and synthesizes multiple data sources to provide a comprehensive illustration of marketing and sales trends for emerging ENDS products. The dashboard includes data on (1) South Carolina retail sales of tobacco products (ENDS, cigars, cigarettes, smokeless, roll your own, and cessation products); (2) ENDS advertising in the United States and in South Carolina markets; (3) Google search trends for emerging ENDS products in South Carolina; (4) recent Twitter posts from ENDS marketing accounts; (5) recent Instagram posts from ENDS brands; and (6) recent posts from ENDS-related Reddit communities. The sales and advertising data are updated quarterly while all other data sources are updated in real-time. The dashboard was designed by an interdisciplinary team of tobacco control scientists, communication scientists, programmers, and data scientists. The ENDS Tracker employs a user-center design approach and presents the data in a graphics based, easy to understand, and customizable format. The evaluation methods of this surveillance tool are still in development. Current formative evaluations have mostly involved gathering input from subject matter experts in various program areas in the SC DTCP. The feedback mechanism to improve the tool has been formal quarterly meetings between SC DTCP and RTI coupled with regular communications over email as any issues or suggestions for improvement arise. Some of potential performance measures that may be used over the next 5 years will be (1) the number of scholarly articles and presentations; (2) the number and reach of earned media from news coverage of the partnership/tool; (3) a percentage decrease in social media posts with a positive view of youth ENDS use; and (4) decreases in combustible tobacco and ENDS sales. Additional performance measures are being considered. The ENDS Tracker is a data dashboard for public health professionals and stakeholders that collects, analyzes, and synthesizes multiple data sources to provide a comprehensive illustration of marketing and sales trends for emerging ENDS products.

## O16 Prognostic models for sepsis built on small datasets

### Chunyan Li^1^, Lu Wang^2^, Kexun Li^2,3^, Hongfei Deng^2^, Yu Wang^2^, Li Chang^2^, Ping Zhou^2^, Jun Zeng^2,3^, Mingwei Sun^2,3^, Hua Jiang ^2,3^, and Qi Wang^1^

#### ^1^Department of Mathematics, University of South Carolina, Columbia, SC, USA; ^2^Institute for Emergency and Disaster Medicine, Sichuan Provincial People’s Hospital, University of Electronic Science and Technology of China, Chengdu, China; ^3^Sichuan Provincial Research Center for Emergency Medicine and Critical Illness, Sichuan Provincial People’s Hospital, University of Electronic Science and Technology of China, Chengdu, China

##### **Correspondence:** Hua Jiang (jianghua@uestc.edu.cn); Qi Wang (qwang@math.sc.edu)

*BMC Proceedings 2023*, **17(Suppl 19):**O16

**Background and Objectives:** Sepsis is one of the common causes of death in intensive care units (ICU). A reliable prognostic model based on short-term historical records would enable clinicians to make proper clinical decisions to improve clinical outcomes for septic patients at the ICU. Patients’ biomarkers collected in longitudinal forms at the ICU are important for the clinical diagnosis and on-time treatment of sepsis for the ICU patients although they are often available in a high-dimensional and yet small dataset. The aim of this study is to develop a machine-learning (ML) modeling framework for developing prognostic tools based on patient’s short-term historical records by exploring the class-imbalanced longitudinal data of a small group of septic patients.

**Methods:** A machine-learning framework is proposed in which ML-ready data in the form of concatenated triplets is generated by sliding the window with width k from the patient’s longitudinal data. It greatly enriches the data size. Each concatenated triplet consists of a patient’s static data, the k-day consecutively longitudinal data, and the clinical outcome. Then the structured ML-ready data are used to train a set of selected classifiers in combination with appropriate sampling strategies and dimension reduction techniques to build the prognostic models. Eventually, the classifiers are evaluated on new collected dataset to make it practically usable. We illustrate the approach using five well-known classifiers: K nearest neighbors (KNN), Logistic Regression (LR), Support Vector Machine (SVM), Random Forest (RF), and Extreme Gradient Boosting (XGBoost) coupled with three sampling strategies (undersampling, SMOTE-NC oversampling and ensemble undersampling) and two dimension reduction techniques (RF and XGBoost). ROC AUC and a new indicator, γ defined by F1 score on the external validation set, are used to assess the efficacy of the models.

**Results:** Five prognostic classifiers/models are built on the re-engineered ML-ready dataset accounting for 10 selected dynamic features. XGBoost (ROC AUC=0.777, F1 score=0.694) and RF (ROC AUC=0.769, F1 score=0.647) combined with ensemble undersampling strategy outperform their peers in the external validation. We show that the modeling approach can greatly improve the accuracy and generalizability of the standard classifiers. The improvement in ROC AUC and overfitting are 6.66%, 54.96% and 0.52%, 77.72% for RF and XGBoost, respectively.

**Conclusion:** This study proposes an effective modeling framework for using small, class imbalanced, and high-dimensional dataset to clinical prognosis of septic patients with respect to their mortality. This innovative framework enables standard classifiers to use small datasets to achieve relatively high predictability by designing new structured datasets and combining sampling strategies and dimension reduction techniques. It provides a useful prognostic tool based on short-term patient longitudinal data and an example for applying machine learning methods to small data problems in medicine.

This study has been reviewed by the Medical Ethics Committee of Sichuan Provincial People’s Hospital (No. 266 in 2021) and registered in the China Clinical Trial Registration Center (Clinical Registration No.: ChiCTR2200056316). Since it was an observational study on historical data and would not interfere with the treatment plan of the patients, the ethics committee agreed to waive the informed consent.

## O17 The utility of a Bayesian predictive model to forecast neuroinvasive West Nile virus disease in the United States, 2022

### Maggie S. J. McCarter^1^, Stella Self^1^, Kyndall C. Dye-Braumuller^1^, Christopher Lee^2^, Huixuan Li^1^, Melissa S. Nolan^1^

#### ^1^Department of Epidemiology and Biostatistics, Arnold School of Public Health, University of South Carolina, Columbia, SC, USA; ^2^Department of Computer Science and Engineering, College of Engineering and Computing, University of South Carolina, Columbia, SC, USA

##### **Correspondence:** Melissa Nolan (msnolan@mailbox.sc.edu)

*BMC Proceedings 2023*, **17(Suppl 19):**O17

**Study Objectives:** Arboviruses (arthropod-borne viruses) are an emerging global health threat that is rapidly spreading as climate change, international travel, and landscape fragmentation impact local ecologies. Since its initial detection in 1999, West Nile virus (WNV) has shifted from being a novel disease to an established arbovirus in the United States^1^. Subsequently, more than 25,000 cases of West Nile Neuro-invasive Disease (WNND) have been diagnosed, cementing WNV as public health priority^2^. Given its recent emergence in the United States, high-risk ecologies are largely underdefined, making targeted public health interventions challenging. Therefore, we developed a model to predict county-level WNND human cases in the contiguous USA.

**Methods:** Using the Centers for Disease Control and Prevention ArboNET WNND data from 2000 - 2021, we predicted WNND human cases using a Bayesian spatiotemporal negative binomial regression model. The model includes environmental, climatic, and demographic factors, as well as host species distribution. An integrated nested LaPlace approximation (INLA) approach was used to fit our model^3^. We fit the model by removing variables that were not found to be statistically important individually until all variables were statistically important. We then added the removed variables back in individually, calculated the mean and median square prediction error, and kept those that improved the mean square prediction error. To assess model prediction accuracy, annual counts were withheld, forecasted, and compared to observed values. The validated models were then fit to the entire dataset for 2022 predictions.

**Results:** After model selection, our final model was able to predict 2021 cases with a median square prediction error of 0.006 cases^2^. After variable selection, the model showed accurate prediction of historical WNND cases in most counties, though the model can be improved on counties with very large populations (Figure 1). Following our assessment of model accuracy, we successfully predicted WNND incidence for the year 2022 (Figure 2).

**Discussion:** This study, utilizing Bayesian inference, is one of the first studies to predict human mosquito-borne disease for the continental USA at the county level, and introduces concepts that have application for future studies. This proof-of-concept mathematical, geospatial modeling approach has proven utility for national health agencies seeking to allocate funding and other resources for local vector control agencies tackling WNV and other notifiable arboviral agents.

**References**

1. Artsob H, Gubler DJ, Enria DA, et al. West Nile Virus in the New World: trends in the spread and proliferation of West Nile Virus in the Western Hemisphere. *Zoonoses Public Health*. 2009;56(6-7):357-369. doi:10.1111/j.1863-2378.2008.01207.x
2. Ronca SE, Murray KO, Nolan MS. Cumulative Incidence of West Nile Virus Infection, Continental United States, 1999–2016. *Emerg Infect Dis*. 2019;25(2):325-327. doi:10.3201/eid2502.180765
3. Rue H, Martino S, Chopin N. Approximate Bayesian inference for latent Gaussian models by using integrated nested Laplace approximations. *J R Stat Soc Ser B Stat Methodol*. 2009;71(2):319-392. doi:10.1111/j.1467-9868.2008.00700.x

**Fig. 1 (abstract O17).**
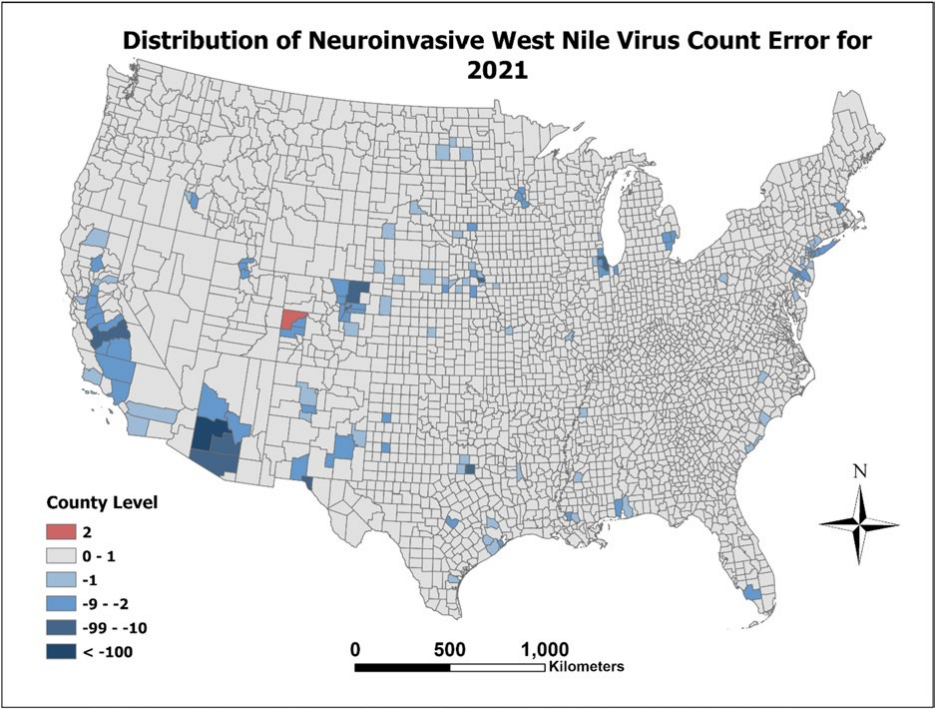
Distribution of Neuroinvasive West Nile Virus Count Error for 2021

**Fig. 2 (abstract O17).**
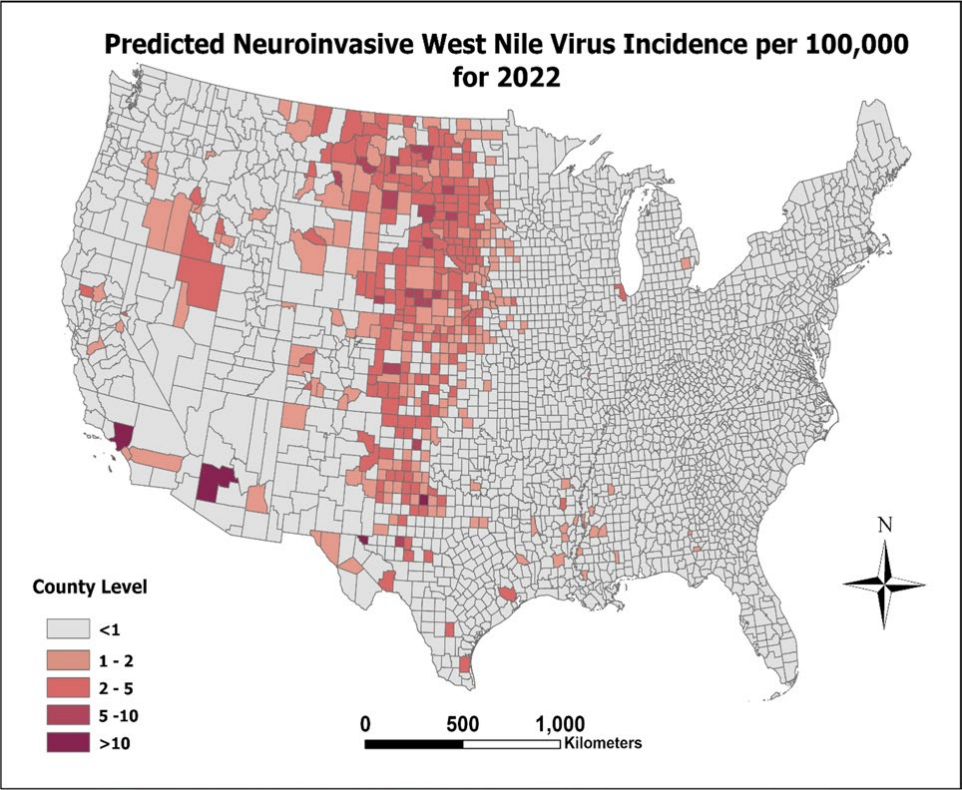
Predicted Neuroinvasive West Nile Virus Incidence per 100,000 for 2022

## O18 Using smartphone-based place visitation big data to improve health measure estimation

### Huan Ning^1, 2^, Zhenlong Li^1, 2^, Fengrui Jing^1, 2^, Shan Qiao^2^, Xiaoming Li^2^

#### ^1^Geoinformation and Big Data Research Lab, Department of Geography, University of South Carolina, Columbia, SC, USA; ^2^Big Data Health Science Center, University of South Carolina, Columbia, SC, USA

##### **Correspondence:** Huan Ning (hning@email.sc.edu)

*BMC Proceedings 2023*, **17(Suppl 19):**O18

**Abstract Narrative:** Resident routing activity information extracted from place visitation big data improves the model explanation power for health measure estimation at the census tract level^1,2^, especially for the measures of health risk behaviors that involve place visitation such as *Binge Drinking* and *Visits to Doctor for Routine Checkup*.

**Study Objectives**: Health measures for small areas (i.e., census tract level health outcomes, prevention, health risk behaviors, and health status) can support effective public health planning for policymakers^3–7^. Social determinants of health (SDOH) factors are usually applied to generate health measure estimation^3^. However, SDOH lacks consideration of resident routine activities that may affect health measures^8^. For example, more fitness center visitation might relieve depression, and bar trips might increase binge drinking. This study investigated whether the additional resident routine activity information improves health measure estimation in areas with small populations (e.g., census tract) in the United States (US).

**Method**: We collected SDOH data and resident routine activity information at the tract level in the US and conducted hierarchical linear regression. SDOH contains 12 variables derived from American Community Survey 5-Year Estimates 2019 (ACS 2019), such as race, education, and income. Routine activity information consisted of per capita visitors of tracts to 120 categories of places (e.g., restaurants and parks) in 2019, derived from smartphone-based place visitation big data (>1 billion records) obtained from SafeGraph^9^. The health measures were extracted from the Population-Level Analysis and Community Estimates (PLACES) dataset^10^. Hierarchical regression using SDOH with and without visitation data was conducted to examine whether including resident routine activity information improves the model explanation power to the 22 health measures in PLACES.

**Preliminary Results**: The results of hierarchical regression showed that SDOH factors contributed the most variance to the 22 health measures (mean *r*^*2*^ = 0.732). Place visitation data increased the prediction performance of all health measures (mean *r*^*2*^ increased by 6%). The performance improvements varied among health measures. Seven health measures that benefited more than 10% of performance increase were *Binge Drinking* (39.2%), *Visits to Doctor for Routine Checkup* (22.1%), *Talking Medicine for High Blood Pressure Control*, *Taking Medicine For High Blood Pressure Control* (19.2%), *High Cholesterol* (18.8%), *Depression* (13.4%), *Obesity* (11.2%), and *Cholesterol Screening* (10.3%).

**Discussion:** Resident routing activity information extracted from place visitation big data improves the model explanation power for health measure estimation at the census tract level, especially for the measures of health risk behaviors that involve place visitation such as *Binge Drinking* and *Visits to Doctor for Routine Checkup*. Place visitation data can be an auxiliary data source to traditional SDOH factors for health measure estimation. This study also provided researchers with new insights into the association between the place-based health measures and resident routing activities.

**References**

1. Zhou RZ, Hu Y, Tirabassi JN, Ma Y, Xu Z. Deriving neighborhood-level diet and physical activity measurements from anonymized mobile phone location data for enhancing obesity estimation. *Int J Health Geogr*. 2022;21(1):22. doi:10.1186/s12942-022-00321-4
2. Jing F, Li Z, Qiao S, Ning H, Zhou S, Li X. Association between immigrant concentration and mental health service utilization in the United States over time: A geospatial big data analysis. *Health Place*. 2023;83:103055. doi:10.1016/j.healthplace.2023.103055
3. Zhang X, Holt JB, Lu H, et al. Multilevel regression and poststratification for small-area estimation of population health outcomes: a case study of chronic obstructive pulmonary disease prevalence using the behavioral risk factor surveillance system. *Am J Epidemiol*. 2014;179(8):1025-1033. doi:10.1093/aje/kwu018
4. Zhang X, Holt JB, Yun S, Lu H, Greenlund KJ, Croft JB. Validation of Multilevel Regression and Poststratification Methodology for Small Area Estimation of Health Indicators From the Behavioral Risk Factor Surveillance System. *Am J Epidemiol*. 2015;182(2):127-137. doi:10.1093/aje/kwv002
5. Feng C, Jiao J. Predicting and mapping neighborhood-scale health outcomes: A machine learning approach. *Comput Environ Urban Syst*. 2021;85:101562. doi:10.1016/j.compenvurbsys.2020.101562
6. Wang Y. Using 3 Health Surveys to Compare Multilevel Models for Small Area Estimation for Chronic Diseases and Health Behaviors. *Prev Chronic Dis*. 2018;15. doi:10.5888/pcd15.180313
7. Ning H, Li Z, Qiao S, et al. Revealing geographic transmission pattern of COVID-19 using neighborhood-level simulation with human mobility data and SEIR model: A case study of South Carolina. *Int J Appl Earth Obs Geoinformation*. 2023;118:103246. doi:10.1016/j.jag.2023.103246
8. Qiao S, Li Z, Zhang J, Sun X, Garrett C, Li X. Social Capital, Urbanization Level, and COVID-19 Vaccination Uptake in the United States: A National Level Analysis. *Vaccines*. 2022;10(4):625. doi:10.3390/vaccines10040625
9. SafeGraph. Patterns | SafeGraph Docs. SafeGraph. Published November 2022. Accessed December 21, 2022. https://docs.safegraph.com/docs/monthly-patterns
10. Greenlund KJ. PLACES: Local Data for Better Health. *Prev Chronic Dis*. 2022;19. doi:10.5888/pcd19.210459

## O19 Underrepresented minorities (URM) academic career development and data science applied to health luncheon

### Miranda Nixon (mc95@mailbox.sc.edu)

#### Big Data Health Science Center, University of South Carolina, Columbia, SC, USA; Department of Health Promotion, Education, and Behavior, University of South Carolina, Columbia, SC, USA

*BMC Proceedings 2023*, **17(Suppl 19):**O19

**Background:** Nationwide, racial and ethnic minority students comprise approximately 39% of the college population but earn approximately only 17% of bachelor’s degrees and 13% of doctoral degrees in the life sciences.^1^ The percentage of undergraduate public health degrees conferred to racial/ethnic minority groups decreased from 23% to 18% from 2003 to 2012.^2^ A promising approach to increasing the diversity of the big data analytics workforce in infectious disease research is to promote big data analytics research training and education among students from diverse backgrounds in the early stages of their academic training.

**Methods/Design:** To engage the local Historically Black Colleges and Universities (HBCU) community and underrepresented minority (URM) students at the University of South Carolina and other South Carolina institutions, and to increase the participation of URM students in the data science applied to health arena, the 2023 National Big Data Health Science Conference planning committee invited faculty member advisors and at least 5 URM students from USC, Allen University, Benedict College, Claflin University, Voorhees University and other institutions to attend the 2023 National Big Data Health Science Conference and participate in a URM Academic Career Development and Data Science Applied to Health Luncheon.

This inaugural event was held in conjunction with the 4^th^ annual National Big Data Health Science Conference on Friday, February 10^th^, 2023. It was an opportunity for URM students interested in pursuing academic-related careers to gather with URM faculty and leadership and discuss opportunities in data science, the institutional value of diversity and URM involvement in academia, network and connect with potential mentors, and learn about resources and gain insight into the challenges and opportunities URM traditionally encounter when embarking on academic-related career paths. Attendees received complementary registration to attend the national conference; access to 100% of the conference programming, including presentations by esteemed experts including academic, industry and government leaders and focused breakout sessions and workshops; dozens of networking opportunities; and a seat at the URM Academic Career Development and Data Science Applied to Health Luncheon.

**Discussion:** The inaugural event attracted 45 attendees coming from institutions across South Carolina. The majority of attendees were undergraduate students with biomedical or health science related majors. Also in attendance were faculty representatives and advisors from the attending HBCUs including Benedict College, Claflin University, Allen University and Voorhees University. Leadership from USC were also in attendance. The hour-long working luncheon included presentations from USC leadership and a guided Q&A discussion with URM faculty. This event will be featured again at the 5^th^ Annual National Big Data Health Science Conference on February 2-3, 2024.

**References**

1. National Institute of Allergy and Infectious Diseases (NIAID). *PAR-21-258: NIAID Research Education Program Advancing the Careers of a Diverse Research Workforce (R25 Clinical Trial Not Allowed)*. July 16, 2021.
2. Goodman MS, Plepys CM, Bather JR, Kelliher RM, Healton CG. Racial/ethnic diversity in academic public health: 20-year update. *Public Health Reports.* 2020;135(1):74-81.

## O20 Leveraging electronic health records for big data research

### Jihad S. Obeid (jobeid@musc.edu)

#### Department of Public Health Sciences, Medical University of South Carolina, Charleston, SC, USA

*BMC Proceedings 2023*, **17(Suppl 19):**O20

**Abstract**

Secondary use of electronic health records (EHR) data offers a valuable resource for data-driven biomedical research. With years of widespread adoption of EHRs nationally and recent advances in computational resources, big data methods including artificial intelligence (AI) research have gained significant traction. Several academic health systems have established integrated data repositories optimized for research with appropriate oversight. Examples of research leveraging EHR data include the characterization of specific clinical phenotypes and predictive modeling for patient outcomes. A good portion of the information within the EHR is in the form of unstructured text-based clinical notes. A variety of natural language processing (NLP) pipelines have been developed and used for information extraction. In recent years, significant strides have been made in deep learning clinical text-based approaches. Working with these models is far less daunting than it used to be, thanks to machine learning frameworks—such as Google’s open-source framework, TensorFlow.

A research infrastructure for using EHR data enables collaborations in regional and national data networks, as well as AI-based research with focus on unstructured data in clinical records. We have used different types of clinical text classifiers in a variety of clinical domains, using both traditional machine learning and deep learning algorithms. This works demonstrates the often-superior performance of deep learning algorithms such as convolutional neural networks and highlights associated challenges related to interpretability of the results. We have demonstrated that deep learning text classifiers are highly effective for e-phenotyping tasks. Their effectiveness with predictive tasks, such as the prediction of suicidal behavior, is competitive when compared with traditional models using structured EHR data, albeit not as effective as in phenotyping applications. We have also demonstrated text mining approaches such as variable importance or word overrepresentation analyses – may yield insight into the characteristics of phenotype-associated keywords in clinical text and possible symptomatology, e.g., the appearance of words such as “smell”, “taste”, “loss” in a cohort of patients who tested positive for COVID-19. The de-identification of clinical text using established NLP methods does not seem reduce this performance of classifiers. The impact of pre-training using self-supervised algorithms such as word2vec, BERT and newer language models, as well as the impact of transfer learning across institutional boundaries is still being investigated.

## O21 Leveraging the continuity in treatment dashboard analytics to retain persons living with HIV on ART care and treatment in Nigeria: the Lagos ART surge experience

### Idowu G. Oluwasola, Obioma A. Azurunwa, Ikponwonmsa Omogun, Collins O. Imarhiagbe, David I. Udanwojo, Abimbola S. Phillips, Bolanle O. Oyeledun

#### Centre for Integrated Health Programs, FCT Abuja, Nigeria

##### **Correspondence:** Abimbola S. Phillips (aphillips@cihpng.org)

*BMC Proceedings 2023*, **17(Suppl 19):**O21

**Background:** In 2018, there were 1.8 million people with HIV (PWHIV) with only 1.1 million (64%) receiving antiretroviral therapy (ART) in Nigeria.^1^ According to the continuum of care demands, PWHIV are identified, started, and retained on treatment using the UNAIDS 95:95:95 treatment goal.^2^ In April 2019, through the U.S. President’s Emergency Plan for AIDS Relief (PEPFAR), the Centre for Disease Control and Prevention (CDC) launched an 18-month ART Surge program in nine Nigerian states including Lagos to rapidly increase the number of PWHIV receiving ART. With HIV prevalence in Lagos at 1.3% and an estimated 120,000 PLHIV, half of which were not on treatment,^3^ poor retention has been a concern for the surge program in Lagos. Centre for Integrated Health Programs with funding from PEPFAR/CDC developed an innovative Continuity in Treatment (CIT) retention dashboard to monitor near real-time tracking of all PLHIVs on treatment and prevent drop offs, increasing viral suppression rate, which minimizes HIV transmission, and thereby moving Nigeria closer to epidemic control.

**Project description/Methods:** CIT was previously based on routine assessments of patient files but has been updated to use electronic medical records to generate data on treatment transitions for program planning. Poor data on interruptions in treatment (IIT), a reliable indication of retention, could not be detected accurately before the CIT-Retention dashboard was created in June 2020. The dashboard, which runs on Microsoft Power BI software, provides near-real-time analysis, reports, and insights into retention and treatment. This involved four focused data science implementations: (1) Data Flow (data collection, storage and accessing), (2) Data Curation (cleaning, modeling, and evaluation), (3) Data Analytics (statistical analysis, data modeling and stimulations, and data visualizations) and (4) Data Visualization (profiling, presentation, and GIS analysis).

**Results/lessons learned:** Prior to the implementation of CIT-Retention dashboard (at the end of the second quarter in 2020), IIT rate remained high at 5.1% with viral load (VL) coverage and viral suppression at 80% and 95%, respectively. Post- CIT-Retention dashboard implementation, there was a sharp decline of IIT rate to 2.6% (49% reduction), followed by a continuous and sustained decline in IIT rates to 1.1% by June 2022 with improved VL coverage and viral suppression of 92% and 96%, respectively (see figures 1 and 2). The CIT dashboard was useful for near real-time review and automated analysis of patient level data for tracking and monitoring of outcomes (e.g., VL eligibility, sample collection, missed appointment and IIT), and improved program decision making.

**Conclusion:** The CIT-Retention Dashboard can serve as a critical tool to identify and follow up persons in care that are likely to experience IIT using predictive analysis from historical data. This has the potential to significantly improve retention among PLHIV in care, and consequently improve clinical outcomes.

**References**

1. Federal Ministry of Health, Nigeria. Nigeria HIV/AIDS Indicator and Impact Survey (NAIIS) 2018: Technical Report. Abuja, Nigeria. October 2019.
2. Frescura L, Godfrey-Faussett P, Feizzadeh A A, El-Sadr W, Syarif O, Ghys PD; on and behalf of the 2025 testing treatment target Working Group. Achieving the 95 95 95 targets for all: A pathway to ending AIDS. PLoS One. 2022 Aug 4;17(8): e0272405. doi: 10.1371/journal.pone.0272405. PMID: 35925943; PMCID: PMC9352102.
3. https://pdf.usaid.gov/pdf_docs/PA00XBDH.pdf. Accessed August 2020.

**Fig. 1 (abstract O21).**
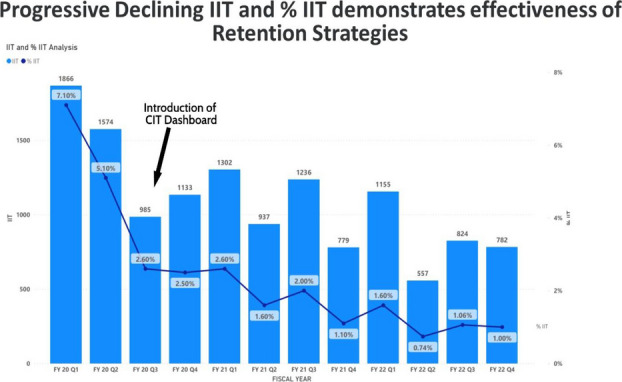
Progressive decrease in interruption in treatment (IIT) following deployment of retention strategies

**Fig. 2 (abstract O21).**
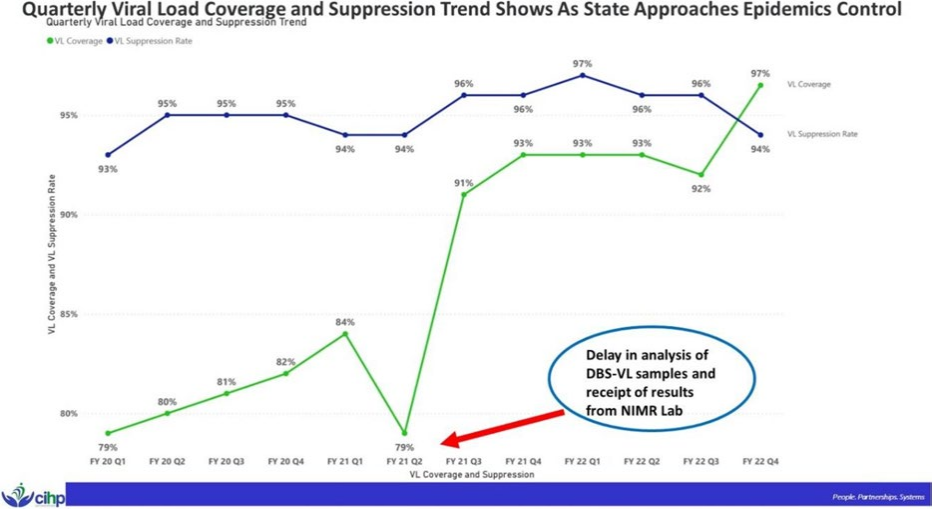
Trend in quarterly viral load coverage and suppression as the State gets closer to epidemic control

## O22 Harnessing big heterogeneous data to evaluate the potential impact of HIV responses among key populations in generalized epidemic settings in Sub Saharan Africa: the Boloka Data Repository

### Refilwe Nancy Phaswana-Mafuya^1,2^, Edith Phalane^1,2^, Katharine S. Journeay^3^ , Haley I. Sisel^3^, Claris Siyamayambo^1,2^, Betty Sebati^1,2^, Francois Wolmarans^3^, Katherine Rucinski^4^, Amrita Rao^4^, Kalai Willis^4^, Xiaoming Li^5^, Bankole Olatosi^5^, Stefan D. Baral^4^

#### ^1^South African Medical Research Council/University of Johannesburg (SAMRC/UJ) - Pan African Centre for Epidemics Research (PACER) Extramural Unit; ^2^Faculty of Health Sciences, Department of Environmental Health, University of Johannesburg, Johannesburg, South Africa; ^3^University of Johannesburg Technology Architecture & Planning, Johannesburg, South Africa; ^4^Key Populations Program, Center for Public Health and Human Rights, Johns Hopkins Bloomberg School of Public Health, Baltimore, MD; ^5^Big Data Health Science Center, University of South Carolina, Columbia, SC, USA

##### **Correspondence:** Refilwe Nancy Phaswana-Mafuya (refilwep@uj.ac.za)

*BMC Proceedings 2023*, **17(Suppl 19):**O22

“*The work reported herein was made possible through funding by the South African Medical Research Council through its Division of Research Capacity Development under the Mid-Career Scientist Programme using funding received from the South African National Treasury. The content hereof is the sole responsibility of the authors and do not necessarily represent the official views of the SAMRC”*

**Background:** Key Populations (KPs), including gay men and other men who have sex with men, female sex workers, transgender persons, people who use drugs, and incarcerated persons, bear a much higher burden of HIV compared to other adults of reproductive age^1-2^. There is no centralized system to exclusively gather and monitor data KP for HIV surveillance and programming^3^. The Boloka data repository is being developed as a mechanism for KP data storage. Harnessing big heterogeneous data on HIV for KPs is necessary to improve our understanding of HIV among KPs; assist in setting and monitoring programme targets^3-4^.

**Objective:** The overall objective of the study is to leverage and collate existing available KP HIV-related data, in South Africa from 2000 onwards

**Methods:** To achieve the stated objective, this study will undergo several stages as reflected in Figure 1.

**Progress Update:** This study is in the preliminary stages. Ethics approval from the University of Johannesburg Faculty of Health Sciences Research Ethics Committee has been secured and will be renewed yearly as required. A transdisciplinary and multi-institutional study team is in place. A project steering committee has been established. Research assistants, postgraduate students, and post-doctoral fellows who will utilize the data have been recruited, onboarded, and trained. There has been engagement with stakeholders to develop meaningful partnerships to facilitate collaboration and data sharing (stage 1). Data have been harnessed, including: routinely collected HIV programmatic data, published research data, and technical reports (stage 2). The data received was checked for accuracy, relevance, and quality to enable high impact analyses (stage 3). The data have been placed in a staging area prior to being stored in REDCap (stage 4). The University of Johannesburg (UJ), through its Information and Communication Services, secured REDCap license from the REDCap consortium. The license grants UJ permission to utilise the REDCap software, along with access to the consortium's support tools and resources. Currently, ICS is in the process of implementing the REDCap system in compliance with ICS Security standards and adhering to the best practices recommended for REDCap. Currently, ICS is in the process of implementing the REDCap system in compliance with ICS Security standards and adhering to the best practices recommended for REDCap. The Consortium Resources link has been shared to provide access to REDCap Training Materials. Through this platform, KP data will be securely uploaded into a centralised storage area that is managed and protected. Furthermore, authorised users and stakeholders will have the capability to generate customised reports and export the data to applications such as STATA for further dissemination (stage 5). Initial secondary data analyses using analytic methods attuned to the structure of available data, including cross-sectional and longitudinal analyses are being conducted to improve our understanding of HIV among KPs for a targeted response.

**Conclusion:** This work will advance scientific evidence on KPs, help prepare for, and respond more proactively and decisively to the persistent HIV epidemic^2-6^. Further, it will inform future research directions and become a credible institutional mechanism for epidemiological and public health training^2-6^.

**References**

1. The Joint United Nations Programme on HIV/AIDS (UNAIDS). 2022. Fact sheet 2022. Available from: https://www.unaids.org/sites/default/files/media_asset/UNAIDS_FactSheet_en.pdf (accessed 12 September 2022)
2. South African National AIDS Council (SANAC). National Strategic Plan for HIV, TB, STIs 2023-2028. Available from: https://sanac.org.za/wp-content/uploads/2023/05/NSP-Document.pdf (accessed on 09 May 2023)
3. South African National AIDS Council: Bello B, N dagurwa P, Omogiate S , L uwaca B, Motsieloa L . Republic of South Africa: 2020 Global AIDS Monitoring Report (GAM). 2021. Johannesburg: CESAR; 2021. Available from: https://sanac.org.za/south-africa-global-aids-monitoring_gam-report-2020/ (accessed on 23 May 2023)
4. Stone J, Mukandavire C, Boily MC et al. (2021) Estimating the contribution of key populations towards HIV transmission in South Africa. JIAS.24:e25650. 10.1002/jia2.25650.
5. Baral S, Beyrer C, Muessig K et al. Burden of HIV among female sex workers in low-income and middle-income countries: a systematic review and meta-analysis. Lancet Infect Dis. 2012; 12(7):538-549. 10.1016/S1473-3099(12)70066-X.
6. Long LC, Rosen S, Nichols B, Larson BA, Ndlovu N, Meyer-Rath G. Getting resources to those who need them: the evidence we need to budget for underserved populations in sub-Saharan Africa. J Int AIDS Soc. 2021 Jul;24 Suppl 3(Suppl 3):e25707. doi: 10.1002/jia2.25707.

**Fig. 1 (abstract O22).**
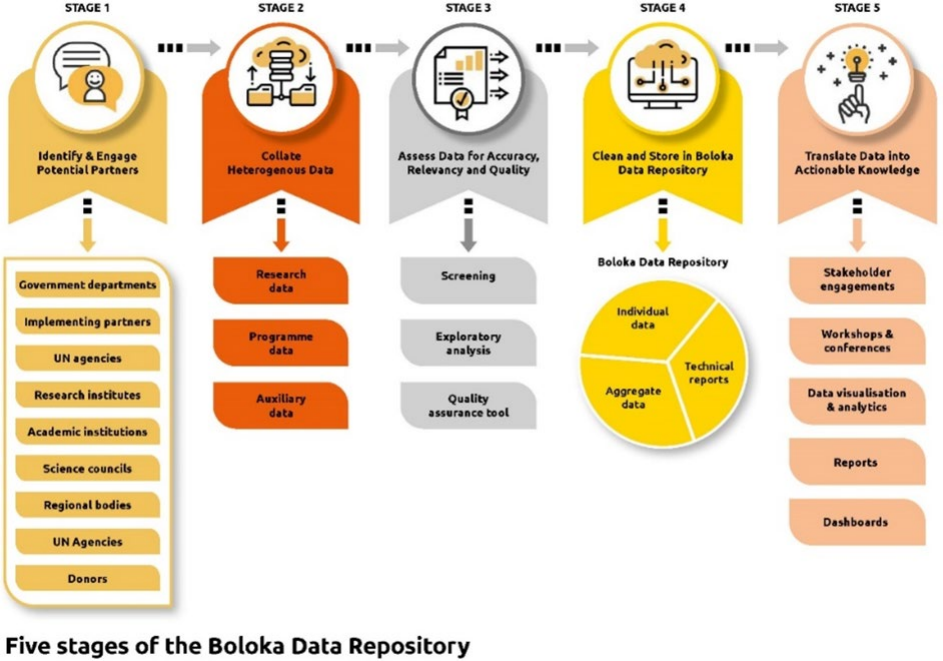
Five stages of the Boloka Data Repository

## O23 The incidence and evolving risk factors of diabetes among people with HIV: a population-based cohort study

### Gazi Sakir Mohammad Pritom^1,2^, Xueying Yang^1,2,4^, Haoyuan Gao^4^, Shujie Chen^1,3,4^, Jiajia Zhang^1,3,4^, Bankole Olatosi^1,3,5^, Xiaoming Li^1,2,3^

#### ^1^South Carolina SmartState Center for Health Care Quality, Arnold School of Public Health, University of South Carolina, Columbia, SC, USA; ^2^Department of Health Promotion, Education, and Behavior, Arnold School of Public Health, University of South Carolina, South Carolina, Columbia, SC, USA; ^3^Big Data Health Science Center, University of South Carolina, Columbia, SC, USA; ^4^Department of Epidemiology and Biostatistics, Arnold School of Public Health, University of South Carolina, Columbia, SC, USA; ^5^Department of Health Services Policy and Management, Arnold School of Public Health, University of South Carolina, Columbia, SC, USA

##### **Correspondence:** Gazi Sakir Mohammad Pritom (gspritom@email.sc.edu)

*BMC Proceedings 2023*, **17(Suppl 19):**O23

**Background and objective:** Prolonged life expectancy in people with HIV (PWH) might lead to an increased burden of a variety of comorbidities, including diabetes mellitus (DM)^1,2^. The present study aimed to describe the incidence of DM among PWH and investigate the trajectory of changes in traditional and HIV-related risk factors associated with DM incidence.

**Method:** This is a population-based cohort study, with data retrieved from integrated electronic health records in South Carolina (SC). Adults (age≥18) PWH diagnosed between 2008 and 2020 who were event-free at baseline were included. The association of HIV-related and traditional risk factors with incidence of DM during the overall study period and the follow-up time up to 5-, 8-, 13-year were investigated using multivariable logistic regressions.

**Results:** Among 9,115 PWH, the incidence of DM was 8.9 per 1000 person years. In the overall model, age (adjusted odds ratio [AOR]: 4.26, 95% Confidence Interval [CI]: 2.80, 6.48), hypertension (AOR: 2.96, 95%CI: 2.39,3.68), dyslipidemia (AOR: 1.508, 95%CI: 1.17,1.95), and obesity (AOR: 1.91, 95%CI: 1.49,2.44) were significant (p<0.05) risk factors for DM. While Injecting Drug Use (AOR: 0.61 95%CI: 0.39, 0.97) and HIV diagnosis year (AOR: 0.90, 95%CI: 0.88, 0.92) were found to be protective factors for DM. After stratified by three groups with different follow up time periods, age, dyslipidemia, and obesity were significant in only group 1(0-5^th^ follow up years) and group 2 (6-8^th^ follow up years). Hypertension was found significant in all three groups.

**Conclusion and implication:** DM incidence among PWH in SC is lower than previous estimates^3^. The underlying need for understanding how risk factors are evolving over time is highlighted with this study.

**Ethics approval**

This abstract reports findings from a larger study which has been reviewed by the Institutional Review Board at the University of South Carolina and was approved for research [USC IRB (#Pro00068124)].

**References**

1. May MT, Gompels M, Delpech V, et al. Impact on life expectancy of HIV-1 positive individuals of CD4+ cell count and viral load response to antiretroviral therapy. AIDS. 2014;28(8):1193-1202. doi: 10.1097/qad.0000000000000243
2. Deeks SG, Lewin SR, Havlir DV. The end of AIDS: HIV infection as a chronic disease. The Lancet. 2013;382(9903):1525-1533. doi: 10.1016/s0140-6736(13)61809-
3. Tripathi A, Liese AD, Jerrell JM, et al. Incidence of diabetes mellitus in a population-based cohort of HIV-infected and non-HIV-infected persons: the impact of clinical and therapeutic factors over time. Diabetic Medicine. 2014;31(10):1185-1193. doi: 10.1111/dme.12455

## O24 Association between patient-provider shared decision-making and use of pain-related complementary and integrative health modalities among adults with chronic noncancer pain, 2010-2017

### Yiwen Shih^1^, Peiyin Hung^1,2^

#### ^1^Department of Health Services Policy and Management, Arnold School of Public Health, University of South Carolina, Columbia, SC, USA; ^2^Big Data Health Science Center, University of South Carolina, Columbia, SC, USA

##### **Correspondence:** Yiwen Shih (yshih@email.sc.edu)

*BMC Proceedings 2023*, **17(Suppl 19):**O24

**Background:** Chronic noncancer pain (CNCP) affected every one in five US adults in 2019 and has been commonly prescribed opioids to manage pain. In 2016, Center for Disease Control and Prevention released guidelines for prescribing opioids for CNCP, recommending engaging complementary and integrative health (CIH) modalities to prevent initial opioids. Patient-provider shared decision-making could improve patients’ awareness of multimodal treatment plans. However, little is known about the relationship between patient-provider shared decision-making and uptake of CIH modalities. This study examined the association between shared decision-making and the use of pain-related CIH modalities among US adults with CNCP.

**Materials and methods:** This national retrospective cross-sectional study used data from the Medical Expenditure Panel Survey (MEPS), specifically Panel 15 (2010-2011) to 21 (2016-2017). International Classification of Diseases (ICD) and Clinical Classification codes were used to identify adults with CNCP. The primary outcome – dichotomous pain-related CIH modality use – was defined as having had at least one visit to chiropractors, acupuncturists, massage therapists, homeopathic/naturopathic/herbalists, and other complementary/alternative care providers for managing or addressing CNCP. Patient-provider shared decision-making was measured with a Likert scale (never, sometimes, usually, always). Rao-Scott Chi-square tests and a survey-weighed generalized logit model were applied, controlling for individuals’ sociodemographic factors, clinical conditions, and health behavior factors.

**Results:** A total of 9,482 adults with CNCP (corresponding to a weighted population of 206,110,116) during 2010-2017 were racially diverse (Hispanic: 10.6%, non-Hispanic White: 72.3%, non-Hispanic Black: 10.8%, Asian: 3.2%, non-Hispanic others: 3.1%). Half (55.6%) reported being always engaged by their providers in treatment decision-making. These adults whose providers always vs. never shared decision-making were more likely to be non-Hispanic White (74.8% vs. 56.4%, p<.001), female (62.3% vs. 56%, p<.001), those who held graduate or professional degree (10.9% vs. 6.0%, p<.001), with income level ≥ 400% poverty line (37.4% vs. 22.9%, p<.001), living in Midwest (24.7% vs. 17%, p<.001), had excellent physical health (11.2% vs. 8.1%, p<.001) and mental health status (28.1% vs. 21.5%, p<.001), had a little pain interference (24.9% vs. 16.6, p<.001), and had at least one visit to any CIH modalities (20.2% vs. 16.3%, p<.001). The use of CIH modalities among those who never vs. at least sometimes shared decision-making with their providers was 16.3% vs. 21.7% (p<0.01). CIH users were more likely to have higher income levels and educational attainment. Crude associations indicated higher odds of using CIH modalities among adults who sometimes or usually share decisions (odds ratio (OR): 1.6, 95% CI [1.1-2.3]; 1.7, 95% CI [1.3-2.3], respectively) than those who never did. Yet, after adjusting for individuals’ sociodemographic factors, clinical conditions, and health behaviors, these associations were mitigated, yielding similar CIH use by the level of shared decision-making.

**Conclusions:** Shared decision-making was not associated with using pain-related CIH modalities among adults with CNCP but varied by individuals’ demographic and socioeconomic status. Efforts to promote CIH use should go beyond shared decision-making, and target adults with lower socioeconomic status.

## O25 Deep learning for sleep staging in rodents

### Andrew Smith^1^, Snezana Milosavljevic^2^, Musa Azeem^1^, Courtney Wright^2^, Ana Pocivavsek^2^, Homayoun Valafar^1^

#### ^1^Department of Computer Science and Engineering, College of Engineering and Computing, University of South Carolina, Columbia, SC, USA; ^2^Pharmacology, Physiology, and Neuroscience, School of Medicine Columbia, Columbia, SC, USA

##### **Correspondence:** Homayoun Valafar (homayoun@cse.sc.edu)

*BMC Proceedings 2023*, **17(Suppl 19):**O25

**Background:** Sleep is essential for optimal health^1^, and poor quality of sleep has been associated with cognitive decline, diseases, and disorders. Animal studies are imperative to recapitulate phenotypes and evaluate molecular mechanisms disrupted by sleep loss. Manual annotation of electroencephalography (EEG) recordings of sleep with sleep stages is time-consuming and severely limits efficiency, as it takes an expert about 2 hours to evaluate 24 hours of polysomnographic data. Therefore, developing a reliable and accurate automated method for sleep staging is critical for more efficiently conducting pre-clinical sleep studies and better-connecting sleep loss with health outcomes.

Sleep staging via only single-channel EEG is of particular interest to pre-clinical researchers because it allows for minimally invasive hardware and experimental settings in small animals such as rodents. However, sleep staging via a single-channel EEG rather than multiple channels significantly increases complexity. Several approaches for automated scoring have been established, yet many rely on hand-engineered features that fail to generalize across species, experimental conditions, cohorts, and more, due to inherent bias^2^. Deep learning has recently come into focus in almost all domains because of its success in automatically learning complex nonlinear features from raw data. In this study, we leverage that potential and present a deep-learning approach for sleep scoring in rodents.

**Methods:** For sleep staging, sleep signals are uniformly partitioned into segments of 10s duration called epochs, each of which must be classified into one of three vigilance states: rapid eye movement (REM) sleep, non-REM sleep, or wakefulness. Our model encodes each epoch with a 1-dimensional ResNet^3^ to extract information critical for sleep staging. The temporal transition of this local information is then captured between neighboring epochs via a bidirectional LSTM. Single-channel EEGs are obtained at 500 Hz via a single cortical implant from 16 rodents for 48 hours each and manually annotated by an expert scorer, resulting in 768 hours of raw data. To produce a labeled dataset for supervised learning, we use a sliding window of length 9 with stride 1 (in terms of epochs). The window slides across zero-padded EEG signals such that the label of each window is assigned to the label of the center epoch in that window. We concatenate all windowed recordings, obfuscating rodent identity, and randomly partition windowed samples at the epoch level into training and testing sets (80% and 20%) for optimization and testing. A small portion (5%) of the training set is set aside for validation to avoid overfitting the training set and stop training early. After optimization, the macro f1-score (the harmonic mean between precision and recall) is computed only once on the testing set as a performance metric.

**Results:** On the testing set, our model achieves an f1-score of 91.1%, recall of 91.9%, and precision of 90.4%. These results are meant to describe a within-animal generalization based on the partitioning scheme described above. Our previous model described in [2], trained and validated using the same scheme described here, performs with an f1-score of 84.5%, recall of 84.3%, and precision of 84.7%.

**Discussion:** Deep learning can automatically learn information-rich features from raw single-channel EEG signals that support downstream sleep stage classification performance. Confounds such as leakage of information across training and testing set result from the concatenate-shuffle-and-partition validation scheme used here; therefore, an in-depth evaluation of the model featuring a cross-validation scheme is still required. Further, the generalizability of sleep staging models across species is yet under-studied. We incorporate local epoch transition dynamics information via a bidirectional LSTM; however, the size of the neighborhood (like the receptive field), and even the use of attention-based models remains to be evaluated. The classifier proposed here shows promise as an accurate, reliable, and generalizable automatic method for sleep staging.

**References**

1. Cirelli, C., & Tononi, G. (2008). Is sleep essential?. *PLoS biology*, *6*(8), e216.
2. Smith, A., Anand, H., Milosavljevic, S., Rentschler, K. M., Pocivavsek, A., & Valafar, H. (2021, December). Application of machine learning to sleep stage classification. In *2021 International Conference on Computational Science and Computational Intelligence (CSCI)* (pp. 349-354). IEEE.
3. He, K., Zhang, X., Ren, S., & Sun, J. (2016). Deep residual learning for image recognition. In *Proceedings of the IEEE conference on computer vision and pattern recognition* (pp. 770-778).

## O26 Race and risk: a bioinformatic analysis of Alzheimer’s disease

### Loni S. Taylor, Gregory Gibbs, Bishnu Sarker

#### School of Applied Computational Sciences, Meharry Medical College, Nashville, TN, USA

##### **Correspondence:** Loni S. Taylor (lotaylor23@email.mmc.edu)

*BMC Proceedings 2023*, **17(Suppl 19):**O26

**Objective**: The primary focus of this study is to analyze the differences in alleles and gene data for Apoliprotein E (APOE) of individuals who are of African descent in comparison with the entire population. The hypothesis is set to evaluate whether there will be a significant difference in the onset of the disease by race for alleles and genotypes.

**Method**: This relatability concept is determined by using preprocessing on the data to complete statistical analysis such as computing frequency and distribution tables for the racial groups classified in the data. The design of this study is to use biomarker and participant data of all visits for all participants provided from the National Alzheimer’s Coordinator Center (NACC) to complete an association-based analysis to determine the relatability of age, race, allele and gene distribution in the reported Alzheimer’s data.

**Results**: The population studied had a total 172026 records, 86% of which were white while subject data for African Americans detailed 14%. Results found that overall age did not change the distribution of the gene but may have basis to determine if race was a factor. One of the results of our study showed the differences in race and the APOE gene (figure 1).

Analysis shows that the interquartile range of values for African Americans were statistically similar to Pacific Islanders but greater than whites, supporting the overall argument of a different number of genes but not the median gene allocation. Another statistical analysis that was ran compared the populations’ race data vs the allele distribution. For each race, we reviewed the number of e4 alleles associated. Data frequencies show African Americans generally have more of their population containing less alleles while Caucasians tend to be more numerous overall (figure 2).

Finally, allele and gene data did not reflect the same relationship comparison by race. Within the African American demographic, as the number of alleles increase, the ratio of alleles to subject population were different in comparison to Caucasians.

**Discussion**: Alzheimer’s Disease (AD) is a neurological disorder that affects memory in the brain. It is one of the causes of dementia and inhibits the body’s ability to perform independent living. African Americans are more understudied in this particular disease primarily due to the lack of research in the disease as it exhibits in African Americans. This data and its accompanying results afford the ability to consider utilizing machine learning as a tool for identification of delineating factors. This approach can be used to also import environmental factors for continued investigation. With improvements in this work and others, relationships between APOE associated with onset, APOE distributions amongst races, prediction of causal candidate genes and increased efficiency in early identification of risks and patterns are possible. This will lead to further advancements in remediating the effects this disease has on people of color.

***Key words***: Allele, Alzheimer’s Disease (AD), Apoliprotein E, gene, neurological disorder, machine learning, preprocessing

**References**

1. Ashford JW. APOE genotype effects on Alzheimer's disease onset and epidemiology. J Mol Neurosci. 2004;23(3):157-165. doi:10.1385/JMN:23:3:157
2. Brænne I, Civelek M, Vilne B, et al. Prediction of Causal Candidate Genes in Coronary Artery Disease Loci. Arterioscler Thromb Vasc Biol. 2015;35(10):2207-2217. doi:10.1161/ATVBAHA.115.306108
3. Yu L, Lutz MW, Wilson RS, et al. APOE ε4-TOMM40 '523 haplotypes and the risk of Alzheimer's disease in older Caucasian and African Americans. PLoS One. 2017;12(7):e0180356. Published 2017 Jul 3. doi:10.1371/journal.pone.0180356
4. Mezlini AM, Magdamo C, Merrill E, et al. Characterizing Clinical and Neuropathological Traits of APOE Haplotypes in African Americans and Europeans. J Alzheimers Dis. 2020;78(1):467-477. doi:10.3233/JAD-200228
5. Roses AD, Lutz MW, Saunders AM, et al. African-American TOMM40'523-APOE haplotypes are admixture of West African and Caucasian alleles. Alzheimers Dement. 2014;10(6):592-601.e2. doi:10.1016/j.jalz.2014.06.009
6. Kunkle BW, Schmidt M, Klein HU, et al. Novel Alzheimer Disease Risk Loci and Pathways in African American Individuals Using the African Genome Resources Panel: A Meta-analysis [published correction appears in JAMA Neurol. 2021 May 1;78(5):620]. JAMA Neurol. 2021;78(1):102-113. doi:10.1001/jamaneurol.2020.3536

**Fig. 1 (abstract O26).**
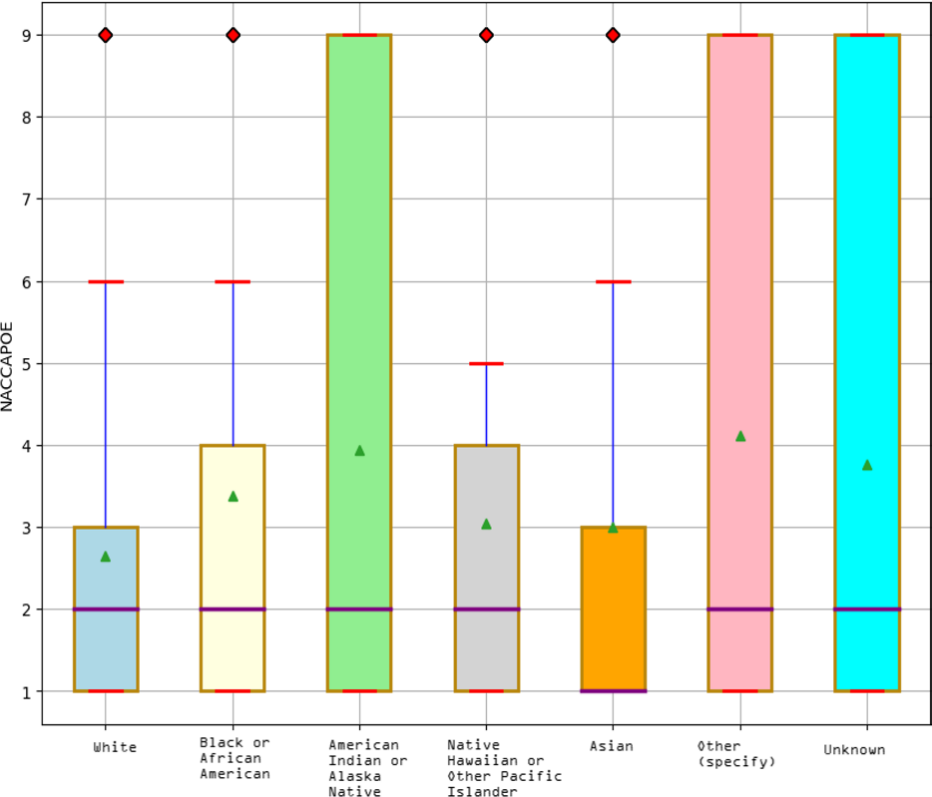
Gene Distribution by race

**Fig. 2 (abstract O26).**
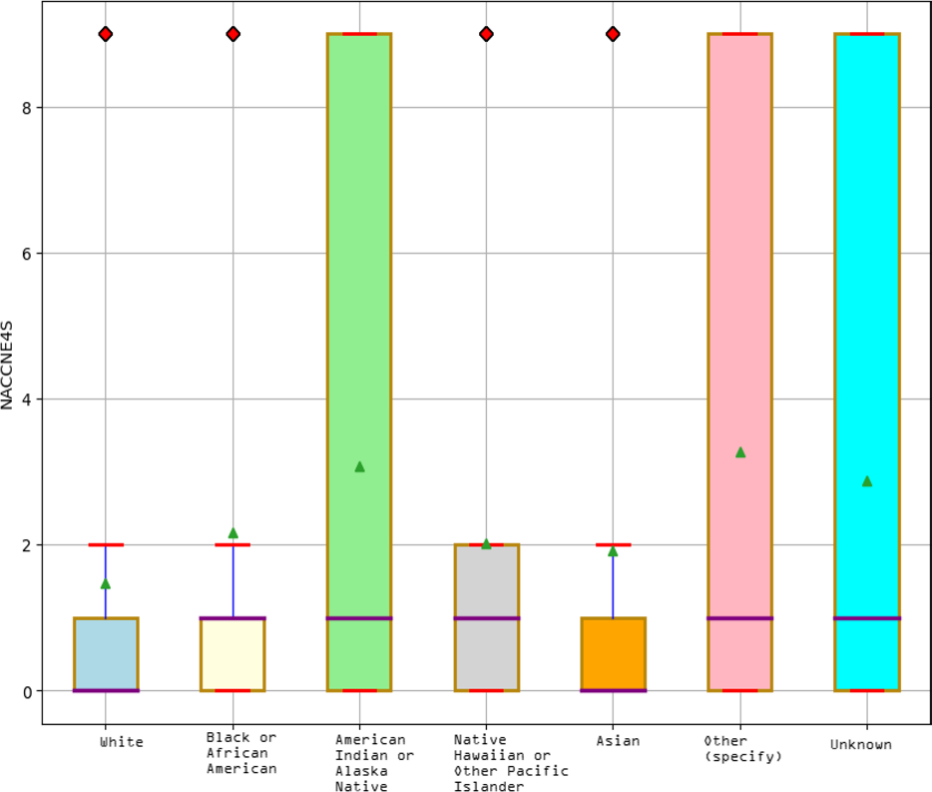
Allele Distribution by race

## O27 Exploring racial disparities in colorectal polyp characteristics at screening colonoscopy using machine learning approaches

### Yuqi Wu^1^, Dezhi Wu^2^, Nabil Natafgi^1^, Chao Cai^3^, Sudha Xirasagar^1^

#### ^1^Arnold School of Public Health, University of South Carolina, Columbia, SC, USA; ^2^Department of Integrated Information Technology, College of Engineering and Computing, University of South Carolina, Columbia, SC, USA; ^3^College of Pharmacy, University of South Carolina, Columbia, SC, USA

##### **Correspondence:** Sudha Xirasagar (sxirasagar@sc.edu)

*BMC Proceedings 2023*, **17(Suppl 19):**O27

**Background:** Despite almost tandem screening colonoscopy rates among Black and White populations for a decade, currently about 60%, Black-White disparities in colorectal cancer (CRC) incidence and mortality persist.^1-4^ The disparities are largely attributed to biologic factors, early polyp initiation and more aggressive progression among Black population. To study racial differences in polyps, a dataset consisting of polyp features of a large cohort was analyzed using traditional regression and showed no racial differences in polyp likelihood and features except for a few attributes on which Whites had more unfavorable status. Because of the large number of polyp features with low frequencies, and the structural challenges of traditional regression to identify features among them in distinguishing two race groups, this study uses machine learning (ML) to address the research question. Identifying specific polyp features with high cancer risk if found disproportionately among Blacks may help accelerate CRC prevention equity by providing detailed polyp status criteria for increased surveillance.

**Methods:** The study will evaluate the performance of multiple supervised ML methods to identify the most important polyp features that distinguish two race groups. Data of 29,425 patients who had screening colonoscopy at an endoscopy center in South Carolina, September 2001 to July 2016 were studied with a total of 48,761 polyps. Of 29,425, 14,636 patients (7,672 Blacks, 6964 Whites) with at least one adenoma or one hyperplastic polyp removed were studied using ML to study polyp differences by race. Seven supervised ML models were evaluated: LR, NB, KNN, SVM, RF, XG-Boost, and AdaBoost. The methods producing the highest performance results were selected, and the most important polyp features that best separated the sample into the two race groups were identified, using Python software.

**Results:** All 3 samples (total, males and females) were randomly split in 80-20 training and testing sets. The testing dataset was used to produce the confusion matrices, showing LR and AdaBoost to be the best performing models (with highest AUROC and accuracy scores). Using these models, the most important polyp features were: 1) Total polyp burden, among top 10 in all 3 samples, and statistically significant among males and females; 2) Presence of hyperplastic polyp in the right colon, among top 10 in full cohort, and statistically significant in traditional regression in all 3 samples; 3) Total polyp burden in the right colon was important in all 3 samples; 4) Hyperplastic polyp burden in the left colon was important in all 3 samples. Direction of association in traditional statistical regression showed that Black population has a more favorable polyp profile.

**Conclusion:** Overall, the study showed that racial differences in polyp profile are at best marginal or favor Black population generally. Traditional regression identified marginal differences between the Black and White populations in the total polyp burden. ML largely confirmed these results and extracted additional information on polyp features that are occasionally present but thought to be associated with higher cancer development potential. Findings indicate that removal of all polyps protects against colorectal cancer, and removal is equally effective by race.

**References**

1. Siegel RL, Miller KD, Goding Sauer A, Fedewa SA, Butterly LF, Anderson JC, Cercek A, Smith RA, Jemal A. Colorectal cancer statistics, 2020. CA: a cancer journal for clinicians. 2020 May;70(3):145-64.
2. Siegel, R., DeSantis, C., & Jemal, A. (2014). Colorectal cancer statistics, 2014. CA: a cancer journal for clinicians, 64(2), 104-117.
3. Siegel, R. L., Miller, K. D., Fedewa, S. A., Ahnen, D. J., Meester, R. G., Barzi, A., & Jemal, A. (2017). Colorectal cancer statistics, 2017. CA: a cancer journal for clinicians, 67(3), 177-193.
4. American Cancer Society (ACS). Cancer Facts and Figures 2022. Retrieved at: https://www.cancer.org/research/cancer-facts-statistics/all-cancer-facts-figures/cancer-facts-figures-2022.html. Accessed in Nov 2022.

## O28 Identifying a group of factors predicting cognitive impairment among older adults

### Longgang Zhao^1^, Yuan Wang^1^, Eric Mishio Bawa^1^, Zichun Meng^1^, Jingkai Wei^1^, Sarah Newman-Norlund^2^, Tushar Trivedi^3^, Hatice Hasturk^4^, Roger D. Newman-Norlund^2^, Julius Fridriksson^2^, Anwar T. Merchant^1^

#### ^1^Department of Epidemiology and Biostatistics, Arnold School of Public Health, University of South Carolina, Columbia, SC, USA; ^2^Communication Sciences and Disorders, Arnold School of Public Health, University of South Carolina, Columbia, SC, USA; ^3^Regional Medical Center Primary Care Stroke, Orangeburg, SC, USA; ^4^Center for Clinical and Translational Research, Forsyth Institute, Boston, MA, USA

##### **Correspondence:** Anwar T. Merchant (merchant@mailbox.sc.edu)

*BMC Proceedings 2023*, **17(Suppl 19):**O28

**Background:** Cognitive impairment has multiple risk factors spanning several domains, but few studies have evaluated risk factor clusters. We aimed to identify naturally occurring clusters of risk factors of poor cognition among middle-aged and older adults and evaluate associations between measures of cognition and these risk factor clusters.

**Methods:** We used data from the National Health and Nutrition Examination Survey (NHANES) III (training dataset, n=4074) and the NHANES 2011-2014 (validation dataset, n=2510). Risk factors were selected based on the literature. We used both traditional logistic models and support vector machine methods to construct a composite score of risk factor clusters. We evaluated associations between the risk score and cognitive performance using the logistic model by estimating odds ratios (OR) and 95% confidence intervals (CI).

**Results:** Using the training dataset, we developed a composite risk score that predicted undiagnosed cognitive decline based on ten selected predictive risk factors including age, waist circumference, healthy eating index, race, education, income, physical activity, diabetes, hypercholesterolemia, and annual visit to dentist. The risk score was significantly associated with poor cognitive performance both in the training dataset (OR _Tertile 3 verse tertile 1_=8.15, 95% CI: 5.36-12.4) and validation dataset (OR _Tertile 3 verse tertile 1_=4.31, 95% CI: 2.62-7.08) (**Table 1**). The area under the receiver operating characteristics curve for the predictive model was 0.74 and 0.77 for crude model and model adjusted for age, sex, and race.

**Conclusion:** The model based on selected risk factors may be used to identify high risk individuals with cognitive impairment.

**Key words:** Cognition; risk factors; prediction; cluster; machine learning

**Table 1 (abstract O28).** Impaired cognition score for cognition performance based on the development and prediction datasets

	Tertiles of impaired cognition scores *		
	Tertile 1	Tertile 2	Tertile 3
**NHANES III (training dataset)**			
Impaired cognition score based on logistic model			
Cases/Controls †	27/1186	91/1123	217/996
Model 1, OR (95% CI) ‡	1.00 (reference)	3.56 (2.30-5.51)	9.57 (6.36-14.4)
Model 2, OR (95% CI) §	1.00 (reference)	3.18 (2.05-4.94)	8.15 (5.36-12.4)
Impaired cognition score based on SVM model			
Cases/Controls	25/1188	82/1132	228/985
Model 1, OR (95% CI)	1.00 (reference)	3.44 (2.18-5.43)	11.0 (7.22-16.8)
Model 2, OR (95% CI)	1.00 (reference)	3.18 (2.01-5.04)	8.77 (5.57-13.8)
**NHANES 2011-2014 (validation dataset)**			
Impaired cognition score based on logistic model			
Cases/Controls	25/811	47/790	113/724
Model 1, OR (95% CI)	1.00 (reference)	1.93 (1.18-3.17)	5.06 (3.25-7.90)
Model 2, OR (95% CI)	1.00 (reference)	1.66 (0.99-2.76)	4.31 (2.62-7.08)
Impaired cognition score based on SVM model			
Cases/Controls	21/815	44/793	120/717
Model 1, OR (95% CI)	1.00 (reference)	2.15 (1.27-3.65)	6.50 (4.04-10.4)
Model 2, OR (95% CI)	1.00 (reference)	1.59 (0.92-2.74)	3.95 (2.34-6.66)

NHANES, National Health and Nutrition Examination Survey; SVM, support vector machine; OR, odds ratio; CI, confidence interval

* The tertiles was based on the distribution of all participants

† Poor cognition performance was defined as the lowest 10% of the distribution of the MMSE score (NHANES III) or a composite score based on the Consortium to Establish a Registry for Alzheimer’s Disease (CERAD), the Animal Fluency (AF), and the Digit Symbol Substitution Test (DSST) tests (NHANES 2011-2014)

‡ Model 1 is the crude model without any adjustments

§ Model 2 adjusted for age, sex, and race

## P1 Trends in prenatal care utilization among women with childbirths in South Carolina during 2016 – 2020: a retrospective study

### Syeda Shehirbano Akhtar, Peiyin Hung

#### Department of Health Services Policy and Management, Arnold School of Public Health, University of South Carolina, Columbia, SC, USA

##### **Correspondence:** Syeda Shehirbano Akhtar (sakhtar@email.sc.edu)

*BMC Proceedings 2023*, **17(Suppl 19):**P1

**Background:** Access to prenatal care is critical to ensure optimal pregnancy outcomes^1^; however, sociodemographic disparities in prenatal care access persist^2^. In the United States, inequities in the PNC utilization become a major challenge given the increasing high-risk pregnancies and medical needs of pregnant woman^3^, especially those living in the Southern states, including South Carolina^4^ where pregnant women are more likely to be in rural communities, to have chronic conditions (e.g., diabetes and hypertension), and to lack access to timely health care. This study aimed to use the most recent data to examine the trends of prenatal care utilization and adequacy in South Carolina over a five-years span and identify maternal factors associated with PNC utilization in South Carolina.

**Methods:** This was a retrospective population-based study of all childbirths from 2016 to 2020 using the birth certificate data from South Carolina Community Assessment Network (SCAN) database. This study has two phases: first, the frequency and percentage of prenatal care utilization and timeliness across all births in South Carolina by year were calculated. We compared prenatal care adequacy (inadequate, intermediate, adequate, and adequate plus), using Kotelchuck index at each birth level. Second, we examined prenatal care utilization variation across different maternal sociodemographic characteristics (such as maternal race/ethnicity, age, marital status etc.) and change in patterns of prenatal care utilization over the five-year period.

**Results:** No prenatal care utilization increased from 0.9 % in 2016 to 1.3 % in 2020 (Figure 1), yielding an absolute 0.44 percentage increase, whereas inadequate prenatal care reduced by 0.12 percentage during the five-year study period. The adequate and adequate plus utilization of prenatal care increased by 0.036 and 0.02 percentages respectively during the study duration. Across all births, the lack of prenatal care and adequacy varied by maternal sociodemographic factors (Figure 2). Black and Hispanic women, teenagers (aged 15-17 years), and unmarried women were more likely to be related with inadequate prenatal care utilization, compared to white married women aged 25-29. Women with inadequate prenatal care, compared to those with adequate prenatal care, were more likely to experience preterm births (32-36 vs. 37+ gestational weeks).

**Conclusion:** The inequities in prenatal care access raise concerns regarding the existing maternal health disparities facing teenage births, racial and ethnic minority populations, and unmarried birthing people in South Carolina. The findings on the slightly decreased prenatal care access but improved adequacy suggest that women who had visited prenatal care providers experienced improved timeliness and access to prenatal care. However, additional efforts should target the birthing people who have not been able to seek prenatal care prior to childbirth. These findings will further supplement the efforts to understand the individual effect of sociodemographic and maternal factors to generate guiding evidence for programmatic and policy initiatives.

**References**

1. Osterman MJK, Martin JA. Timing and Adequacy of Prenatal Care in the United States, 2016.; 2016.
2. Kogan MD, Alexander GR, Jack BW, Allen MC. The Association Between Adequacy of Prenatal Care Utilization and Subsequent Pediatric Care Utilization in the United States.; 1998. http://publications.aap.org/pediatrics/article-pdf/102/1/25/837804/25.pdf
3. Alex FP, Mark T, Wanda B, Sean C. B, Christopher M. Z. Designing your patient’s prenatal care PATH. Contemporary OB/GYN Journal . 2022;67(1).
4. Greg R A, Donald A. Comely. Prenatal Care Utilization: Its Measurement and Relationship to Pregnancy Outcome. *Am J Prev Med*. 1987;3(5):243-253.

**Fig. 1 (abstract P1).**
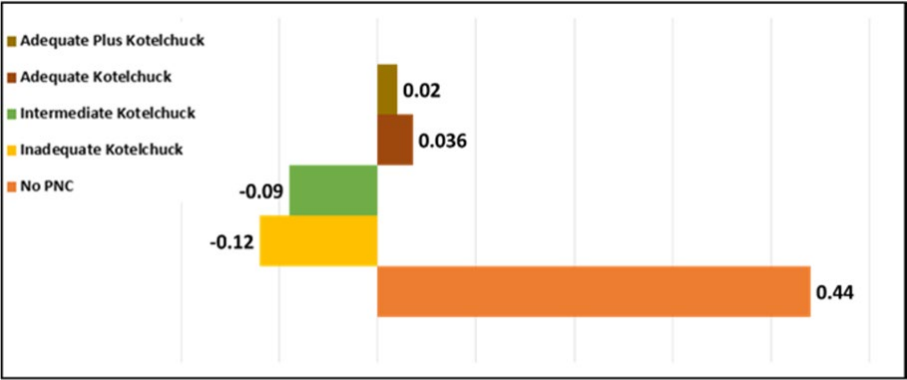
Change in Utilization of PNC (%) in South Carolina for Year 2016-2020

**Fig. 2 (abstract P1).**
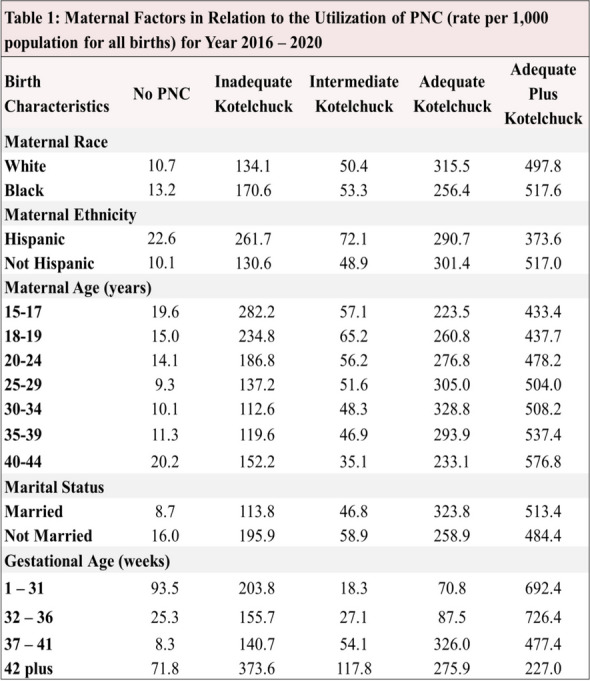
Maternal Factors in Relation to the Utilization of PNC (rate per 1,000 population for all births) for Year 2016-2020

## P2 Building a research database to investigate and promote medical students’ community engagement efforts

### Darby Billing^1^, Elise Kao^1^, Jason Li^2^, Alyssa Guo^2^, Jennifer Grier^1^, Lauren Fowler^2^, Jennifer Springhart^1^

#### ^1^School of Medicine Greenville, University of South Carolina, Greenville, SC, USA; ^2^ Wake Forest University School of Medicine, Winston-Salem, NC, USA

##### **Correspondence:** Jennifer Springhart (jennifer.springhart@prismahealth.org)

*BMC Proceedings 2023*, **17(Suppl 19):**P2

**Abstract**

There has been little evidence-based research in the literature examining the relationship among medical students, community outreach, and volunteerism. Get Connected, also called Campus Connect, is an innovative software that provides organizations, such as medical schools, with the tools to track and record individual volunteer hours. Community engagement in medical schools has been shown to strengthen leadership, promote empathy, cultivate civic and social responsibility, and improve medical school performance. Promoting community engagement in medical schools is difficult due to a variety of factors. Some factors include difficulty in recruiting, scheduling, and managing volunteers to facilitate community service projects. By utilizing a volunteer management software, medical students can sign up, track, and increase awareness of in-person and virtual volunteer opportunities.

Campus Connect by Galaxy Digital is a volunteer management software used by the University of South Carolina School of Medicine Greenville (USC SOMG) to increase medical student volunteer engagement and track our students’ involvement. The school implemented Campus Connect in 2019 and has continued to use it to track students' hours and promote in-person and virtual volunteering opportunities that are posted by the medical school and local community organizations. Students can log hours under 5 sectors: Community Health Improvement Partnerships, Community Medical Services (Free Clinics), School-Based and Educational Initiatives, Social Determinants of Health Initiatives (Food Security, Housing, Economic Stability), and Environmental Initiatives. Furthermore, the logged volunteer hours are all verified by respective organization leadership members. To date, students have logged 8,300.43 hours. Of these hours, 1921.44 were School-Based and Educational Initiatives, 1635.15 were Community Health Improvement Partnerships, 885.50 were Community Medical Services, 468.95 were Social Determinants of Health Initiatives, and 67.00 were Environmental Initiatives. The remaining hours were assigned a sixth category, “Individual”, which includes opportunities that were not previously entered or offered.

The use of a volunteer management software allows community engagement to be tracked systematically to assess community engagement in medical students. Furthermore, it allows volunteer opportunities to be focused on opportunity type (in-person vs. virtual), opportunity focus (Community Health Improvement Partnerships vs. Community Medical Services), and opportunity day and time based on when community engagement is the highest.

One limitation associated with tracking volunteer hours is that students may participate in volunteer opportunities hosted by organizations not associated with the institution or local organizations and choose to not log the hours on the volunteer management software. In the future, additional emphasis can be provided to encourage medical students to log hours on Campus Connect.

The Campus Connect platform will serve as a database and provide the foundation to any future research analyzing the links between medical students at UofSC SOMG and the impacts of community engagement. The use of Campus Connect has gradually become implemented into the UofSC SOMG medical student experience, and over time, the volunteer management software will be able to provide the data necessary to map out the impact of community outreach on future healthcare providers.

**References**

1. Loh AZ, Tan JS, Lee JJ, Koh GC. Voluntary community service in medical school: A qualitative study on student leaders' motivations, experiences, and outcomes. *Med Teach*. 2016;38(7):683-690. doi:10.3109/0142159X.2016.1150985
2. Guidry J, Sarkar A, Little A, Harris T, Brandt M. Using community service to promote awareness of health care-related resources, volunteerism, and teamwork in an incoming medical school class. *Tex Med*. 2013;109(12):e1. Published 2013 Dec 1.
3. Loh AZ, Tan JS, Lee JJ, Koh GC. Voluntary community service in medical school: a qualitative study on obstacles faced by student leaders and potential solutions. *Glob Health Action*. 2015;8:27562. Published 2015 Oct 20. doi:10.3402/gha.v8.27562

## P3 Use of a cascade strategy to infer chronic Hepatitis C follow-up within an infectious disease surveillance system

### Andrew T Broadway^1^, Marya S Barker^2^, Nicholas V. Resciniti^1^

#### ^1^Data Analytics, Management, and Support Section, Division of Acute Disease Epidemiology, South Carolina Department of Health and Environmental Control, Columbia, SC, USA; ^2^Core Surveillance Section, Division of Acute Disease Epidemiology, South Carolina Department of Health and Environmental Control, Columbia, SC, USA

##### **Correspondence:** Andrew Broadway (broadwat@dhec.sc.gov)

*BMC Proceedings 2023*, **17(Suppl 19):**P3

**Introduction:** Infectious disease electronic surveillance systems are generally designed to focus on the management of acute disease cases and outbreaks. These diseases are either curable or self-limiting and long-term follow-up and case management is not necessary. Chronic hepatitis C infections can persist for years^1^, however, and the traditional surveillance systems are not always built to allow ascertainment of the proportion of hepatitis C infections that may have achieved a cure. In 2021 the CDC published *Laboratory-based Hepatitis C Virus Clearance Cascade Program Guidance For Local and State Health Departments*, which was intended to provide guidelines for classifying chronic hepatitis C events based on laboratory records^2^. We began to design a program, using the CDC’s guidelines, to quickly infer a patient’s current hepatitis C clearance status.

**Methods:** We first pulled all chronic hepatitis C laboratory tests from the 2020 calendar year using SQL. We excluded AST, ALT, and bilirubin tests since they are not specific to chronic hepatitis C ^2^. These records were sorted by a unique individual identifier as well as the laboratory specimen collection date. Using SAS 9.4, we deduplicated and categorized these records. Labs with simple text responses indicating positive or negatives were categorized first. Numeric tests were considered positive based on the test parameters, or by standard thresholds where test parameters were not stated. Lastly, genotype tests with a response other than unsatisfactory or insufficient were considered positive. The laboratory records were assigned a binary number based on these results, positive (1) or negative (0). The labs were converted into a binary string for each person. The strings were assessed for patterns potentially indicative of a lack of follow-up (1 with no other tests), clearance (1,0), repeated positive testing (1,1), and reinfections (1,0,1).

**Results:** Out of 13,154 unique individuals testing positive for chronic hepatitis C from January 1^st^, 2020, to December 31^st^, 2020; 9,519 had a positive lab result with no follow-up testing, 3,391 had repeated positive tests, 392 had a pattern indicative of a cure, and 60 had a pattern indicative of reinfection. It should be noted that the last three categories are not mutually exclusive, a person with a string of ‘1101’ would be flagged with all three outcomes, though this sort of pattern was rare.

**Discussion:** We were able to create a system that automatically processes chronic hepatitis C labs into a cure cascade. This can be used to quickly target patients and providers for follow-up based on their

laboratory patterns, as well as output provider and patient phone numbers for easy follow-up, and target high risk groups. In the future we intend to use this system to improve our hepatitis C program. As a caveat, even with highly sensitive tests, it’s possible that some individuals flagged as cleared or reinfected are due to false negatives^3^. Additionally, due to our methodology being contingent on lab testing, we were unable to infer the chronic hepatitis C clearance status of people who did not have follow-up tests that year, which was most of our sample.

**References**

1. Lingala S, Ghany MG. Natural History of Hepatitis C. *Gastroenterol Clin North Am*. 2015;44(4):717-734. doi:10.1016/j.gtc.2015.07.003
2. Centers for Disease Control and Prevention. *Laboratory-based Hepatitis C Virus Clearance Cascade Program Guidance For Local and State Health Departments.* 2021
3. Monaghan TF, Rahman SN, Agudelo CW, et al. Foundational Statistical Principles in Medical Research: Sensitivity, Specificity, Positive Predictive Value, and Negative Predictive Value. *Medicina (Kaunas)*. 2021;57(5):503. Published 2021 May 16. doi:10.3390/medicina57050503

## P4 Developing an ethical framework-guided metric tool for assessing bias in EHR-based big data studies: a research protocol

### E. Graham Caulk (ecaulk@email.sc.edu)

#### Arnold School of Public Health, University of South Carolina, Columbia, SC, USA

*BMC Proceedings 2023*, **17(Suppl 19):**P4

**Abstract**

The current study is concerned with developing an ethical framework-guided metric tool for assessing bias in EHR-based big data studies. At this stage in the research process, our team has been focused on a systematic literature review of different types of bias, methods of measuring these biases, and modes of correcting for them in EHR-based big data studies. The literature has revealed several forms of bias that may be present in different stages of this type of research including: the EHR database development, utilization, de-identification of records, and data aggregation for predictive purposes. We are hopeful that this review will be informative in the development of a research protocol for these types of studies that aims to help elucidate, correct for, and potentially eliminate the biases present in these studies that may lead to ethical concerns for their use as an epidemiological tool.

**References**

1. Gianfrancesco MA, Goldstein ND. A narrative review on the validity of electronic health record-based research in epidemiology. *BMC Med Res Methodol*. 2021;21(1):234. Published 2021 Oct 27. doi:10.1186/s12874-021-01416-5
2. Ninareh Mehrabi, Fred Morstatter, Nripsuta Saxena, Kristina Lerman, and Aram Galstyan. 2021. A Survey on Bias and Fairness in Machine Learning. *ACM Comput. Surv*. 54, 6, Article 115 (July 2022), 35 pages. 10.1145/3457607
3. Juhn YJ, Ryu E, Wi CI, et al. Assessing socioeconomic bias in machine learning algorithms in health care: a case study of the HOUSES index. *J Am Med Inform Assoc.* 2022;29(7):1142-1151. doi:10.1093/jamia/ocac052
4. Kruse CS, Stein A, Thomas H, Kaur H. The use of Electronic Health Records to Support Population Health: A Systematic Review of the Literature*. J Med Syst*. 2018;42(11):214. Published 2018 Sep 29. doi:10.1007/s10916-018-1075-6
5. Reed-Berendt R, Dove ES, Pareek M; UK-REACH Study Collaborative Group. The Ethical Implications of Big Data Research in Public Health: "Big Data Ethics by Design" in the UK-REACH Study. *Ethics Hum Res*. 2022;44(1):2-17. doi:10.1002/eahr.500111

## P5 Disparities of length of hospital stay in fall-related injuries in South Carolina

### Nihan Fila (nfila@email.sc.edu)

#### College of Engineering and Computing, University of South Carolina, Columbia, SC, USA

*BMC Proceedings 2023*, **17(Suppl 19):**P5

**ORCID #**: 0000 0003 4278 1290

**Introduction:** This study aims to analyze the relationship between Length of Stay (LOS) in hospitals and patients' age, gender, race, and admission source due to falls and fall-related injuries in South Carolina (S.C.) in 2007 – 2015. Increasing the life expectancy based on socioeconomic improvements has raised healthcare expenditures where the fall patients' healthcare became a critical issue on family and societal levels^1,2,3^. Since the accidental falls are one of the 10 leading causes of death in S.C. and the U.S., the healthcare policy encompassing falls must be comprehensive for all age groups and should implement improvements in healthcare services^4^.

**Methods:** The Zero Truncated Negative Binomial (ZTNB) model is an appropriate estimation of the LOS because of over-dispersed count data without having a zero value in the response variable (LOS) ^5,6^. Aging and being female are expected to be accelerating factors of LOS^7^. As seen in the Figure below, in terms of age, those aged 75 and over comprise 28.0% of hospitalized patients due to falls, followed by the 45 – 65 age group which makes up 25.3%. The female proportion of hospitalization is 55%. In terms of the admission source, 60.4% of inpatient admissions are through physician referral (PREF), compared to 28.5% for the Emergency Department (ED). The situation is also similar in Europe as “elderly patients make up about 20% of all emergency room visitors in Europe" ^8,9^.

**Results:** The model states that aging is an accelerating factor of LOS when adjusted for other variables. The ZTNB coefficients for the older and middle age (45-65) groups are respectively 2.52 and 3.07 times more likely than young patients in the effect on LOS (Table 1).

On the other hand, the gender of fall inpatients has the opposite impact on LOS than initially predicted and females are 0.92 times less likely than their male counterparts. Hence, it is strongly possible to say that the young male population's behavioral reality might be subject to more fall-related injuries. Furthermore, research on the demographic segmentation of American people between males and females suggests that the gap in the increasing population is in favor of females but getting narrower^10^. As an admissions source group, PREF is 1.325 times more likely to affect LOS than the reference group of ED, even after taking into account the effect of predictors. From a public health perspective, the interrelationship between the referral system and ED admissions in this model is beneficial for policymakers to improve health services by developing social response policies and improving effectiveness for patients of all ages, with special attention to middle-aged and elderly people.

**Conclusion:** This study suggests that as accidental falls are one of the 10 leading causes of death in both S.C. and the U.S., comprehensive health policy covering falls should be differentiated by age groups and the interrelation between referral system and ED admissions and gender. In particular, policies that prevent the decrease in quality of life caused by a disability as a result of falls should be evaluated with environmental and socio-economic factors.

**Acknowledgement**

Special thanks to Neset Hikmet, Ph.D. for his contributions.

**Declaration of Conflicting Interests**

This study is not funded. The author declares that there is no conflict of interest.

**References**

1. Rubenstein, L. Z. (2006). Falls in older people: epidemiology, risk factors and strategies for prevention. Age and ageing, 35(suppl_2), ii37-ii41.
2. World Health Organization, World Health Organization. Ageing, & Life Course Unit. (2008). WHO global report on falls prevention in older age. World Health Organization.
3. Roudsari, B. S., Ebel, B. E., Corso, P. S., Molinari, N. A. M., & Koepsell, T. D. (2005). The acute medical care costs of fall-related injuries among the US older adults. Injury, 36(11), 1316-1322.
4. https://scdhec.gov/sites/default/files/docs/Health/docs/2015_Injury_Report.pdf; Patsy Myers South Carolina Injury Profile - SC DHEC Division of Injury and Violence Prevention; 2015.
5. Hardin, J. W., & Hilbe, J. M. (2015). Regression models for count data from truncated distributions. The Stata Journal, 15(1), 226-246
6. Cruyff, M. J., & Van Der Heijden, P. G. (2008). Point and interval estimation of the population size using a zero-truncated negative binomial regression model. Biometrical Journal: Journal of Mathematical Methods in Biosciences, 50(6), 1035-1050.
7. Stevens, J. A., & Sogolow, E. D. (2005). Gender differences for non-fatal unintentional fall related injuries among older adults. Injury prevention, 11(2), 115-119.
8. Launay, C. P., Kabeshova, A., Lanoé, A., Chabot, J., Levinoff, E. J., & Beauchet, O. (2018). Age effect on the prediction of risk of prolonged length hospital stay in older patients visiting the emergency department: results from a large prospective geriatric cohort study. BMC geriatrics, 18(1), 1-6.
9. Samaras, N., Chevalley, T., Samaras, D., & Gold, G. (2010). Older patients in the emergency department: a review. Annals of emergency medicine, 56(3), 261-269.)
10. Medina, L., Sabo, S., & Vespa, J. (2020). Living longer: Historical and projected life expectancy in the United States, 1960 to 2060. US Department of Commerce, US Census Bureau.

**Fig. 1 (abstract P5).**
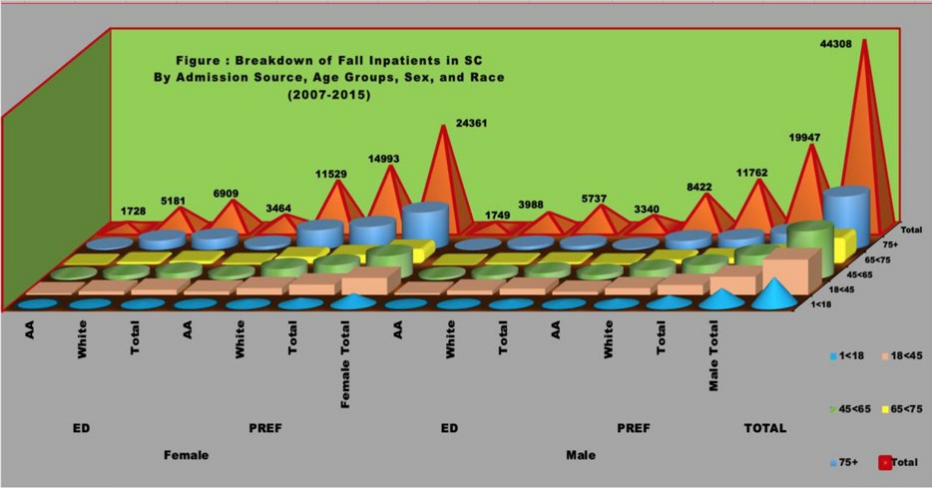
Breakdown of Fall Impatient in SC By Admission Source, Age Groups, Sex, and Race (2007-2015)

**Table 1 (abstract P5).** Zero-Truncated Negative Binomial Model For Fall-Inpatients

	Estimate	Std. Error	Pr(>|z|) c	> exp(est)	2.5%	97.5%
				Estimate		
**ASC3PREF**	***0.281***	***0.018***	< 2e-16 ***	***1.325***	***1.278***	***1.373***
**AgeCat5 early-middle**	***0.896***	***0.037***	< 2e-16 ***	***2.449***	***2.275***	***2.635***
**AgeCat5 middle**	***1.121***	***0.034***	< 2e-16 ***	***3.068***	***2.868***	***3.281***
**AgeCat5 early-older**	***1.079***	***0.036***	< 2e-16 ***	***2.942***	***2.740***	***3.158***
**AgeCat5 older**	***0.924***	***0.034***	< 2e-16 ***	***2.520***	***2.358***	***2.693***
**sexfem1**	***-0.083***	***0.016***	2.98e-07 ***	***0.920***	***0.892***	***0.950***
**Race3- White**	***-0.188***	***0.019***	< 2e-16 ***	***0.828***	***0.797***	***0.860***

Data was obtained from Health Sciences South Carolina's (HSSC) Clinical Data Warehouse (CDW)

Falls are classified as E880-E888 in external causes of unintentional injury within ICD-9, and ICD-10

Missing observations are not more than 5% of 44,308 total observations

## P6 Socio-economic and marital status differences in the uptake of HIV testing in Tanzania: analysis of the 2016-2017 Tanzania HIV impact survey

### Salome-Joelle Gass^1^, Peiyin Hung^1,2^, Jan Ostermann^1,3,4^

#### ^1^Department of Health Services Policy and Management, Arnold School of Public Health, University of South Carolina, Columbia, SC, USA; ^2^Rural and Minority Health Research Center, Columbia, SC, USA; ^3^South Carolina SmartState Center for Healthcare Quality, Arnold School of Public Health, University of South Carolina, Columbia, SC, USA; ^4^Duke Global Health Institute, Duke University, Durham, NC, USA

##### **Correspondence:** Salome-Joelle Gass (sgass@email.sc.edu)

*BMC Proceedings 2023*, **17(Suppl 19):**P6

**Introduction:** Universal HIV testing is a key step toward achieving the UNAIDS goal of ending the AIDS epidemic by 2030. However in 2016/17 only half of people living with HIV (PLWH) knew their positive status.^1^ Globally, marital status and wealth have been identified as key correlates of HIV testing, with unmarried and less wealthy individuals being less likely to test.^2-7^ However, evidence is mixed across geographic contexts, making it difficult to develop targeted HIV testing interventions. This study aims to identify the association of marital status and wealth with HIV testing among youth and adults in Tanzania.

**Methods:** This secondary data analysis used data from a nationally representative sample of 38,680 individuals who participated in the 2016-2017 Tanzania HIV Impact Survey. Weighted logistic regression adjusted for clustering was used to model the association of HIV testing with marital status and wealth, accounting for age, gender, education, and urbanicity of residence. Ever having been tested for HIV and having been tested for HIV in the past 12 months were considered primary outcomes. Post hoc marginal effects estimates were used to calculate probabilities of ever and recent HIV testing by wealth quintile and marital status.

**Results:** In 2016/17, 67.38% of respondents reported having ever been tested for HIV. The highest wealth quintile had 57.3% (Table 1; aOR = 1.573, CI = 1.227, 1.952) higher odds of ever being tested for HIV when compared to the lowest wealth quintile. However, the fourth wealth quintile had the highest odds of ever being tested. Unmarried individuals had the lowest odds of ever being tested (Table 1; aOR = 0.199, 95% CI = 0.173, 0.230). Results were similar when looking at having been tested for HIV in the last 12 months, although a lower difference in odds between unmarried individuals and those married/living together was observed. Interaction analyses indicated that unmarried individuals at all wealth index quintiles had lower probabilities of having ever or recently tested for HIV (Figure 1). Married/living together and widowed/divorced/separated individuals in higher wealth quintiles had a reduction in the probability of testing for HIV, however this difference was not significant (Figure 1).

**Conclusion:** These findings highlight low rates of HIV testing rates among unmarried and less affluent individuals in Tanzania. Previous studies have shown that wealth correlates with higher levels of HIV knowledge, better access to healthcare, and higher rates of HIV testing.^8^ By contrast, individuals in lower wealth brackets face greater logistical and financial obstacles when seeking healthcare.^8^ It is possible that repeat testing as a response to higher risk exposure may be the driver of the lower difference in odds of recent HIV testing between unmarried and married individuals.^9^ Furthermore, a focus of HIV testing programs on the poor, and stigma-related concerns about potential loss of social status, may contribute to lower rates of recent HIV testing among higher wealth indices.^10^ Future HIV testing strategies in Tanzania should target the needs and preferences of unmarried, less wealthy, and wealthier married or previously married individuals.

**References**

1. ICAP. Tanzania HIV Impact Survey (THIS) 2016-2017 Summary Sheet: Preliminary Findings. ICAP 2017.
2. Deynu M, Agyemang K, Anokye N. Factors Associated with HIV Testing among Reproductive Women Aged 15–49 Years in the Gambia: Analysis of the 2019–2020 Gambian Demographic and Health Survey. International Journal of Environmental Research and Public Health. 2022;19(8).
3. Dey NEY, Owusu Ansah K, Norman QA, et al. HIV Testing among sexually active Ghanaians: an examination of the rural-urban correlates. AIDS and Behavior. 2022.
4. Mandiwa C, Namondwe B. Uptake and correlates of HIV testing among men in Malawi: evidence from a national population–based household survey. BMC Health Services Research. 2019;19(1).
5. Siziya S, Muula AS, Rudatsikira E, et al. Correlates of HIV testing among women in Malawi: results from the 2006 Multiple Indicator Cluster Survey. Tropical Medicine & International Health. 2008;13(11):1351-1356.
6. Worku MG, Tesema GA, Teshale AB. Prevalence and associated factors of HIV testing among reproductive-age women in eastern Africa: multilevel analysis of demographic and health surveys. BMC Public Health. 2021;21(1).
7. Ziraba AK, Madise NJ, Kimani JK, et al. Determinants for HIV testing and counselling in Nairobi urban informal settlements. BMC Public Health. 2011;11(1).
8. Jooste S, Mabaso M, Taylor M, et al. Socio-economic differences in the uptake of HIV testing and associated factors in South Africa. BMC Public Health. 2021;21(1).
9. Garcia J, Harichund C, Kunene P, et al. Repeat HIV testing practices in the era of HIV self-testing among adults in KwaZulu-Natal, South Africa. Plos One. 2019;14(2).
10. Deane K, Wamoyi J, Mgunga S, et al. HIV testing attitudes and practices amongst 'wealthy men': qualitative evidence from Tanzania. Culture, Health & Sexuality. 2021;24(9):1215-1229.

**Table 1 (abstract P6).** Weighted logistic regression of association of HIV testing with marital status and wealth index

Covariate		Level	Unadjusted OR	95% CI	P-value	Adjusted OR*	95%CI	P-value
Ever Tested for HIV	Wealth Quintile	Lowest	(base)			(base)		
		Second	1.197	(1.065, 1.346)	**0.004**	1.197	(1.017, 1.408)	**0.032**
		Middle	1.469	(1.300, 1.658)	**<0.0001**	1.435	(1.211, 1.702)	**<0.0001**
		Fourth	1.946	(1.695, 2.234)	**<0.0001**	1.638	(1.354, 1.981)	**<0.0001**
		Highest	2.136	(1.867 2.442)	**<0.0001**	1.573	(1.227, 1.952)	**<0.0001**
	Marital Status	Not married	0.196	(0.181, 0.214)	**<0.0001**	0.199	(0.173, 0.230)	**<0.0001**
		Married/living together	(base)			(base)		
		Widowed/divorced/ separated	0.534	(0.489, 0.582)	**<0.0001**	0.730	(0.648, 0.822)	**<0.0001**
Tested for HIV in Last 12 Months	Wealth Quintile	Lowest	(base)			(base)		
		Second	1.094	(0.957, 1.251)	0.181	1.059	(0.914, 1.223)	0.427
		Middle	1.310	(1.148, 1.494)	**<0.0001**	1.232	(1.074, 1.412)	**0.005**
		Fourth	1.668	(1.444, 1.925)	**<0.0001**	1.408	(1.199 1.653)	**<0.0001**
		Highest	1.860	(1.626, 2.127)	**<0.0001**	1.466	(1.237, 1.737)	**<0.0001**
	Marital Status	Not married	0.470	(0.434, 0.509)	**<0.0001**	0.425	(0.376, 0.481)	**<0.0001**
		Married/living together	(base)			(base)		
		Widowed/divorced/ separated	0.656	(0.599, 0.717)	**<0.0001**	0.866	(0.779, 0.964)	**0.010**

*Adjusted for age, gender, education, and area of residence

**Fig. 1 (abstract P6).**
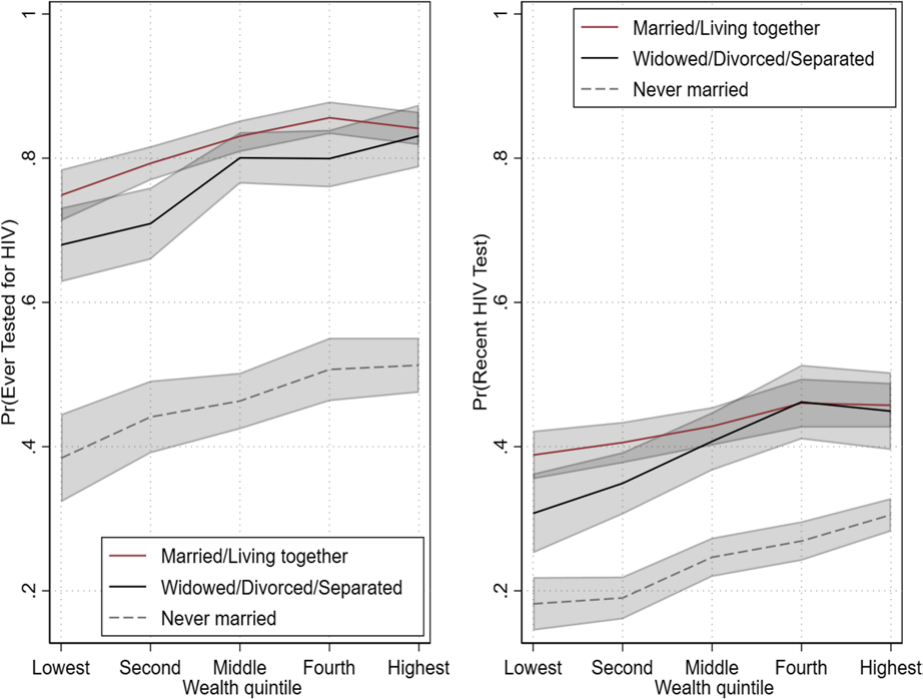
Predicted probability of ever testing for HIV and recently testing for HIV by marital status and wealth quintile

## P7 Hypoglycemia associations with antidiabetic agents: a pharmacovigilance study of the FDA adverse event reporting system (FAERS)

### Julia Geith J, Ryan Gourdine, Chengwen Teng

#### College of Pharmacy, University of South Carolina, Columbia, SC, USA

##### **Correspondence:** Julia Geith (jgeith@email.sc.edu)

*BMC Proceedings 2023*, **17(Suppl 19):**P7

**Objective:** In the United States of America 1 in 10 people have been diagnosed with diabetes.^1^ Prolonged hypoglycemia can lead to severe complications including seizures, coma, and death.^2^ Antidiabetic agents like insulin,^2^ GLP-1 agonists,^3^ DPP-4 inhibitors,^4^ sulfonylureas^4^ and and SGLT-2 inhibitors^5^ have all been known to cause hypoglycemia, but no study has systematically compared hypoglycemia associations between different drug classes. The objective of this study was to evaluate the association between antidiabetic agents and hypoglycemia using the FDA Adverse Event Reporting System.

**Methods:** FAERS reports from January 1, 2004 to December 31, 2021 were included in the study. Reporting odds ratios (RORs) and corresponding 95% confidence intervals (95% CI) for the association between antidiabetic agents and hypoglycemia were calculated. An association was considered to be statistically significant when the lower limit of the 95% CI was greater than 1.0.

**Results:** A total of 14,467,159 reports (including 78,630 hypoglycemia reports) were considered, after inclusion criteria were applied. 30 antidiabetic agents were evaluated, and all of them were significantly associated with hypoglycemia. The top 10 antidiabetic agents with the highest number of hypoglycemia reports were insulin (35,811), metformin (16,610), exenatide (7,046), glimepiride (4,784), glipizide (3,386), glyburide (3,116), sitagliptin (2,900), pioglitazone (2,472), liraglutide (1,658), and dulaglutide (1,615). The top 10 antidiabetic agents with the highest hypoglycemia ROR (95% CI) were pramlintide 34.87 (31.60-38.48), insulin 30.73 (30.29-31.17), acarbose 26.83 (24.62-29.23), repaglinide 26.52 (25.01-28.13), chlorpropamide 23.47 (15.47-35.59), glyburide 22.27 (21.44-23.13), exenatide 19.47 (18.98-19.97), glimepiride 18.84 (18.28-19.43), tolazamide 16.42 (7.58-35.59), and lixisenatide 15.10 (12.81-17.81).

**Discussion:** The antidiabetic agents with the highest association of hypoglycemia found were pramlintide, insulin, acarbose, repaglinide, chlorpropamide, glyburide, exenatide, glimepiride, tolazamide, and lixisenatide. Knowing which classes of antidiabetic drugs are most prone to cause hypoglycemia will help inform clinicians on the most appropriate treatment plan for their patients.

**References**

1. The Facts, Stats, and Impacts of Diabetes (2022) Centers for Disease Control and Prevention. Centers for Disease Control and Prevention. Available at: https://www.cdc.gov/diabetes/library/spotlights/diabetes-facts-stats.html (Accessed: November 16, 2022).
2. Amiel SA. The consequences of hypoglycaemia. *Diabetologia*. 2021;64(5):963-970. doi:10.1007/s00125-020-05366-3
3. Danowitz M, De Leon DD. The Role of GLP-1 Signaling in Hypoglycemia due to Hyperinsulinism. *Front Endocrinol (Lausanne)*. 2022;13:863184. Published 2022 Mar 24. doi:10.3389/fendo.2022.863184
4. Deacon CF, Lebovitz HE. Comparative review of dipeptidyl peptidase-4 inhibitors and sulphonylureas. *Diabetes Obes Metab*. 2016;18(4):333-347. doi:10.1111/dom.12610
5. Li CX, Liang S, Gao L, Liu H. Cardiovascular outcomes associated with SGLT-2 inhibitors versus other glucose-lowering drugs in patients with type 2 diabetes: A real-world systematic review and meta-analysis. *PLoS One*. 2021;16(2):e0244689. Published 2021 Feb 19. doi:10.1371/journal.pone.0244689

## P8 XHealth: data science prerequisites to harnassing big data in healthcare: vectors over scalars

### Stephen C. Lloyd (Stephenclloyd@gmail.com)

#### University of South Carolina, Columbia, SC, USA

*BMC Proceedings 2023*, **17(Suppl 19):**P8

**Abstract**

Artificial Intelligence (AI), according to popular wisdom, is the panacea for the American economy. Healthcare is no exception. The power of AI grew immensely with the advent of machine learning and experienced an additional leap with deep learning. The casual observer of AI has little appreciation for the impact of the transition from algorithmic learning to machine and deep learning. The power to incorporate complex interrelationships without human input trancends rational explaination. Suffice it to say, delivering on the promise of medical informatics impacting the quality or costs of healthcare is dependant on providing the accurately labeled “Big Data.” Most physicians are slaves to the keyboard and transcription systems. They are deluded into the belief that there is a vast repository of valuable clinical data already available to feed the hungry AI beast.

Truth be known, all the dictation and physician cum clerk-typist data entry is valueless in the context of Big Data for healthcare. What is missing? How do we remedy the shortcomings? We have developed a data model that remedies the deficiencies. Conceptually, a patient must be considered a point in health-space. A collection of disease-based vectors, not scalars. Without accurate criteria for diagnosis, information to calculate disease severity, and introducing the notion of the ability to predict outcomes, we will remain stuck with dumb EHRs that intrude on, rather than enhance the patient-physician relationship. Add to this the ability to closely monitor quality of care (both diagnosis and procedural) and the dream of consistant quality, efficient compassionate care can be realized. We describe a system using standardized vocabulary and intercommunication constructs of SNOMED-CT (Standardized NOmenclature for MEDicine, Common Terminology), LOINC (Logical Observation Identifiers Names and Codes), ICD-10, NDC, HL7, and FHIR to capture all relevant data at the point-of-care. The system checks to see if any important data items have been overlooked, BEFORE the patient exits. The monumental problem of missing data will evaporate. Precision medicine is a realistic goal. Costs will drop dramatically (estimated more than half). Access through Telehealth to a personal PCP is assured. The PCPs role will be dramatically enhanced (as will compensation). AI is already capable of analyzing images, so the ability to monitor procedural services (surgery, colonoscopy, biopsies, imaging, and laboratory surviellance) are within our grasp.

One caveat: such a system will require an order higher in magnitude of data entry by the primary care team. The beleagured PCP front line practitioners will balk. We need chatbots (like ChatGPT), fed by the patient and a host of medical assistants, closely guided by the AI engine. By the time the patient comes face-to-face with the provider, the diagnosis will be postulated and the plans for interventions (additional tests, medications, therapies, and monitoring) roughed out. The patient can re-play the video-recorded portions at their leisure. Patient education will consume a far larger portion of the PCP’s efforts. Double the quality for half the cost. In such partnerships, the savings will exceed $2 trillion, all because we are finally able to create the Big Data trove support deep learning for primary healthcare delivery.

## P9 Identify factors associated with emergency department visit rate at ZCTA level

### Zichun Meng^1,2^, Songyuan Deng^1^, Kevin Bennett^1,3^

#### ^1^Center for Rural and Primary Healthcare, Columbia, SC, USA; ^2^Department of Epidemiology and Biostatistics, Arnold School of Public Health, University of South Carolina, Columbia, SC, USA; ^3^Department of Family and Preventive Medicine, School of Medicine Columbia, University of South Carolina, Columbia, SC, USA

##### **Correspondence:** Zichun Meng (zichunm@email.sc.edu)

*BMC Proceedings 2023*, **17(Suppl 19):**P9

**Study Objective:** Emergency Department (ED) visit rates in South Carolina (SC) present a significant variance, with a considerable 34% of residents inhabiting rural areas where medical resources are often insufficient. Our study aims to quantify the association between factors such as chronic disease prevalence, socio-economic indicators, health behaviors, and the rate of ED visits. This data can help identify ED hotspots and inform the efficient allocation of limited resources.

**Methods:** Data on Emergency Room (ER) visits and population numbers for 2019 were extracted at the ZIP Code Tabulation Areas (ZCTA) level from the South Carolina Revenue and Fiscal Affairs Office (RFA) and the American Community Survey (ACS). The Behavioral Risk Factor Surveillance System (BRFSS) supplied prevalence data on chronic diseases (e.g., depression, arthritis, asthma, hypertension, diabetes) and health-related behaviors (e.g., insurance coverage, exercise, binge drinking, smoking, annual physical exams). A multivariable regression model was utilized to quantify the association between ED visit rate and chronic diseases.

**Results:** Across 143 ZCTAs, the average ED visit rate is 453 per 1000 people. The lowest ED visit rate is in Fort Mill (ZCTA 29707) at 16 per 1000, while the highest is in Walterboro (ZCTA 29488) at 928 per 1000. Significant associations were observed between ED visit rates and low education levels (758.6, 95%CI [352.62, 1164.65], p-value <0.01 ), high tobacco use (1131.0, 95%CI [552.03, 1709.99], p-value <0.01), prevalent diabetes (1012.0, 95%CI [347.85, 1676.24], p-value 0.03), high HIV testing rates (610.8, 95%CI [324.13, 897.54], p-value <0.01), and low unemployment rates (570.0, 95%CI [271.99, 867.99], p-value <0.01).

**Conclusion:** Our study suggests that areas with high HIV or Diabetes prevalence require increased ED resource support. In the effort to reduce ED visit rates—which often reflect residents' health status—controlling HIV and diabetes may have a direct impact. However, long-term strategies such as promoting higher education, encouraging employment, and implementing anti-smoking campaigns may also play a significant, albeit more gradual, role in improving public health.

## P10 *“You Want to Use My Data?!?*”: how can patient engagement and outreach enhance big data analytics?

### Ariana Mitcham^1,2^, Conor O’Boyle^2,3^, Ginny Cartee^2^, Katie Parris^2^, Ann Blair Kennedy^2,3,4^, Nabil Natafgi^1,2^

#### ^1^Arnold School of Public Health, University of South Carolina, Columbia, SC, USA; ^2^University of South Carolina Patient Engagement Studio, Columbia, SC, USA; ^3^School of Medicine Greenville, University of South Carolina, Greenville, SC, USA; ^4^Prisma Health Family Medicine, Greenville, SC, USA

##### **Correspondence:** Nabil Natafgi (nnatafgi@mailbox.sc.edu)

*BMC Proceedings 2023*, **17(Suppl 19):**P10

**Issue:** Clinicians, policymakers, and researchers are increasingly more focused on providing patient-centered care. The Patient-Centered Outcomes Research Institute (PCORI) was authorized by Congress in 2010 as part of the ACA to promote patient-centered outcomes research that address healthcare challenges confronting American families every day, including cancer, diabetes, maternal mortality, opioid addiction, mental health, and equitable access to care, among many others. PCORI funds over $400 million in research projects that meaningfully involve and engage patients and stakeholders as equal partners in the research process.

There are several opportunities where patient engagement in research can guide and improve big data research to better healthcare outcomes. Patients and caregivers can propose novel research questions that are meaningful to their conditions, identify health outcomes that are important to them, and inform how and when their health data can (or cannot) be used in research. Patients can provide input and co-develop research with scientists in a variety of settings of big data analytics.

**Program:** The goal of the University of South Carolina (USC) Patient Engagement Studio (PES) is to revolutionize science to serve the patient by meaningfully integrating the patient and community voices in all stages of research from research question development to dissemination and implementation. It serves as a resource for researchers and clinicians from USC and across the nation by offering a structured opportunity for patients, community stakeholders, physicians, and academic researchers to collaborate in the planning, conducting, and dissemination of results of research projects and health system innovations.

The PES has various condition-specific and disease-agnostic panels that reflect the rich experiences of individuals with lived health conditions. The diversity of the PES panels provides a unique perspective to encourage engagement, while effectively and efficiently communicating data on respectful and culturally competent research. Patients and caregivers in the PES are involved in various team-building activities and training sessions on understanding various research terms and methodologies to facilitate their interaction with researchers and clinicians. Those patients/caregivers are referred to as Patient Experts as they are experts in living with the health condition (e.g. Long COVID or diabetes).

Since its inception in 2016, the PES has trained more than 100 patients and partnered with over 350 researchers associated with nearly 50 institutions nationwide. Specifically, the PES has worked with researchers seeking feedback on projects that include social media, EHR extractions, genomics, geospatial components, AI, and health information technology.

**Lessons Learned:** Higher levels of engagement take increasingly higher resources and patient feedback has typically not been seen as a part of big data research (Figure1-adapted from Manafò et al. 2018). However, the PES has learned best practices for providing feedback to research teams from research question formulation through results dissemination. The PES has recognized the next steps for further incorporating patient feedback into big data projects including advising big data researchers on the importance of patient feedback for these types of projects. The most efficient avenue for PES expansion will be identifying big data researchers who are champions of co-developing projects with patient engagement.

**Reference**

1. Manafò E, Petermann L, Vandall-Walker V, Mason-Lai P. Patient and public engagement in priority setting: A systematic rapid review of the literature. *PLOS ONE*. 2018;13(3). doi:10.1371/journal.pone.0193579

**Fig. 1 (abstract P10).**
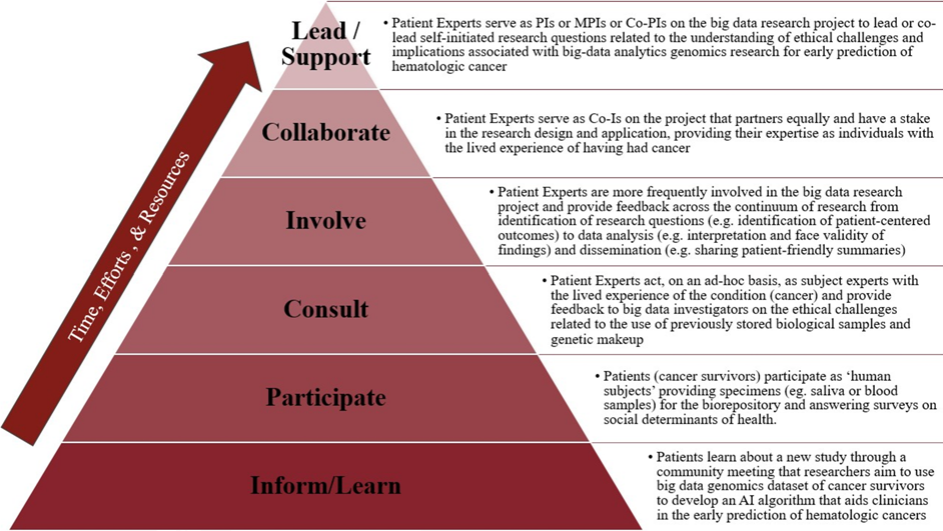
PES Levels of Engagement with Big Data Research

## P11 Annual compliance with colorectal cancer screening in medically underserved, uninsured South Carolinians

### Omar Mushtaq^1^, Lisa Scott^2^, Tracie Lewis^2^, Becky Eaddy^2^, Frank Berger^2^, Troy Herter^2^, Annie Thibault^2^

#### ^1^Department of Biological Sciences, College of Arts and Sciences, University of South Carolina, Columbia, SC, USA; ^2^University of South Carolina, Colorectal Cancer Prevention Network, Columbia, SC, USA

##### **Correspondence:** Omar Mushtaq (omushtaq@email.sc.edu)

*BMC Proceedings 2023*, **17(Suppl 19):**P11

**Abstract**

This study aimed to examine annual compliance of Fecal Immunochemical Test (FIT) among Colorectal Cancer Prevention Network (CCPN) qualified patients who tested negative in previous stool-based screening. Fecal Immunochemical Test (FIT) is a stool based colorectal cancer screening that measures the amount of hemoglobin in the stool sample (NCCN, 2022). For average risk patients, annual participation in FIT screening provides a noninvasive nature and low cost screening option. This said, compliance with recurring annual screening is crucial and can be a struggle for medically underserved and uninsured patients; the specific population for this study.

We reviewed data from FIT test results collected between the period of 2017 to 2020. Specifically, we looked at patients with FIT negative results to see how many completed their FIT test the following year (as per recommendations). Subsequent FIT compliance results are as follows: 3 out of 29 patients screened in 2017 returned for their annual FIT screening in 2018, with an annual FIT compliance rate of 10.34% (Figure 1). Similarly, 5 out of 44 patients screened in 2018 returned in 2019, 19 out of 159 patients screened in 2019 returned in 2020, and 34 out of 175 patients returned for their annual FIT screening in 2021. Annual compliance for fiscal year groups 2017, 2018, and 2019 were similar. However, patients that had their inaugural screening in fiscal year 2020 showed a 7.48% increase in compliance with annual FIT as compared to those that started in the previous year. This increase is statically significant with an odds ratio of 1.63, a Z value of 1.87, and a p-value of 0.030. In 2020 the CCPN implemented new process improvement strategies that centered around virtual patient navigation. This virtual process included video conferencing, mailed-in FIT, virtual educational resources, and instructional videos. Additionally, we began sending out reminders stating, “It’s time for your annual FIT.” The combination of these two factors may have contributed to the observed increase in patient compliance. We will monitor patient compliance to determine if increase in compliance continues. If compliance to annual FIT remains low, we may consider FIT screening to be less than effective in low-income uninsured individuals.

**Fig. 1 (abstract P11).**
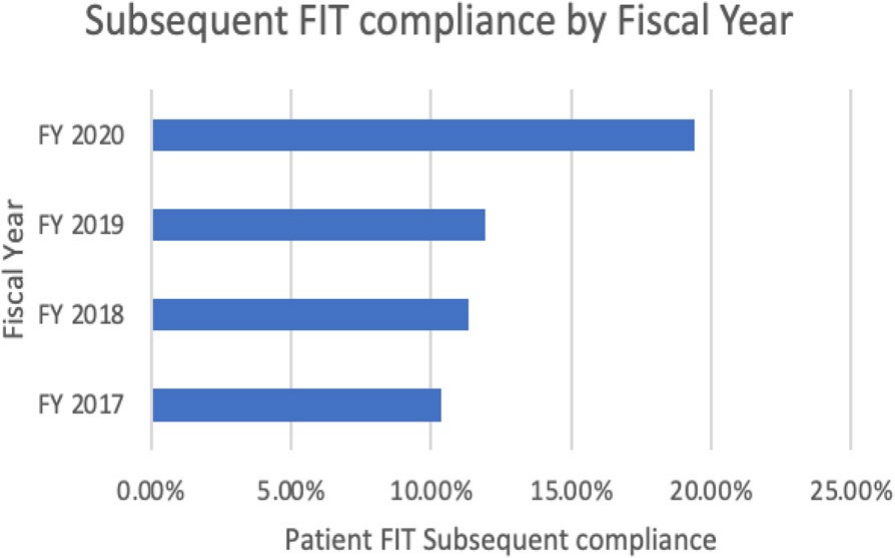
Subsequent FIT compliance by Fiscal Year

## P12 Exploring the social determinants of health in 29203

### Catherine O’Leary^1^, Mark E. Humphrey^2^

#### ^1^School of Medicine Greenville, University of South Carolina, Greenville, SC, USA; ^2^School of Medicine Columbia, University of South Carolina, Columbia, SC, USA

##### **Correspondence:** Catherine O’Leary (csoleary@email.sc.edu)

*BMC Proceedings 2023*, **17(Suppl 19):**P12

**Introduction:** Efforts to improve health equity should include informing healthcare providers about the specific needs of the unique patient population which they serve. Improving the understanding of the social determinants of health (SDOH) of the specific community that physicians are serving allows for targeted interventions based on population needs and prioritization of community partnerships.

**Methods:** In this study, responses to five specific SDOH screening questions were extracted from Epic for the 8206 patients who visited Prisma Health Family Medicine Center (FMC) at Colonial Drive from March 2021 through May 2022. These responses were examined to determine the most common barriers to health faced by the FMC patient population. The responses from patients residing in the 29203-zip code were compared to that of the rest of the FMC patient population for appreciable differences in SDOH.

**Results:** For all patients, regardless of zip code, financial resource strain was the most common social determinant of health barrier, followed by food insecurity. Reported financial resource strain among patients residing in 29203 was higher than non-29203 FMC patients. However, food insecurity, housing insecurity, social connectivity, and transportation needs were roughly uniform across the population regardless of zip-code.

**Discussion:** FMC providers can benefit from the knowledge that a significant portion of their patient population may face financial instability and food insecurity, both of which can greatly impact patient and population health.

## P13 Tackling healthcare access by simplifying access to actionable data

### Samantha Renaud^1^, Qian Huang^2^, Songyuan Deng^1^, Samantha Slinkard-Barnum^1^, Kevin J Bennett^1^

#### ^1^School of Medicine Columbia, University of South Carolina, Columbia, SC, USA; ^2^University of South Carolina, Columbia, SC, USA

##### **Correspondence:** Samantha Renaud (Samantha.Renaud@uscmed.sc.edu)

*BMC Proceedings 2023*, **17(Suppl 19):**P13

**Study Objectives**: Addressing healthcare access issues is a complex problem that requires buy-in from individuals and organizations with varying understanding of technical data. This project was designed to combine relevant sources of information into easy-to-understand indexed scores to tackle high priorities healthcare access issues in South Carolina. The development of placement indices for providers of primary (PCP) and obstetric/gynecological (OB) healthcare are presented.

**Method**: Data, including provider license information, inpatient (IP) and emergency department (ED) information, and healthcare facilities were sourced from various South Carolina based agencies. Additional population information, such as population estimates, number of women aged 15-50, number of births, were collected from the American Community Survey.

The PCP placement score was estimated using provider density, facility density, IP and ED visit rates, and travel distance. The OBGYN placement score was estimated using provider density, facility density, percent of the population that are women of childbearing age, birth rate, and travel distance. Scores were calculated at the state, county, ZCTA, and census track then standardized using mean and standard deviation and ranged from 0-100 (lowest to highest needs). The final index was weighed by the rural population percentage.

**Results**: Index scores were mapped at the ZCTA level and uploaded to the SC Rural Healthcare Resource Dashboard for public access (see figure 1). Results indicate overlap of need for both types of providers. For example, Mountain Rest area (29664) in the northwest border of our state had a high PCP (30.85) and OB (69.52) score. Other areas, such as Trenton (29847), which is northeast of Augusta, has a high OB (60.55) score but a lower PCP (27.29) score. Meanwhile, areas such as Columbia and Greenville have low needs for both types of providers.

**Discussion**: By combining multiple types of data into a single index, these maps are a quick and easy way for individuals and organization to identify areas of greatest need. The publicly available maps can be leveraged to create incentive programs that will drive providers to practice in areas of higher needs. Subsequent hot spot analysis can be conducted to accentuate where placement would result in the greatest impact on access. The results of these Indices also highlight the disparities in healthcare access for rural South Carolinians.

**Fig. 1 (abstract P13).**
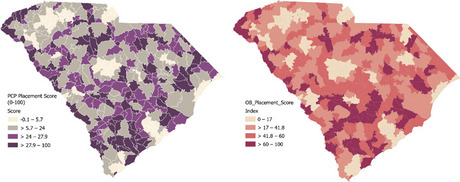
Maps of SC ZCTA PCP and OB Placement Scores. Higher scores are indicated with a darker color and represent a greater need. From SC Rural Healthcare Resource Dashboard (SCRuralHealth.org)

## P14 Influenza vaccination and climate change in the American South

### Ebony Allen Toussaint (eatoussaint@sc.edu)

#### University of South Carolina, Columbia, SC, USA

*BMC Proceedings 2023*, **17(Suppl 19):**P14

**Objective:** In this article, we measure the effect of geography on influenza vaccination uptake in the United States. The implications of climate change are discussed.

**Methods:** We estimated logistic regression models from adult responses to the Behavioral Risk Factor Surveillance System in the continental United States, Alaska, and Hawaii for 2019-2021 using the Andersen Behavioral Model of Health Services Use.

**Results:** Among respondents in the pooled sample population, 44% received an influenza vaccination. Of those vaccinations, 16% occurred among adults living in the American South compared with 29% elsewhere in the United States. Adults in urban counties of the American South were more likely to have received an influenza vaccination; this difference was statistically significant. When controlling for enabling factors, residing in the American South became insignificant (p-value=0.67), while residing in a rural county remained significant (p-value=0.00). Having health insurance only slightly decreased the odds of influenza vaccination, yet those who reported having a usual source of care were three times as likely to receive an influenza vaccination. When estimating the full model, accounting for geographic and sociopolitical differences, adults residing in rural counties were the least likely to receive an influenza vaccination and those with a usual source of care were two times as likely to receive an influenza vaccination. Individuals with one of the top five chronic health conditions, older adults, and women were also more likely to have received an influenza vaccination.

**Discussion:** Having a usual source of care increased the odds of receiving an influenza vaccination, as most influenza vaccinations were received at a doctor’s office followed by a supermarket or drugstore. Additionally, older adults and individuals with chronic health conditions are more likely to visit these places. As the American South continues to grapple with the effects of climate change, rural areas will be most impacted. While influenza remains a top cause of mortality in the United States, health policy related to vaccination needs to consider geographic differences in access to healthcare.

## P15 Multitask learning for South Carolina’s prenatal maternal care

### Edward Tsien^1^, Dezhi Wu^1^, Ana Lòpez-De Fede^2^

#### ^1^College of Engineering and Computing, University of South Carolina, Columbia, SC, USA; ^2^The Institute of Families in Society, University of South Carolina, Columbia, SC, USA

##### **Correspondence:** Dezhi Wu (dezhiwu@cec.sc.edu)

*BMC Proceedings 2023*, **17(Suppl 19):**P15

**Study Objectives**: Many states in the South rank poorly for *preterm birth, low birthweight, and severe maternal morbidity*. In South Carolina, the Department of Health and Human Services (SC DHHS) has been making great efforts to implement innovative approaches to reduce maternal morbidity and improve prenatal outcomes with targeted interventions^1^. However, these interventions require accurate criteria for determining which potential mothers are at risk^2^. Recent literature has demonstrated the ability of deep learning to predict adverse outcomes such as mental health^3^, chronic health conditions^4^, and maternal morbidity^5^, but these studies do not have the wealth and depth of data to justify using deep learning. Multitask learning (MTL)^6^ is a form of deep learning that leverages related targets within a dataset to build a single model that has a shared knowledge base^7^. These shared insights serve to stabilize and regularize the model, as well as reduce overfitting especially in the context of electronic health records.^8^ In this study we obtained a large and rich maternal health dataset to train a MTL model and to demonstrate the effectiveness of certain improvements to the MTL model that we are developing.

**Method**: Our provided dataset had 271,233 maternal delivery records with 95 features collected from 2015 to 2021. We further augmented the data with Social Determinants of Health and Social Vulnerability Index statistics from the CDC based on maternal location. We trained two MTL models: one for three morbidity related tasks and one for six long-term chronic tasks. Morbidity tasks were severe maternal morbidity (SMM), hemorrhage, and eclampsia; chronic tasks were cardiovascular disease, hypertension, diabetes, obesity, mental, and substance use. Since most of these are rare conditions, we had to mitigate class imbalance via random undersampling and model bias initializations. We created a new method for balancing the training of targets called Task-Adaptive Loss (TAL) gradient strategy. We evaluated each task with F-score and AUC-ROC.

**Results**: Performance-per-task of the MTL models is greater than single-task DNN and ML models on most tasks, except for two targets (Table 1). The Substance Abuse task had comparatively low performance across the board due to a lack of features that related to it. The hemorrhage classification performance is similar between all models (within 2% of each other) suggesting that the underlying prediction task shares few insights or similarities with the other two tasks in the morbidity group.

**Conclusion**: The model demonstrates the possibility of creating a tool pulling in data from several different domains to help identify the characteristics of obstetric patients of highest risk for poor maternal outcomes. In addition, we will be further refining our improvements (TAL) to apply to multitask learning with particular relevance to health informatics. In doing so, our study results should be informative to SC public health policy makers and to clinicians seeking to assess patient risk.

**References**

1. Lisa F. Waddell, B.Y.M., Michael G. Smith, Lucy H. Gibson, Breana N. Lipscomb, Daniela Nitcheva: ‘Healthy Mothers, Healthy Babies:South Carolina’s Plan To Reduce Infant Mortality & Premature Births’, in Editor (Ed.)^(Eds.): ‘Book Healthy Mothers, Healthy Babies:South Carolina’s Plan To Reduce Infant Mortality & Premature Births’ (South Carolina Dept of Health and Environmental Control, 2013, edn.), pp.
2. Gareau, S., Lòpez-De Fede, A., Loudermilk, B.L., Cummings, T.H., Hardin, J.W., Picklesimer, A.H., Crouch, E., and Covington-Kolb, S.: ‘Group Prenatal Care Results in Medicaid Savings with Better Outcomes: A Propensity Score Analysis of CenteringPregnancy Participation in South Carolina’, Maternal and Child Health Journal, 2016, 20, (7), pp. 1384-1393
3. Andersson, S., Bathula, D.R., Iliadis, S.I., Walter, M., and Skalkidou, A.: ‘Predicting women with depressive symptoms postpartum with machine learning methods’, Scientific Reports, 2021, 11, (1)
4. Wu, Y.-T., Zhang, C.-J., Mol, B.W., Kawai, A., Li, C., Chen, L., Wang, Y., Sheng, J.-Z., Fan, J.-X., and Shi, Y.: ‘Early prediction of gestational diabetes mellitus in the Chinese population via advanced machine learning’, The Journal of Clinical Endocrinology & Metabolism, 2021, 106, (3), pp. e1191-e1205
5. Irfan, M., Basuki, S., and Azhar, Y.: ‘Giving more insight for automatic risk prediction during pregnancy with interpretable machine learning’, Bulletin of Electrical Engineering and Informatics, 2021, 10, (3), pp. 1621-1633
6. Caruana, R.: ‘Multitask Learning’ (Springer, 1998. 1998)
7. Abu-Mostafa, Y.: ‘A method for learning from hints’, Advances in Neural Information Processing Systems, 1992, 5
8. Espinosa, C., Becker, M., Marić, I., Wong, R.J., Shaw, G.M., Gaudilliere, B., Aghaeepour, N., and Stevenson, D.K.: ‘Data-Driven Modeling of Pregnancy-Related Complications’, Trends Mol Med, 2021, 27, (8), pp. 762-776

**Table 1 (abstract P15).** Performance of single-task models vs. stock MTL models vs. our MTL+TAL models

Tasks	Single-task models from literature		MTL model		MTL + TAL model	
	AUC-ROC	F-Score	AUC-ROC	F-Score	AUC-ROC	F-Score
**Morbidity:**						
**SMM**	0.7617 XGBoost	0.8071	0.8658	0.7993	**0.8878**	**0.8186**
**Hemorrhage**	**0.8482** **DNN**	0.7897	0.8365	0.8177	0.8450	**0.8285**
**Eclampsia**	0.8110 RF	0.8273	0.8374	0.8350	**0.8495**	**0.8442**
**Chronic:**						
**Cardio. Disease**	0.7113 DT	0.7193	0.7735	0.7408	**0.7951**	**0.7542**
**Hypertension**	0.8195 XGBoost	0.8352	0.8877	0.8561	**0.9124**	**0.9237**
**Diabetes**	0.7327 XGBoost	0.7391	0.8306	0.7985	**0.8425**	**0.8716**
**Obesity**	0.7421 RF	0.7394	0.8527	0.8294	**0.8892**	**0.8853**
**Mental**	0.8664 RF	0.8110	0.8704	0.8535	**0.9039**	**0.8633**
**Substance Use**	**0.7845** **LR**	0.6585	0.5718	**0.7210**	0.7785	0.7109

## P16 AI-based mining of biomedical literature: applications for the drug repurposing

### Aliaksandra Sikirzhytskaya^1^, Ilya Tiagin^2^, Joe Magagnoli^1^, Tammy Cummings^1^, Michael Wyatt^1^, Scott Sutton^1^, Ilya Safro^2^, Michael Shtutman^1^

#### ^1^University of South Carolina, Columbia, SC, USA; ^2^University of Delaware, Newark, DE, USA

##### **Correspondenec:** Michael Shtutman (shtutmanm@cop.sc.edu)

*BMC Proceedings 2023*, **17(Suppl 19):**P16

**Background:** Repurposing approved drugs for new disease indications drastically reduces the drug development down to 6 years or less. Biomedical data and literature are exponentially growing, making it harder for investigators to keep up with, much less analyze and discover hidden connections in these vast datasets. Primary biomedical datasets, such as PubMed, PMC, ClinicalTrials, and others have vast information that can be mined to generate new hypotheses of disease development, progression, as well as novel treatment avenues.

We have developed and employed MOLIERE and AGATHA, AI-based literature mining systems that discover novel interactions that potentially lead to new therapeutic discoveries. MOLIERE/AGATHA are able to handle knowledge networks of 100 billion edges (tested) with a fast query process. AGATHA is a deep-learning hypothesis generation system that can introduce data-driven insights earlier in the discovery process. AGATHA quickly prioritizes plausible term pairs among entity sets through learned ranking criteria, allowing the recommendation of new research directions. AGATHA has been applied to discover new treatments for HIV-associated neurocognitive disorder (HAND) and Substance Use Disorder (SUD). With AGATHA, we generated a list of HAND-associated genes and with corresponding small molecule inhibitors, including FDA-approved drugs. Several drug repurposing candidates were validated in lab models of neuronal damage caused by HIV and SUD. Also, the effects of FDA-approved drugs on diseases different from their indications is analyzed in the Electronic Health Records of the Department of Veteran Affairs (VA) using the VA Informatics and Computing Infrastructure (VINCI). We provide below one successful example of a drug uncovered via AGATHA that was tested for potential HAND risk reduction.

**Methods:** The study evaluated HIV and angiotensin-converting enzyme inhibitors (ACEi) categorized by their ability to cross the blood brain barrier (BBB). Electronic medical records from Oct 1999 through June 2022 were included and evaluated demographic, comorbid, clinical and mortality data. We investigated if there was an immediate risk of the study outcome of dementia while censoring at 1 and 5 years. Differences among the groups were evaluated using t-tests, chi-square tests and adjusted Cox proportional hazards model.

**Results:** Records from 46563 patients were included in the study (16222 BBB ACEi cohort and 30341 nonBBB ACEi cohort). Most patients were black males. The average age was 49 and 45 (p-value<0.05) while the Charlson comorbidity index (CCI) was 0.15 and 0.16 (p-value<0.05) in the BBB ACEi cohort and nonBBB ACEi cohort respectively. When censored at 1 year, the BBB ACEi cohort had fewer patients with dementia (0.76% vs. 1.12%; p-value<0.05), had a higher average time to dementia (162.3 days vs. 130.0 days; p-value<0.05), and had a lower risk of dementia (aHR=0.661; 95% CI=0.523-0.835) compared to the nonBBB ACEi cohort. When censored at 5 years, the BBB ACEi cohort had fewer patients with dementia (2.19% vs. 2.49%; p-value<0.05), had a higher average time to dementia (2.0 years vs. 1.7 years; p-value<0.05), and had a lower risk of dementia (aHR=0.734; 95% CI=0.635-0.849). The results provide new insight into mechanisms of diseases and new treatment options, and validate our novel big data querying approaches.

## P17 A Bayesian spatial scan statistic for normal data

### Laasya Velamakanni, Yuan Wang, Alexander McLain, Stella Self

#### University of South Carolina, Columbia, SC, USA

##### **Correspondence:** Laasya Velamakanni (laasya@email.sc.edu)

*BMC Proceedings 2023*, **17(Suppl 19):**P17

**Abstract**

Scan statistics are used to detect spatial clustering. While they were initially developed to detect regions with an excess of binomial or Poisson events, spatial scan statistics have been extended to detect hotspots in other types of data including continuous data. Spatial scan statistics have also been extended to the Bayesian paradigm for a limited number of data types, including zero-inflated count data and multivariate count data. The Bayesian spatial scan statistic has not been developed for continuous data. Thus, in this work, we develop a Bayesian spatial scan statistic for detecting “areas of clustering” using normally distributed data, where a cluster represents some part of a study area in which the mean value is higher than the rest of the study area.

The Bayesian spatial scan statistic is designed to detect clustering in continuous valued data that has been collected at different spatial locations. We implement a hypothesis test for clustering using the Bayes factor, in which the alternative hypothesis indicates a cluster of observations for which the means are different from the rest of the data. In order to apply our method, we first identify the most likely cluster as the potential cluster for which the likelihood under the alternative hypothesis is maximized. We conduct a simulation study to evaluate the performance of our method under varying sample sizes, cluster sizes, and observation means.

Simulation results consist of the empirical type I error rate for data simulated under the null hypothesis, the empirical power for data simulated under the alternative hypothesis, and the average sensitivity and positive predictive value (PPV) of the test. We observe that the performance of the method gets better as the clustering gets stronger and the sample size increases (the rejection rate increases). Furthermore, the performance of the method improves when we have a large cluster versus a small cluster.

The motivation behind a Bayesian approach includes the ability to incorporate prior information when available and directly calculate posterior probabilities. Comparing our Bayesian spatial scan statistic to the frequentist spatial scan statistic, we observe that the Bayesian statistic does not seem to have an advantage here. If we had historical data, we may have been able to set informative prior distributions which could give more power. We may consider this for future work. Some possible ideas for future work include looking at different priors as well as speeding up the computation time.

## P18 The dose-response associations between physical activity and cognitive function in older Americans in different demographic subgroups

### Fanli Yi^1^, Carlos Avalos^2^, Chelsea Richard^1^, Chih-Hsiang Yang^3^

#### ^1^Department of Epidemiology and Biostatistics, Arnold School of Public Health, University of South Carolina, Columbia, SC, USA; ^2^Division of Population Health Surveillance, Bureau of Maternal and Child Health, Columbia, SC, USA; ^3^Department of Exercise Science/TecHealth, Arnold School of Public Health, University of South Carolina, Columbia, SC, USA

##### **Correspondence:** Fanli Yi (yif@email.sc.edu)

*BMC Proceedings 2023*, **17(Suppl 19):**P18

**Background:** A positive relationship between physical activity (PA) and cognition has been found^1-3^. However, it is less clear whether there is a dose-response association between the effect of PA and cognitive decline or how this association varies among the different demographic groups. This study utilized the device-based physical activity (PA) measures and the performance-based cognitive measures from NHANES 2011-2014 to investigate the dose-response association between PA and cognition among US older adults ≥ 60 years.

**Methods:** The 2011-2014 NHANES survey comprised 2,547 older adults 60 years old or above. The duration and intensity of PA were self-reported and collected using ActiGraphs. The cognitive tests include the Consortium to Establish a Registry for Alzheimer’s Disease Word List Memory Task, Digit Symbol Substitution Test (DSST), and Animal Fluency Test^4-7^. This study used the average daily PA and average peak 30-minute Monitor Independent Movement Summary (MIMS) to predict the test-specific and combined global Z scores for cognition. The peak 30-min MIMS is the average for the mean of peak 30 minutes that had the highest MIMS on each valid wear day. Sample weight-adjusted multivariable linear regression was used to evaluate the association in cognitive function by different PA levels. Quantile regressions were used to compare the associations between PA and cognition in various subgroups.

**Results:** PA and cognitive function differ by gender, age, race, occupation, hypertension status, and BMI (**ps<.05**). There was a positive association between a cognitive function with peak-30 (MIMS/min) (LSMEANS=46.40%-47.99%, *p*=0.03) and vigorous physical activity (LSMEANS=50.07%, *p*=0.01), whereas no overall improvement in cognitive function was seen in daily PA (MIMS/d). Both daily PA and Peak-30 were associated with better cognitive function in people of color, people who are in lower education levels, those in medium socioeconomic status, married, social participants, healthy in weight, sleep, and with diabetes but no other chronic conditions (**ps**<.05, **Table 1**).

With different quantiles of cognition from 10%, 25%, 50%, 75%, to 90%, the increases in cognitive function were attenuated by participants’ peak-30 (MIMS/min). These attenuated associations are especially pronounced in Mexican American and Hispanics, individuals with no occupation, individuals with drinking behaviors, individuals with regular social participation, individuals with healthy weight and longer sleeping hours, and individuals with no depression. However, the magnitude of increases in cognitive function declined as the increase of quantile groups (see **Table 2**).

**Conclusion:** Using national representative data, our findings supported the literature that higher PA represented by the mean Peak-30 (MIMS/min) and daily physical activity (MIMS/d) is associated with better cognitive function. This observation is especially pronounced among older Americans with the poorest cognitive ability.

**References**

1. Zheng P, Pleuss JD, Turner DS, Ducharme SW, Aguiar EJ. Dose–Response Association Between Physical Activity (Daily MIMS, Peak 30-Minute MIMS) and Cognitive Function Among Older Adults: NHANES 2011–2014. *The Journals of Gerontology: Series A*. 2023;78(2):286-291.
2. Tudor-Locke C, Schuna Jr JM, Han H, et al. Step-based physical activity metrics and cardiometabolic risk: NHANES 2005-06. *Medicine and science in sports and exercise*. 2017;49(2):283.
3. Adams B, Fidler K, Demoes N, et al. Cardiometabolic thresholds for peak 30-min cadence and steps/day. *PLoS One*. 2019;14(8):e0219933.
4. Morris JC, Heyman A, Mohs RC, et al. The consortium to establish a registry for Alzheimer's disease (CERAD): I. Clinical and neuropsychological assessment of Alzheimer's disease. *Neurology*. 1989.
5. Fillenbaum GG, van Belle G, Morris JC, et al. Consortium to Establish a Registry for Alzheimer’s Disease (CERAD): the first twenty years. *Alzheimer's & dementia*. 2008;4(2):96-109.
6. Wechsler D. WAIS Manual–Third Edition. Psychological Corporation New York; 1997.
7. Strauss E, Sherman EM, Spreen O. *A compendium of neuropsychological tests: Administration, norms, and commentary*. American chemical society; 2006.

**Table 1 (abstract P18).** The associations of cognitive function and physical activity in subgroups

Demographic characters		Global Z score for cognition	Global Z score for cognition	The coefficient for the trend (P-value)	Global Z score for cognition	Global Z score for cognition	The coefficient for the trend (P-value)
		Increased LSMEANS^1^ (%) P-value^2^	Increased LSMEANS (%) P-value		Increased LSMEANS (%) P-value	Increased LSMEANS (%) P-value	
		Peak-30 level 1 (ref)^**3**^ Vs Peak-30 level 2	Peak-30 level 1 (ref)^**3**^ Vs Peak-30 level 3		Peak-30 level 1 (ref)^**4**^ Vs Peak-30 level 2	Peak-30 level 1 (ref)^**4**^ Vs Peak-30 level 3	
Adjust for all the demographic characters		**46.40 (0.03)**	**47.99 (0.03)**	**0.02(0.02)**	16.67 (0.35)	23.36 (0.18)	0.00001(0.11)
Age	60-69 years old	161.65 (0.25)	145.31(0.36)	0.01(0.28)	138.13 (0.47)	195.92 (0.31)	0.0000075 (0.52)
	70-80 years old	**67.78 (0.004)**	**75.66 (0.002)**	**0.04(0.002)**	27.33 (0.18)	15.90 (0.58)	0.0000308 (0.11)
	Above 80 years old	-11.53 (0.24)	**33.45 (0.004)**	0.006 (0.46)	-15.86 (0.22)	-58.68 (0.0005)	**-**0.0000065 (0.68)
Racial groups	Mexican American	**108.79 (0.11)**	**111.66 (0.001)**	**<.0001**	**452.72 (0.003)**	**286.13 (0.02)**	**0.000057 (0.02)**
	Other Hispanic	**36.87 (<0.0001)**	**38.83 (0.0001)**	**<.0001**	**36.52 (<0.0001)**	**40.48 (<0.0001)**	**0.000053 (<0.0001)**
	Non-Hispanic White	**208.86 (0.02)**	**198.68 (0.04)**	0.016 (0.06)	108.00 (0.58)	153.36 (0.42)	0.000012 (0.21)
	Non-Hispanic Black	**2.36 (0.002)**	**11.85 (<0.0001)**	**0.010 (0.001)**	**10.90 (0.005)**	**11.65 (<0.0001)**	**0.0000088 (0.04)**
	Non-Hispanic Asian	**747.88 (<0.0001)**	**202.57 (0.39)**	0.008 (0.74)	1030.13 (0.03)	644.46 (0.38)	0.000039 (0.39)
Genders	Male	**39.72 (0.033)**	43.15 (0.10)	**0.02 (0.04)**	23.57 (0.22)	9.52 (0.70)	0.000015 (0.34)
	Female	166.21 (0.22)	123.89 (0.42)	0.008(0.41)	42.81 (0.80)	144.47 (0.42)	0.000011 (0.39)
Education	<9^th^ grade	**20.22 (0.01)**	7.88 (0.50)	**0.028(<.0001)**	**-2.86 (0.80)**	**-2.78 (0.88)**	**0.000099 (0.005)**
	9-11th grade	**182.67 (<0.0001)**	**328.52 (<.0001)**	**0.08 (<.0001)**	**40.55 (<0.0001)**	**98.95 (<0.0001)**	**0.000073 (<0.0001)**
	High school	53.64 (0.08)	50.22 (0.22)	**0.03(0.02)**	**37.16 (0.50)**	**75.99 (0.09)**	**0.000047 (0.006)**
	Some college	38.18 (0.61)	48.73 (0.23)	0.003(0.81)	9.61 (0.89)	-25.92 (0.75)	0.0000029 (0.88)
	College or above	54.72 (0.39)	68.70 (0.29)	0.01(0.24)	38.91 (0.38)	28.05 (0.64)	-0.0000016 (0.94)
Occupation	Having occupation	**88.20 (<0.0001)**	**91.89 (<0.0001)**	**0.02(0.04)**	59.79 (0.07)	62.64 (0.06)	0.000029 (0.07)
	Not having occupation	29.38 (0.12)	29.55 (0.26)	0.014 (0.15)	-10.76 (0.63)	-17.27 (0.56)	0.000012 (0.42)
Smoking status	Smoking	49.32 (0.31)	37.40 (0.09)	**0.02 (0.01)**	16.98 (0.30)	32.97 (0.11)	0.000012 (0.23)
	Non-smoking	129.10 (0.07)	75.72 (0.44)	0.008 (0.49)	18.52 (0.83)	35.56 (0.75)	0.000012 (0.50)
Alcohol drinking	Drinking	-10.05 (0.70)	34.12 (0.36)	0.02(0.20)	**7.40 (0.67)**	**43.22 (0.13)**	**0.000034 (0.04)**
	Non-drinking	**63.50 (0.005)**	45.42 (0.07)	**0.02 (0.03)**	30.51 (0.17)	12.48 (0.56)	0.0000096 (0.36)
Social participation	No difficulty	**68.56 (0.03)**	**84.28 (0.01)**	**0.02(0.005)**	**39.36 (0.30)**	**59.07 (0.10)**	**0.000021 (0.01)**
	Some difficulty	**43.16 (0.001)**	**-28.27 (<.0001)**	-0.004(0.08)	7.74 (0.43)	0.65 (0.93)	-0.000018 (0.82)
Income	Lower-income	3.22 (0.86)	-16.64 (0.41)	-0.004 (0.74)	-3.19 (0.86)	2.10 (0.92)	0.0000028 (0.82)
	Medium income	**45.22 (<0.0001)**	**82.15 (<.0001)**	**0.04 (<.0001)**	**18.60 (0.049)**	**44.04 (0.001)**	**0.000052 (<0.0001)**
	High income	**77.95 (0.0002)**	**85.40 (0.0004)**	**0.03 (0.002)**	59.88 (0.12)	59.68 (0.12)	0.000023 (0.20)
Marriage	Married	**31.65 (0.02)**	**39.60 (0.008)**	**0.02 (0.02)**	**13.19 (0.45)**	**17.98 (0.30)**	**0.000024 (0.03)**
	Widowed	116.28 (0.43)	-108.63 (0.62)	0.003 (0.79)	-58.22 (0.84)	6.51 (0.99)	-0.000004 (0.84)
	Divorced	31.50 (0.15)	22.66 (0.33)	0.008 (0.19)	33.81 (0.01)	47.27 (0.03)	0.000011 (0.32)
Body weight	Normal weight	**76.79 (0.046)**	**107.12 (0.0004)**	**0.04 (<0.0001)**	15.94 (0.69)	36.71 (0.12)	0.0000011 (0.20)
	Over-weight	36.25 (0.26)	29.70 (0.48)	0.002 (0.87)	19.53 (0.58)	41.30 (0.21)	0.000010 (0.56)
	Obese	54.17 (0.31)	-28.82 (0.65)	0.002 (0.87)	31.56 (0.65)	23.52 (0.29)	0.000001 (0.94)
Diabetes	Having diabetes	90.94 (0.03)	89.45 (0.07)	0.01 (0.19)	29.34 (0.62)	10.62 (0.78)	0.0000041 (0.67)
	Not having diabetes	**47.13 (0.23)**	**55.03 (0.16)**	**0.03 (0.007)**	**85.16 (0.10)**	**135.53 (0.04)**	**0.000047 (0.02)**
Hypertension	No hypertension	**85.77 (0.0002)**	**89.38 (<0.0001)**	**0.04 (0.0003)**	**59.58 (0.03)**	**73.42 (0.01)**	**0.000038 (0.03)**
	Having hypertension	-43.86 (0.42)	14.63 (0.87)	-0.009 (0.49)	-132.60 (0.01)	-79.95 (0.53)	-0.000021 (0.31)
Depression	No Depression	**76.75 (0.04)**	**99.59 (0.04)**	**0.02 (0.01)**	21.61 (0.70)	57.87 (0.33)	0.000017 (0.12)
	Depression	40.85 (0.06)	13.90 (0.62)	0.006 (0.53)	7.34 (0.19)	-4.99 (0.54)	0.0000058 (0.57)
Sleep duration	<=6 hours	61.54 (0.01)	61.05 (0.06)	0.01 (0.28)	54.62 (0.42)	14.95 (0.69)	0.0000026 (0.88)
	6-9 hours	**40.43 (1.12)**	**44.81 (0.11)**	**0.02 (0.03)**	**17.92 (0.53)**	**37.98 (0.22)**	**0.000026 (0.04)**
	>9 hours	24.73 (0.10)	31.26 (0.17)	0.0003 (0.42)	72.37 (<0.0001)	-101.70 (0.020)	**-0.000017 (0.047)**
Sleep disturbance	Sleep disturbance	57.18 (0.21)	-52.50 (0.35)	-0.006 (0.70)	22.03 (0.55)	-20.56 (0.61)	-0.000014 (0.39)
	No sleep disturbance	**45.51 (0.04)**	**73.08 (0.0004)**	**0.02 (<0.0001)**	**14.27 (0.51)**	**41.02 (0.046)**	**0.000030 (0.001)**

1. LSMEANS: Least-squares means of the different measurements of cognitive function.

2. P-value: it indicates whether the association is statistically significant or not among the different levels of physical activity measurement, with the lowest level as the reference group.

3. Peak-30 (MIMS/min) was classified as “MIMS/min≤31.5897 (ref)”, “31.5897≤MIMS/min≤40.4608” and “MIMS/min>15349.99”, as level1, level2, and level3.

4. Daily physical activity (MIMS/d) was classified as “MIMS/d≤7574.19 (ref)”, “9574.19≤MIMS/d≤15349.99” and “MIMS>15349.99”, as level1, level2 and level3.

5. The covariates that have been adjusted in the model include body weight, hypertension, diabetes, sleep duration, smoking, depression, age, gender, race, education, marriage, income, alcohol, occupation, money management, watching movies, participation of social events

**Table 2 (abstract P18).** The quantile-based association between Peak-30 MIMS/min and cognition in different subgroups

Demographic characters			Global Z score for cognition at 10% quantile	Global Z score for cognition at 25% quantile	Global Z score for cognition at 50% quantile	Global Z score for cognition at 75% quantile	Global Z score for cognition at 90% quantile
			β(P-value)	β(P-value)	β(P-value)	β(P-value)	β(P-value)
Adjust for covariates	Peak-30 (MIMS/min)		**0.31 (0.003)**	**0.23 (0.001)**	**0.18 (<0.0001)**	**0.11(0.01)**	**0.10 (0.01)**
	Daily physical activity (MIMS/d)		-0.018 (0.90)	0.16 (0.03)	0.10 (0.07)	0.09 (0.06)	0.02 (0.70)
Age groups	Peak-30	60-69 years old	**0.23 (0.02)**	**0.17 (0.05)**	0.06 (0.38)	0.06 (0.20)	0.09 (0.10)
	Daily PA		**0.25 (0.04)**	0.14 (0.10)	0.02 (0.78)	0.04 (0.52)	0.01(0.86)
	Peak-30	70-80 years old	0.08 (0.80)	0.23 (0.37)	0.17 (0.39)	0.03 (0.85)	-0.06 (0.56)
	Daily PA		-0.18 (0.57)	-0.07 (0.78)	-0.02 (0.91)	0.03 (0.79)	0.02 (0.79)
	Peak-30	≥ 80 years old	0.27 (0.80)	0.27 (0.79)	0.93 (0.25)	0.91 (0.17)	0.91 (0.17)
	Daily PA		-0.21 (0.82)	-0.21 (0.80)	0.58 (0.39)	0.83 (0.19)	0.83 (0.13)
Racial groups	Peak-30	Hispanics	**0.61 (0.001)**	**0.52 (0.001)**	**0.45 (0.0002)**	**0.43 (0.001)**	**0.43 (<0.0001)**
	Daily PA		0.09 (0.82)	0.50 (0.19)	0.35 (0.36)	0.01 (0.97)	0.28 (0.33)
	Peak-30	Non-Hispanic White	0.12 (0.47)	**0.25 (0.02)**	**0.19 (0.01)**	0.09 (0.19)	0.02 (0.7)
	Daily PA		-0.08 (0.64)	0.13 (0.14)	0.07 (0.29)	0.05 (0.36)	0.01 (0.78)
	Peak-30	Non-Hispanic Black	-0.08 (0.77)	-0.10 (0.69)	-0.14 (0.48)	-0.23 (0.15)	-0.28 (0.07)
	Daily PA		0.26 (0.43)	0.05 (0.86)	-0.07 (0.71)	-0.02 (0.89)	0.01 (0.93)
	Peak-30	Non-Hispanic Asian	-0.23 (0.41)	-0.23 (0.42)	0.04 (0.88)	0.06 (0.80)	0.06 (0.76)
	Daily PA		0.16 (0.52)	0.16 (0.56)	0.31 (0.25)	0.26 (0.30)	0.26 (0.28)
Genders	Peak-30	Male	0.21 (0.11)	0.13 (0.23)	0.15 (0.08)	0.06 (0.42)	0.19 (0.01)
	Daily PA		0.11 (0.55)	0.18 (0.16)	0.13 (0.21)	0.05 (0.50)	0.12 (0.06)
	Peak-30	Female	0.002 (0.99)	0.20 (0.07)	**0.14 (0.02)**	0.06 (0.22)	**0.10 (0.02)**
	Daily PA		-0.02 (0.91)	0.08 (0.45)	0.07 (0.27)	0.001 (0.98)	0.06 (0.21)
Education	Peak-30	Less than 9th grade -9-11th grade	0.80 (0.12)	**0.80 (0.03)**	0.46 (0.22)	0.27 (0.53)	0.27 (0.49)
	Daily PA		-1.07 (0.20)	-1.07 (0.23)	0.17 (0.83)	0.11 (0.88)	0.11 (0.87)
	Peak-30	High school	0.19 (0.50)	-0.06 (0.77)	0.06 (0.74)	-0.031 (0.82)	0.003 (0.98)
	Daily PA		-0.23 (0.24)	-0.30 (0.08)	0.12 (0.41)	0.08 (0.43)	0.14 (0.17)
	Peak-30	Some colleges and above	**0.45 (<0.0001)**	**0.23 (0.001)**	**0.19 (0.002)**	0.07 (0.16)	0.08 (0.04)
	Daily PA		**0.31 (0.02)**	**0.22 (0.003)**	0.12 (0.06)	0.08 (0.18)	0.06 (0.31)
Occupation	Peak-30	Having occupation	**0.36 (0.045)**	0.09 (0.48)	**0.14 (0.047)**	0.08 (0.20)	0.11 (0.07)
	Daily PA		**0.34 (0.049)**	0.26 (0.05)	0.10 (0.19)	0.07 (0.28)	0.09 (0.20)
	Peak-30	Not having occupation	**0.22 (0.03)**	**0.18 (0.02)**	**0.19 (0.001)**	**0.12 (0.02)**	**0.12 (0.003)**
	Daily PA		0.002 (0.99)	**0.16 (0.04)**	0.13 (0.10)	0.04 (0.58)	0.03 (0.53)
Smoking	Peak-30	Smoking	0.29 (0.05)	0.21 (0.07)	0.11 (0.17)	0.09 (0.18)	0.04 (0.54)
	Daily PA		0.002 (0.99)	0.18 (0.08)	0.09 (0.20)	0.08 (0.23)	0.02 (0.74)
	Peak-30	Non-smoking	**0.49 (<0.0001)**	**0.22 (0.03)**	0.14 (0.06)	0.11 (0.09)	0.06 (0.28)
	Daily PA		0.25 (0.12)	0.04 (0.74)	0.09 (0.32)	-0.006 (0.94)	0.02 (0.79)
Alcohol	Peak-30	Drinking	**0.22 (0.05)**	**0.30 (0.001)**	**0.19 (0.01)**	**0.20 (0.01)**	**0.16 (0.02)**
	Daily PA		**0.60 (0.001)**	0.25 (0.05)	0.10 (0.29)	0.04 (0.65)	0.09 (0.28)
	Peak-30	Non-drinking	0.19 (0.18)	0.04 (0.66)	**0.15 (0.03)**	0.03 (0.50)	0.04 (0.34)
	Daily PA		-0.16 (0.27)	0.02 (0.81)	0.09 (0.17)	-0.02 (0.72)	-0.02 (0.67)
Social participation	Peak-30	Participation	**0.26 (0.002)**	**0.25 (<0.0001)**	**0.18 (<0.0001)**	**0.10 (0.02)**	**0.10 (0.03)**
	Daily PA		0.16 (0.18)	**0.21 (0.01)**	0.10 (0.08)	0.09 (0.10)	0.04 (0.47)
	Peak-30	Non-participation	-0.42 (0.57)	-0.42 (0.56)	-0.26 (0.69)	-0.004 (1.00)	-0.004 (0.99)
	Daily PA		-0.15 (0.77)	-0.15 (0.77)	0.09 (0.88)	0.03 (0.97)	0.03 (0.97)
Income	Peak-30	Lower-income	-0.42 (0.58)	-0.34 (0.59)	-0.55 (0.42)	-0.79 (0.24)	-0.79 (0.26)
	Daily PA		0.04 (0.92)	0.29 (0.53)	-0.36 (0.45)	-0.42 (0.40)	-0.42 (0.44)
	Peak-30	Medium income	-0.09 (0.54)	0.04 (0.71)	0.13 (0.13)	0.14 (0.04)	0.10 (0.08)
	Daily PA		-0.18 (0.17)	0.08 (0.43)	0.02 (0.81)	0.01 (0.87)	-0.04 (0.41)
	Peak-30	High income	**0.43 (0.001)**	0.14 (0.19)	**0.21 (0.01)**	0.09 (0.07)	0.02 (0.69)
	Daily PA		**0.35 (0.049)**	0.20 (0.14)	**0.24 (0.01)**	**0.15 (0.01)**	0.13 (0.10)
Marriage	Peak-30	Married or living with a partner	**0.36 (0.001)**	**0.18 (0.04)**	**0.16 (0.02)**	0.06 (0.30)	0.04 (0.41)
	Daily PA		**0.26 (0.03)**	**0.19 (0.02)**	**0.18 (0.03)**	0.06 (0.38)	0.03 (0.64)
	Peak-30	Widowed	-0.03 (0.93)	0.04 (0.91)	0.21(0.49)	0.02 (0.93)	-0.02 (0.95)
	Daily PA		-0.05 (0.87)	-0.25 (0.39)	0.12 (0.59)	0.007 (0.97)	-0.007 (0.96)
	Peak-30	Divorced or separated	-0.66 (0.08)	-0.17 (0.56)	0.10 (0.60)	0.14 (0.23)	0.02 (0.84)
	Daily PA		-0.38 (0.19)	-0.12 (0.57)	0.08 (0.56)	0.08 (0.39)	0.07 (0.46)
	Peak-30	Never married	-0.33 (0.56)	-0.33 (0.56)	-0.33 (0.53)	-0.15 (0.79)	-0.15 (0.79)
	Daily PA		-0.28 (0.60)	-0.28 (0.58)	-0.27 (0.56)	-0.13 (0.81)	-0.13 (0.81)
Bodyweight	Peak-30	Normal weight	**0.36 (0.08)**	**0.28 (0.03)**	**0.25 (0.01)**	**0.19 (0.02)**	**0.14 (0.09)**
	Daily PA		-0.38 (0.15)	0.04 (0.87)	0.18 (0.20)	0.18 (0.05)	0.08 (0.31)
	Peak-30	Over-weight	0.28 (0.29)	0.01 (0.94)	0.26 (0.02)	0.23 (0.01)	0.18 (0.07)
	Daily PA		-0.01 (0.94)	-0.004 (0.97)	0.15 (0.13)	0.10 (0.25)	0.05 (0.53)
	Peak-30	Obese	**0.27 (0.02)**	0.05 (0.65)	0.05 (0.62)	0.02 (0.77)	-0.04 (0.56)
	Daily PA		0.13 (0.38)	0.13 (0.23)	0.06 (0.51)	0.07 (0.39)	-0.02 (0.72)
Diabetic status	Peak-30	Having diabetes	**0.54 (0.02)**	0.25 (0.20)	0.20 (0.11)	**0.22 (0.02)**	**0.19 (0.04)**
	Daily PA		-0.41 (0.22)	-0.16 (0.59)	0.10 (0.59)	0.17 (0.24)	-0.08 (0.54)
	Peak-30	Not having diabetes	0.15 (0.16)	**0.17 (0.04)**	**0.13 (0.02)**	0.07 (0.11)	0.02 (0.60)
	Daily PA		0.19 (0.15)	0.07 (0.39)	0.05 (0.43)	0.04 (0.38)	0.03 (0.58)
Hypertension	Peak-30	No hypertension	**0.40 (0.01)**	**0.23 (0.04)**	0.10 (0.27)	0.09 (0.24)	0.06 (0.41)
	Daily PA		0.12 (0.36)	0.20 (0.08)	0.14 (0.12)	**0.17 (0.04)**	0.07 (0.31)
	Peak-30	Having hypertension	**0.36 (0.04)**	0.21 (0.14)	0.20 (0.10)	**0.23 (0.03)**	0.12 (0.19)
	Daily PA		-0.22 (0.16)	0.13 (0.22)	0.001 (0.98)	0.05 (0.33)	0.04 (0.39)
Depression	Peak-30	No Depression	**0.22 (0.03)**	**0.19 (0.004)**	**0.16 (<0.0001)**	**0.13 (0.01)**	**0.11 (0.02)**
	Daily PA		-0.15 (0.36)	0.10 (0.24)	0.07 (0.30)	0.09 (0.12)	-0.01 (0.79)
	Peak-30	Depression	-0.0019 (0.99)	0.15 (0.44)	0.03 (0.88)	0.09 (0.54)	0.05 (0.64)
	Daily PA		0.32 (0.08)	0.22 (0.20)	0.08 (0.59)	0.05 (0.62)	0.07 (0.35)
Sleep duration	Peak-30	<=6 hours	0.21 (0.15)	0.04 (0.74)	0.04 (0.69)	0.05 (0.56)	-0.03 (0.45)
	Daily PA		0.06 (0.71)	0.07 (0.50)	0.02 (0.74)	0.003 (0.96)	-0.01 (0.85)
	Peak-30	6-9 hours	**0.37 (0.001)**	**0.22 (0.0004)**	**0.20 (<0.0001)**	**0.16 (0.0002)**	**0.13 (0.02)**
	Daily PA		0.07 (0.67)	0.17 (0.08)	**0.17 (0.03)**	**0.17 (0.01)**	0.12 (0.06)
	Peak-30	>9 hours	**0.77 (0.01)**	**0.54 (0.06)**	**0.55 (0.07)**	**0.55 (0.06)**	**0.49 (0.07)**
	Daily PA		**0.84 (0.045)**	0.39 (0.28)	0.39 (0.33)	0.37 (0.29)	0.37 (0.31)
Sleep disturbance	Peak-30	Sleep disturbance	0.21 (0.52)	0.16 (0.40)	0.07 (0.53)	0.07 (0.39)	0.03 (0.71)
	Daily PA		0.03 (0.89)	0.08 (0.61)	0.07 (0.40)	-0.004 (0.94)	-0.12 (0.08)
	Peak-30	No sleep disturbance	0.13 (0.07)	**0.19 (0.002)**	**0.17 (0.001)**	**0.11 (0.01)**	**0.14 (0.003)**
	Daily PA		0.13 (0.35)	0.15 (0.12)	0.14 (0.06)	**0.11 (0.04)**	0.09 (0.12)

### Supplementary Information

**Additional file 1.** National Big Data Health Science Conference 2023 (February 10-11).

