# Supplementary material for: Proceedings of the 4th National Big Data Health Science Conference
Source: BMC Proc. 2023 Nov 23;17(Suppl 19):32. doi: 10.1186/s12919-023-00281-y (PMC10666299; doi:10.1186/s12919-023-00281-y)
Supplement: Supplementary file 1 — Additional file 1. National Big Data Health Science Conference 2023 (February 10-11). [file 12919_2023_281_MOESM1_ESM.docx]

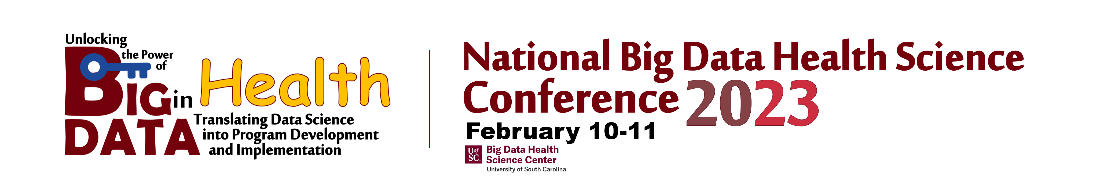


**DAY 1: FRIDAY, FEBRUARY 10**

**Registration & Breakfast | Ballroom 2**

8:00am – 9:30am

**Plenary Sessions | Ballroom 3**

***Opening & Welcoming Remarks***

9:40am – 9:55am

Michael Amiridis, President, University of South Carolina

***Announcement of Case Competition Awards***

Thomas Chandler, Dean, Arnold School of Public Health, University of South Carolina

***Creating a FAIR and Equitable Data Ecosystem***

10:00am – 10:45am

Susan Gregurick (National Institutes of Health)

***Leveraging the National COVID Cohort Collaborative (N3C), a National Electronic Health Records Repository, for Insights into COVID-19 and Health Equity***

10:55pm – 11:40am

Pena Patel (University of Washington)

***The Macroscope Meets the Microscope: Integrating Earth Science Data with Disease Surveillance for Outbreak Forecasting***

11:45am – 12:30pm

Michael Wimberly (University of Oklahoma)

**Lunch | Ballroom 2**

12:30pm – 1:30pm

***Underrepresented Minorities Academic Career Development & Data Science Applied to Health Luncheon (Held in Ballroom 1A by Invitation Only)***

Julian R. Williams, Vice President for Diversity, Equity and Inclusion, University of South Carolina
Cheryl L. Addy, Vice Provost and Dean of Faculty, Interim Executive Director of OIRAA, Interim Dean of Graduate School, University of South Carolina

**Breakout Sessions (S1-S3)**

1:30pm – 3:30pm

**S1. Genomic Core| Ballroom 1A**

**The Power of Genomic Analysis in Biological & Health-Related Data**

Moderator: Homayoun Valafar (University of South Carolina)

***Rigorous Benchmarking of T Cell Receptor Repertoire Profiling Methods for Cancer RNA Sequencing***

Serghei Mangul (University of Southern California)

***Big Data and Deep Learning-Based Genome Mining for Drug Discovery***

Jie Li (University of South Carolina)

***Integrated Molecular and Clinical Data Analysis of Cancer Patients***

Anna Blenda (University of South Carolina, Greenville)

***Kynurenine Aminotransferase II Inhibition Improves Sleep Architecture in Adult Male and Female Rats Exposed to Kynurenic Acid Elevation During Development***

Snezana Milosavljevic (University of South Carolina)

**S2. Geospatial Core| Ballroom 1B**

**Seeing What Others Can’t: Integrating Geospatial Methods into Big Data Research**

Moderator: Melissa Nolan (University of South Carolina)

***Employing Big Data Methods to Elucidate the Impact of Three Decade’s Extreme Weather Events on Early Childhood Development in the United States***

Leila Larson (University of South Carolina)

***The Utility of a Bayesian Predictive Model to Forecast Neuroinvasive West Nile Virus Disease in the United States, 2022***

Maggie McCarter (University of South Carolina)

***Using Geographically Weighted Poisson Regression to Examine Factors Associated with the Spatial Heterogeneity of COVID-19 Infection Rate in Bologna, Italy***

Addisu Jember Zeleke (University of Bologna)

***Geographic Variations in Spatial Access to Hospital Maternity Care and Digital Access to Technology in the United States***

Peiyin Hung (University of South Carolina)

***Adapting Survey Designs for Vector Surveillance using Bayesian Decision Theory: An Application to an Ongoing Tick Monitoring Program in the Southeastern United States***

Brendan Case (University of Vermont)

***Identifying Associations Between Hotspots of Social Determinants of Health Needs, Community-Based Organizations and Healthcare Resource Use***

Reid DeMass (University of South Carolina)

***Using Smartphone-Based Place Visitation Big Data to Improve Health Measure Estimation***

Huan Ning (University of South Carolina)

***Drones for 3D Monitoring of Coastal Ecosystem Healthiness with Sea Level Rise***

Cuizhen Wang (University of South Carolina)

**S3. Electronic Health Records Core I | Ballroom 3**

**Cutting Edge Methodologies to Handle Health Science Big Data**

Moderator: Jiajia Zhang (University of South Carolina)

***Oracle P-Values for High Dimensional Regression Models***

Helen Zhang (University of Arizona)

***Distribution Invariant Differential Privacy***

Xiaotong Shen (University of Minnesota)

***Leveraging Electronic Health Records for Big Data Research***

Jihad Obeid (Medical University of South Carolina)

***On the Use of an Automated, Reproducible Binning Approach to Bring Consistency in Calibration of Predictive Models Built on Electronic Health Records***

Madhusree Chaudhurry (Health Data Analytics Institute)

***Topological Inference on Heat-Diffusion Estimates of Persistence Diagrams***

Yuan Wang (University of South Carolina)

**Breakout Sessions (S4-S6)**

3:45-5:45pm

**S4. NIH Trainee Session| Ballroom 1A**

Moderator: Jiajia Zhang (University of South Carolina)

***Antimicrobial use in the Outpatient Setting During COVID-19 Pandemic***

Pamela Bailey (University of South Carolina)

***Quantifying Vascular Calcification and Predicting Patient Outcomes with Synthetic Data, Deep Neural Networks, and Logic Programming***

Forest Agostinelli (University of South Carolina)

***Informatics Approach to Identification and Deep Phenotyping of PASC Cases***

Chen Liang (University of South Carolina)

***Informatics Leveraging Big Data for the Prevention and Control of Sexually Transmitted Infections (STIs)***

Mufaro Kanyangarara (University of South Carolina)

***Graduate Student Mentoring in Quantitative Health Sciences: A Mentee’s Perspective***

Joshua Miles (University of Florida)

**S5. AI for Sensing & Diagnosis Core| Ballroom 1B**

**AI for Medical Research & Treatment**

Moderators: Chris Sutton & Qian Wang (University of South Carolina)

***Using Random Forest Classifier to Identify Important COVID-19 Patient Characteristics Predicting Mortality in South Florida***

Debarshi Datta (Florida Atlantic University)

***Prognostic Models for Sepsis Based on Short-Term ICU Data***

Chunyan Li (University of South Carolina)

***Interrogating the Metabolomic Profile of Amyotrophic Lateral Sclerosis in the Post-Mortem Human Brain by Infrared Matrix-Assisted Laser Desorption Electrospray Ionization (IR-MALDESI) Mass Spectrometry Imaging (MSI)***

Alexandria Sohn (North Carolina State University)

***Leveraging Smartphone Technology to Enhance Patient-Centered HIV Care and Treatment Services in Nigeria’s Most Populous City: The Jolly-95 App Experience***

Obioma Azurunwa (Centre for Integrated Health Programs)

***Exploring Racial Disparities in Colorectal Polyp Characteristics at Screening Colonoscopy using Machine Learning Approaches***

Yuqi Wu (Mayo Clinic Department of Artificial Intelligence & Informatics)

***Using the Semantic Web Technology to Leverage Interoperable Clinical Decision Support System (CDSS) Rules—A Pathway to Interoperable Patient Records***

Xia Jing (Clemson University)

***Using Label-Free Two-Photon Microscopy and Deep Learning Image Processing to Assess the Chondrocyte Viability of Articular Cartilage***

Hongming Fan (Clemson University)

***An Algorithm on the Concept of the Uniquely Most Frequently Visited Provider***

Songyuan Deng (University of South Carolina)

**S6. Social Media Core| Ballroom 3**

**Harnessing Big Social Data for Public Health Research**

Moderators: Zhenlong Li & Shan Qiao (University of South Carolina)

***Using Social Media and Place Visitation Data for Public Health Research: Applications, Challenges, and Innovation Opportunities***

Zhenlong Li (University of South Carolina)

***Epitweetr: Development and Implementation of an AI Tool to Monitor Twitter Trends for Early Warning of Threats to Public Health***

Laura Espinosa (European Centre for Disease Prevention and Control)

***Using Artificial Intelligence Methods to Identify Perinatal Substance Use Discourse and Stigma Patterns on Twitter***

Dezhi Wu (University of South Carolina)

***Examine HIV Service Interruption During the COVID-19 Pandemic using Cellphone-Based Place Visitation Data***

Shan Qiao (University of South Carolina)

***Assessing Information Quality and User Engagement of Eating Disorder Content on TikTok***

Valerie Lookingbill (University of South Carolina)

***Youth-Focused Tobacco Prevention through a Novel Advertising, Sales, and Social Media Tracking Dashboard***

Jaron King (S.C. Department of Health and Environmental Control)

***Disparities in Mental Health Service Utilization Among Immigrants in the U.S. using Geospatial Big Data***

Fengrui Jing (University of South Carolina)

**DAY 2: SATURDAY, FEBRUARY 11**

**Registration & Breakfast | Ballroom 2**

8:00am – 9:30am

**Poster Session I | Ballroom Prefunction**

8:30am – 9:30am

**Plenary Sessions | Ballroom 3**

***Harnessing Big Heterogeneous Data to Evaluate the Potential Impact of HIV Responses Among Key Populations in Generalized Epidemic Settings in Sub Saharan Africa: The Boloka Data Warehouse Project***

9:35am – 10:20am

Refilwe Nancy Phaswana-Mafuya (University of Johannesburg)

***Priorities, Strategies, and Research Interests in Data Science for Health: Perspectives from NIH Leadership and Programs***

10:25am – 11:10am

Lori Scott-Sheldon (National Institutes of Health)

***Creating Four Methodological Themes in Spatial Health Science***

11:15am – 12:00pm

Fahui Wang (Louisiana State University)

**Lunch | Ballroom 2 & Poster Session II | Ballroom Prefunction**

12:00pm – 1:00pm

**Breakout Sessions (S7-S9)**

1:00-3:00pm

**S7. Developing a Taxonomic Framework for Ethics & Biases in Data Analytics Involving Electronic Health Records & Other Big Data Sources| Ballroom 1**

Moderator: Bankole Olatosi (University of South Carolina)

***Race and Risk: A Bioinformatic Analysis of Alzheimer’s Disease***

Loni Taylor (Meharry Medical College)

***Ethics Framework Tool Charette Session for Electronic Health Records***

George Khushf & Shan Qiao (University of South Carolina)

**S8. Electronic Health Records Core II| Ballroom 3**

**Addressing Public Health Issues using Health Science Big Data**

Moderator: Jiajia Zhang (University of South Carolina)

***SARS-CoV-2 Infections and Severe Maternal Morbidity in U.S. Patients: A National Retrospective Cohort Study***

Jihong Liu (University of South Carolina)

***Leveraging the Continuity in Treatment Dashboard Analytics to Retain Persons Living with HIV on ART Care and Treatment in Nigeria- The Lagos ART Surge Experience***

Oluwasola Idowu (Centre for Integrated Health Programs)

***Social Determinants of Health and Severe Maternal Morbidity During the COVID-19 Pandemic in the U.S.***

Peiyin Hung (University of South Carolina)

***Identifying Risk Factor Clusters to Predict Undiagnosed Cognitive Loss***

Longgang Zhao (University of South Carolina)

***The Incidence and Evolving Risk Factors of Diabetes Among People Living with HIV- A Population-Based Cohort Study***

Gazi Sakir Mohammad Pritom (University of South Carolina)

***Prediction of Low Birthweight Babies and Associated Risk Factors in an Extremely Imbalanced Large-Scale Prenatal Care Dataset: A Machine Learning Approach***

Yang Ren (University of South Carolina)

***Association Between Patient-Provider Shared Decision-Making and Use of Pain-Related Complementary and Integrative Health Modalities Among Adults with Chronic Noncancer Pain, 2010-2017***

Yi-Wen Shih (University of South Carolina)

**S9. Creating Big Data Dashboards using SAS Viya for Learners| USC Discovery Building, 431 (Gather in Lobby of the Alumni Center at 12:45pm)**

Linda Jordan (SAS)

**Plenary Sessions | Ballroom 3**

***Advancing Digital Health Policies and Funding Strategies to Improve the Public’s Health***

3:15pm – 4:00pm

Valerie Rogers (Healthcare Information and Management Systems Society)

***Announcement of Abstract Awards***

4:00pm – 4:05pm

***Closing Remarks***
4:05-4:15
Julius Fridriksson, Vice President for Research, University of South Carolina
